# Optimization of Peptide Linker-Based Fluorescent Ligands for the Histamine H_1_ Receptor

**DOI:** 10.1021/acs.jmedchem.2c00125

**Published:** 2022-06-03

**Authors:** Zhi Yuan Kok, Leigh A. Stoddart, Sarah J. Mistry, Tamara A. M. Mocking, Henry F. Vischer, Rob Leurs, Stephen J. Hill, Shailesh N. Mistry, Barrie Kellam

**Affiliations:** † Division of Biomolecular Science and Medicinal Chemistry, School of Pharmacy, University of Nottingham Biodiscovery Institute, University Park, Nottingham NG7 2RD, U.K.; ‡ Division of Physiology, Pharmacology & Neuroscience, Medical School, School of Life Sciences, University of Nottingham, Nottingham NG7 2UH, U.K.; § Centre of Membrane Proteins and Receptors, University of Birmingham and University of Nottingham, the Midlands, Nottingham NG7 2UH, U.K.; ∥ Amsterdam Institute for Molecules, Medicines and Systems, Division of Medicinal Chemistry, Faculty of Science, Vrije Universiteit Amsterdam, De Boelelean 1083, 1083 HV Amsterdam, The Netherlands

## Abstract

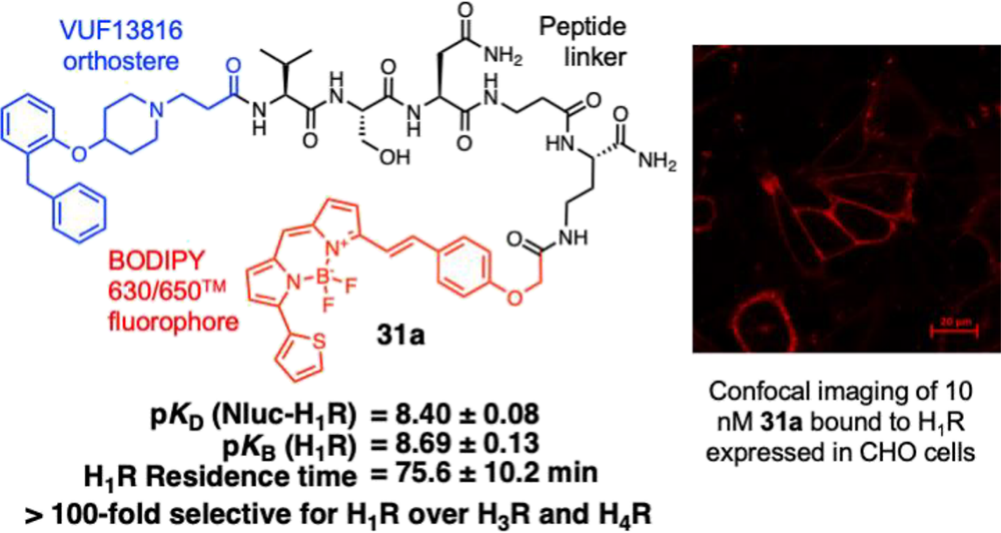

The histamine H_1_ receptor (H_1_R) has recently been implicated in mediating cell proliferation and cancer progression; therefore, high-affinity H_1_R-selective fluorescent ligands are desirable tools for further investigation of this behavior in vitro and in vivo. We previously reported a H_1_R fluorescent ligand, bearing a peptide-linker, based on antagonist VUF13816 and sought to further explore structure–activity relationships (SARs) around the linker, orthostere, and fluorescent moieties. Here, we report a series of high-affinity H_1_R fluorescent ligands varying in peptide linker composition, orthosteric targeting moiety, and fluorophore. Incorporation of a boron-dipyrromethene (BODIPY) 630/650-based fluorophore conferred high binding affinity to our H_1_R fluorescent ligands, remarkably overriding the linker SAR observed in corresponding unlabeled congeners. Compound **31a**, both potent and subtype-selective, enabled H_1_R visualization using confocal microscopy at a concentration of 10 nM. Molecular docking of **31a** with the human H_1_R predicts that the optimized peptide linker makes interactions with key residues in the receptor.

## Introduction

The histamine H_1_ receptor (H_1_R), a class A G protein-coupled receptor (GPCR), is one of four histamine receptor subtypes found in humans. The H_1_R predominantly couples to Gα_q/11_ proteins and upon receptor activation progresses mainly through the inositol-phospholipid-dependent pathway with a subsequent increase in intracellular Ca^2+^ concentration. The H_1_R is widely distributed throughout the body, particularly in immune cells, the lungs, blood vessels, and the central nervous system.^1,2^ The H_1_R has an established role in allergy and inflammation and is the therapeutic target for antihistamines (e.g., loratadine and cetirizine) in the treatment of allergic rhinitis, hayfever, and urticaria. There is however a growing body of evidence suggesting that histamine receptors, including the H_1_R, play an important role in cancer progression due to their involvement in cell proliferation and tumor growth.^3,4^ Activation of histamine receptors has been shown to both promote or suppress tumor growth depending on the tumor microenvironment, histamine metabolism, the receptor subtypes involved, and the local histamine receptor balance.^3−6^ Given the complexity of histamine cancer pharmacology, a better understanding of H_1_R pharmacology in various tumor microenvironments could facilitate the development of H_1_R-targeted cancer therapeutics.^7−9^

**Figure 1:**
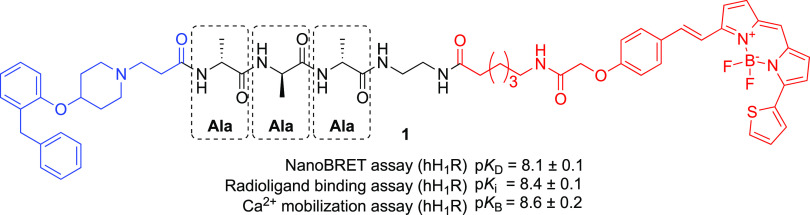
Structure of the previously reported H_1_R fluorescent ligand **1** consisting of the VUF13816 fragment-derived orthostere (blue) connected to a BODIPY630/650-X fluorophore (red) by an Ala-Ala-Ala tripeptide linker and its associated binding affinities.^25^

Fluorescent ligands for GPCRs are synthesized by attaching a fluorophore to a receptor-targeting moiety or “orthostere” (an agonist or antagonist ligand) for the receptor of interest. A linker is usually necessary to separate the fluorophore from the orthostere to avoid disruption of the key ligand–target interactions that occur in the parent ligand binding pocket.^10−13^ Fluorescent ligands have proven themselves to be valuable tools in the study of GPCR pharmacology as they can provide insight into ligand–receptor binding and kinetics,^13−25^ receptor localization and internalization,^20,21,26^ organization and oligomerization^27−34^ at the single-cell level using primarily confocal microscopy, and resonance-energy-transfer techniques, both in vitro and in vivo.^35,36^ In addition, it has been recently demonstrated that fluorescent ligands or small molecule ligands in general could be utilized to fluorescently label endogenous GPCRs expressed in living tissues using ligand-directed chemistry.^37−39^ As such, fluorescent ligands bear the potential to probe receptor behavior, expression, density, and organization in primary cell cultures derived from specific cancers. To date, several fluorescent ligands for the H_1_R have been reported, many of which presented several issues (e.g., high levels of non-specific binding and cellular uptake) that affected their utility for pharmacological studies.^25,28,40^ Previously, work within our group led to the discovery of the fluorescent ligand **1**(25) (Figure 1), which is based on the antagonist H_1_R ligand VUF13816^41^ consisting of a BODIPY 630/650-X fluorophore and an Ala-Ala-Ala tripeptide linker. Compound **1** demonstrated improved physicochemical and imaging properties compared to a previously reported mepyramine-based fluorescent ligand,^28^ which were attributed to the incorporation of a peptide linker.^25^ Other work within our group has demonstrated the peptide linker strategy to be successful in converting a non-selective adenosine receptor antagonist into a high-affinity adenosine A_3_ receptor-selective fluorescent probe.^42^

Recent advances in GPCR structural biology have provided a platform for more rational design of drugs targeting this protein class, and we reasoned that this could also be extended to the rational design of fluorescent ligands for studying GPCR pharmacology.^43^ The crystal structure of the antagonist doxepin bound to the human H_1_R (PDB code: 3RZE) was published in 2011 and revealed several basic residues at the entrance of the binding pocket, namely, K179^ECL2^ (Ballesteros–Weinstein nomenclature^44^), K191^5.39^, and H450^7.35^. It was proposed that these residues played a role in interacting with the carboxylic acid group present in second-generation zwitterionic antihistamines (e.g., cetirizine and acrivastine).^45^ We were attracted to the possibility of synthesizing high-affinity H_1_R-selective fluorescent ligands through targeting these residues, in addition to exploring additional receptor–ligand interactions by probing the outer vestibule of the receptor binding pocket. We envisioned that this could be achieved by optimizing the composition of the peptide-linker moiety to engage in binding interactions with residues lining the ligand entry route when the fluorescent ligand is bound to the receptor. The detection sensitivity offered using high affinity fluorescent ligands opens up the potential for their use in physiological and diseased tissue (which exhibit much lower cell surface receptor expression compared to engineered cell lines in both in vitro and in vivo paradigms). Furthermore, this could be achieved with very low concentrations of fluorescent ligand, thus reducing non-specific binding and improving the signal-to-noise ratio.

In this study, we report the design, synthesis, and pharmacological characterization of a series of high-affinity H_1_R fluorescent ligands comprising a peptide linker of varying composition and explore two orthosteres. The fluorophores were attached to the congeners, utilizing either copper-catalyzed alkyne-azide cycloaddition (CuAAC) or amide coupling. Our efforts led to the discovery of **31a**, which exhibits nanomolar affinity at the H_1_R, is >100-fold selective for H_1_R over the H_3_R and H_4_R subtypes, and provides the capability to visualize cells at a low ligand concentration (10 nM). Although the linker SAR was established in the unlabeled congeners, further investigation revealed that the nature of the fluorophore itself plays a significant role in determining the overall affinity of our series of fluorescent ligands, which appears to override the linker SAR observed in our library of congeners.

## Results and Discussion

### Chemistry

**Figure 2:**
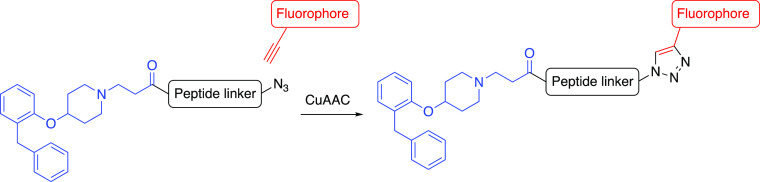
General schematic diagram of fluorescent ligand synthesis using CuAAC for fluorophore conjugation involving an azide-bearing congener and an alkyne-bearing fluorophore.

Our primary objective was to synthesize high-affinity peptide linker-based H_1_R fluorescent ligands by optimizing the linker moiety, using CuAAC for fluorophore conjugation (Figure 2). The use of CuAAC further broadens the scope of this approach by offering orthogonal reaction conditions, allowing incorporation of amino acids into the linker whose side chains (e.g., amine- and carboxylic acid-containing side groups) might otherwise be reactive toward electrophilic fluorophore labeling reagents or interfere with coupling chemistries. This strategy allows us to fully explore peptide linker SARs with minimum side-chain cross-reactivities at the fluorophore conjugation step and achieve high efficiency in overall fluorescent ligand synthesis.

Taking compound **1** as a starting point, we sought to further optimize the composition of the tripeptide linker in the context of a triazole linkage to the fluorophore. We approached this by conducting an initial screening study using unlabeled peptide congeners performed in a systematic manner to determine a more optimal peptide linker. For the purposes of more facile SAR exploration, we chose to emulate the presence of the triazole linking moiety with a phenylalanine residue, serving as a surrogate aryl system uncomplicated by functional reactivity. At the time of the study, the 3-azido-alanine that would ultimately be installed as the terminal linker residue was not commercially available, while the enantiomer 3-azido-d-alanine was. With this in mind, we opted to use d-Phe as a surrogate for the corresponding triazole that would feature in the final fluorescent ligands.

#### Synthesis of Peptide Congeners for Initial Screening

**Scheme 1:**
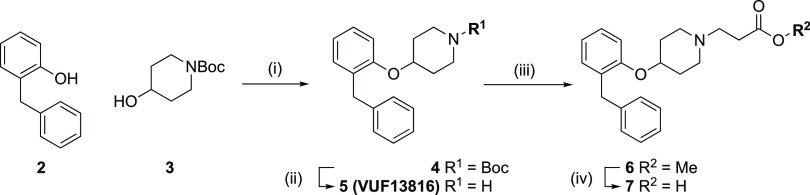
Synthesis of the *N*-(2-Carboxyethyl) Analogue of VUF13816 (7) Reagents and conditions: (i) triphenylphosphine, diisopropyl azodicarboxylate, tetrahydrofuran (THF), rt, 20 h, 42%; (ii) TFA, CH_2_Cl_2_, rt, 17 h, 93%; (iii) methyl acrylate, 1,2-dichloroethane, 70 °C, 4 h, 66%; (iv) NaOH, H_2_O/THF, 0 °C, 5 h, followed by acidification 74%.

Synthesis of the *N*-(2-carboxyethyl) analogue (**7**) of VUF13816 (**5**) was based on procedures previously reported (Scheme 1).^25,46^ Mitsunobu coupling of 2-benzylphenol (**2**) and *N*-Boc-4-hydroxypiperidine (**3**) afforded ether **4**. The latter underwent *N*-Boc deprotection in 2,2,2-trifluoroacetic acid (TFA)/CH_2_Cl_2_ to yield VUF13186 (**5**). Michael addition of **5** to methyl acrylate afforded ester **6**, which underwent saponification in excess NaOH and subsequent acidification to afford the desired orthostere as its free carboxylic acid (**7**).

Congener peptide linker optimization was achieved through a pragmatic, systematic process, whereby the amino acid (AA) at each position (AA^1^, AA^2^, or AA^3^) within the congener was varied while keeping the remaining two positions constant. With the linker present in **1** (AA^1^ = AA^2^ = AA^3^ = Ala) as the starting point for modification, three congener series were synthesized: **8a**–**j** (AA^1^ varied, AA^2^ = AA^3^ = Ala), **9a**–**i** (AA^2^ varied, AA^1^ = Val, AA^3^ = Ala), and **10a**–**i** (AA^3^ varied, AA^1^ = Val, AA^2^ = Ser), which afforded more optimized AA^1^-AA^2^-AA^3^ peptide linker(s). This approach allowed comprehensive exploration of AA functionalities, though it is recognized that the tripeptide sequence may interact with the receptor in a distinct manner to that of its individual component residues. Synthesis was achieved using standard Fmoc solid-phase peptide synthesis (SPPS) procedures on rink amide resin in the presence of 2-(1*H*-benzotriazol-1-yl)-1,1,3,3-tetramethyluronium hexafluorophosphate (HBTU) as the coupling reagent. Each resin-linked tetrapeptide (AA^1^-AA^2^-AA^3^-D-Phe) was then capped with **7** as the final coupling step. The resins were dried and treated with 90% TFA/H_2_O to effect resin cleavage and remove any side-chain protecting groups. The crude mixture was purified by preparative HPLC to afford the product in purity of ⩾95% determined by analytical HPLC, with the exception of **9b** (90%) and **10e** (88%). Resin loading was not determined and SPPS yields were estimated based on a resin loading of 1 mmol/g as reported by the manufacturer. As such, observed yields are likely to be underestimates.

**Scheme 2:**
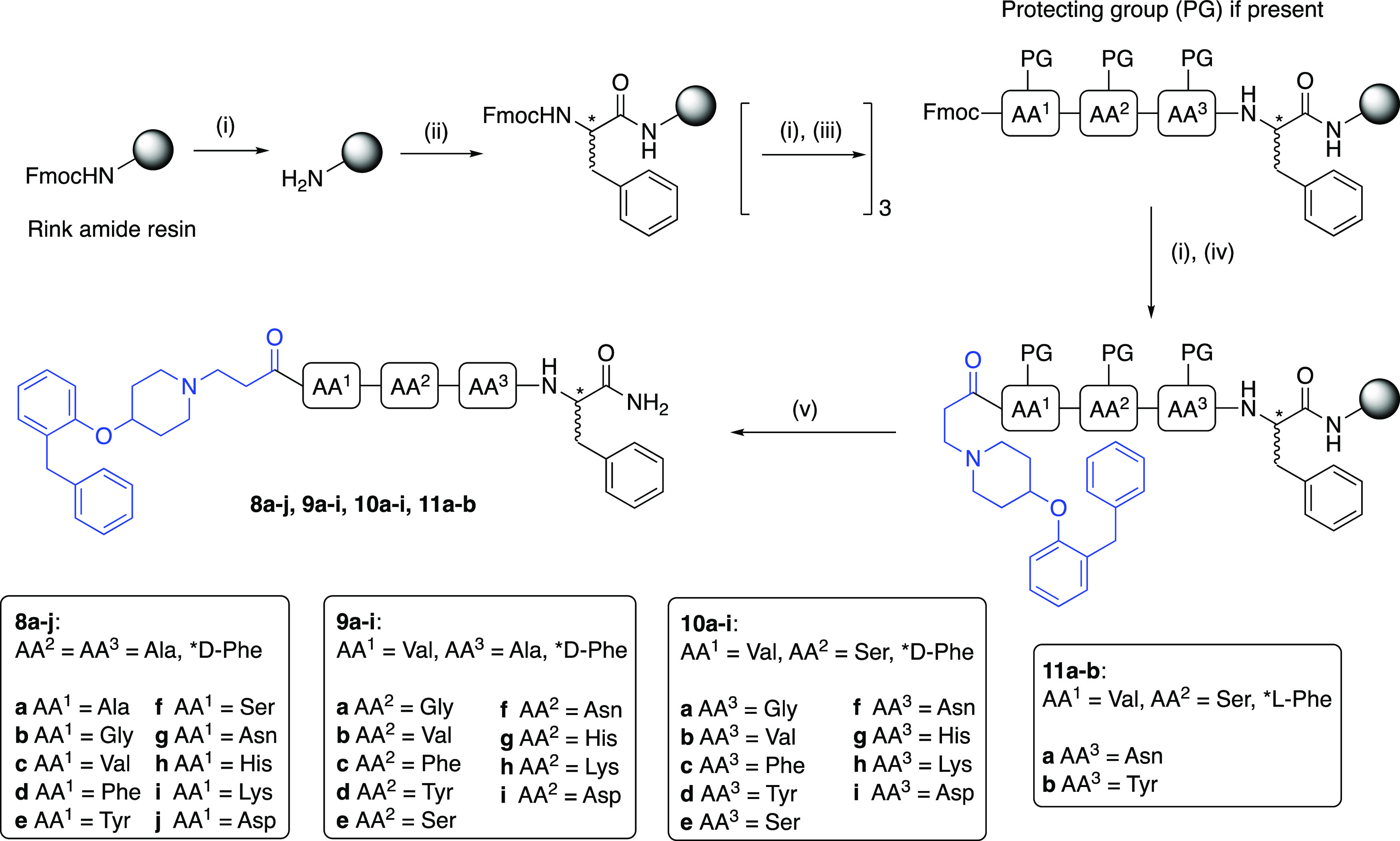
Synthesis of Peptide Congeners (**8a**–**j**, **9a**–**i**, **10a**–**i**, and **11a**,**b**) Reagents and conditions: (i) 20% piperidine/DMF, rt, 5 min; (ii) **8a**–**j**, **9a**–**i**, **10a**–**i**: Fmoc-d-Phe-OH; **11a,b**: Fmoc-Phe-OH; HBTU, 1-hydroxybenzotriazole (HOBt), *N,N*-diisopropylethylamine (DIPEA), *N,N*-dimethylformamide (DMF), rt, 4 h; (iii) Fmoc-amino acid-OH (Fmoc-Gly-OH, Fmoc-Ala-OH, Fmoc-Val-OH, Fmoc-Phe-OH, Fmoc-Tyr(*t*Bu)-OH, Fmoc-Ser(*t*Bu)-OH, Fmoc-Asn(Trt)-OH, Fmoc-His(Trt)-OH, Fmoc-Lys(Boc)-OH, Fmoc-Asp(*t*Bu)-OH), HBTU, HOBt, DIPEA, DMF, rt, 4 h; (iv) **7**, HBTU, HOBt, DIPEA, DMF, rt, 4 h; (v) 90% TFA/H_2_O, rt, 3 h, 1–12%.

During the study, the required *N*-Fmoc-3-azido-d-alanine, which was intended for use in the synthesis of the final fluorescent ligands, ceased to be commercially available; however, the enantiomer *N*-Fmoc-3-azido-alanine had returned to commercial availability. As we had carried out the congener SAR study using a d-Phe surrogate, we deemed it prudent to also synthesize the corresponding l-Phe bearing epimers of the best analogues, to evaluate whether the stereochemistry at this distal point of the linker was likely to play a role in determining affinity. With this in mind, **11a,b** (Scheme 2) were synthesized as the l-Phe epimers of **10d,e** (the highest affinity congeners, Table 1).

**Table 1:**
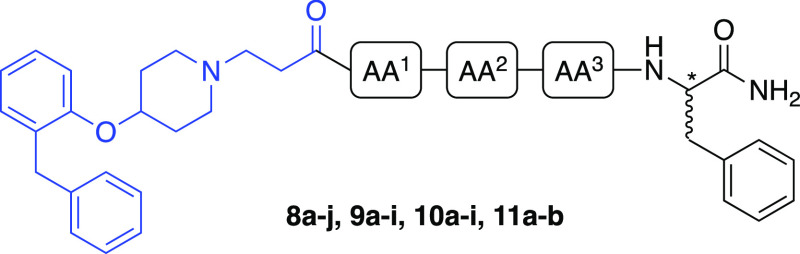
Binding Affinity of Peptide Congeners at Nluc-H_1_R Expressed in HEK293T Cells^a^

	AA^1^	AA^2^	AA^3^	*d/l-Phe	p*K*_i_	*K*_i_ (nM)
**8a**	Ala	Ala	Ala	d-Phe	6.04 ± 0.16	912
**8b**	Gly				6.29 ± 0.21	513
**8c**	Val				6.80 ± 0.12	158
**8d**	Phe				5.88 ± 0.21	1318
**8e**	Tyr				5.93 ± 0.17	1175
**8f**	Ser				6.39 ± 0.15	407
**8g**	Asn				6.25 ± 0.20	562
**8h**	His				6.03 ± 0.13	933
**8i**	Lys				5.79 ± 0.18	1622
**8j**	Asp				5.99 ± 0.24	1023
**9a**	Val	Gly	Ala	d-Phe	6.30 ± 0.07	501
**9b**		Val			6.43 ± 0.06	372
**9c**		Phe			6.10 ± 0.20	794
**9d**		Tyr			6.36 ± 0.04	437
**9e**		Ser			6.82 ± 0.06	151
**9f**		Asn			6.08 ± 0.03	832
**9g**		His			6.16 ± 0.06	692
**9h**		Lys			5.62 ± 0.06	2399
**9i**		Asp			6.26 ± 0.14	550
**10a**	Val	Ser	Gly	d-Phe	6.80 ± 0.05	158
**10b**			Val		6.51 ± 0.07	309
**10c**			Phe		6.96 ± 0.02	110
**10d**			Tyr		7.02 ± 0.08	95
**10e**			Ser		6.47 ± 0.03	339
**10f**			Asn		7.02 ± 0.08	95
**10g**			His		6.90 ± 0.04	126
**10h**			Lys		6.73 ± 0.05	186
**10i**			Asp		6.76 ± 0.04	174
**11a**	Val	Ser	Asn	l-Phe	7.05 ± 0.20	89
**11b**	Val	Ser	Tyr		6.36 ± 0.22	437

a Binding affinity (p*K*_i_/*K*_i_) determined by displacement of **1** (*K*_D_ = 8 nM) from Nluc-H_1_R expressed on HEK293T cells and p*K*_i_/*K*_i_ values obtained using the Cheng–Prusoff equation. The data shown are the means ± SEM from three independent experiments.

#### Synthesis of CuAAC-Coupled H_1_R Fluorescent Ligands

**Scheme 3:**
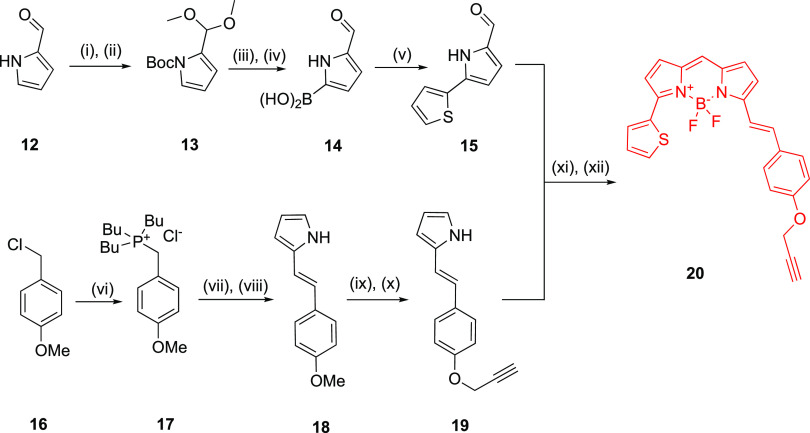
Synthesis of Alkyne-functionalized BODIPY 630/650-Based Fluorophore (**20**) Reactions and conditions: (i) Boc anhydride, triethylamine, DMAP, CH_2_Cl_2_, rt, 30 min; (ii) CH(OCH_3_)_3_, *para*-toluenesulfonic acid, MeOH, rt, 3 h, 87% over two steps; (iii) triisopropylborate, lithium diisopropylamide, THF, 0 °C, N_2_ atm, 1 h; (iv) NH_4_Cl quenching, 10% NaHSO_4_, 70 °C, 2 h, 34 °C, 34% over two steps; (v) 2-bromothiophene, Na_2_CO_3_, Pd(PPh_3_)_2_Cl_2_, 10% H_2_O/1,4-dioxane, 100 °C, N_2_ atm, 3 h, 32%; (vi) P(*n*-Bu)_3_, toluene, reflux, N_2_ atm, 15 h, 85%. (vii) NaOH (aq), H_2_O, rt, sonicate 5 min; (viii) 2-formylpyrrole, 100 °C MW, 30 min, 76%; (ix) NaSEt, DMF, reflux, N_2_ atm, 15 h; (x) propargyl bromide, K_2_CO_3_, acetonitrile, reflux, 15 h, 88% over two steps; (xi) POCl_3_, CH_2_Cl_2_, rt, N_2_ atm, 15 h; (xii) BF_3_·Et_2_O, DIPEA, CH_2_Cl_2_, rt, 2 h, 69% over two steps.

We proceeded with synthesis of fluorescent ligands using CuAAC, incorporating the optimized peptide linker identified from the above congener screening study. To achieve this, we synthesized a novel, alkyne-functionalized fluorophore based on BODIPY 630/650 (**20**) via a twelve-step synthesis route as outlined in Scheme 3. 2-Formylpyrrole (**12**) was sequentially reacted with Boc-anhydride and trimethylorthoformate to protect the pyrrole N–H and aldehyde groups, respectively, to give **13**. Boronation at C5 via triisopropyl borate in the presence of lithium diisopropylamide and subsequent acid-mediated deprotection using sodium hydrogen sulfate yielded the boronic acid product **14**, which was then subjected to a Suzuki reaction with 2-bromothiophene to afford **15**.

Tributylphosphine was added to *para*-methoxybenzyl chloride (**16**) and the mixture subsequently stirred in anhydrous toluene to give the phosphonium salt (**17**), which was then treated with NaOH and subjected to a Wittig reaction with 2-formylpyrrole at 100 °C under microwave conditions to afford **18**. Demethylation of the methoxy group was achieved in the presence of NaSEt to yield the corresponding phenol, which was subsequently alkylated with propargyl bromide, affording **19**. Compound **15** and **19** were then stirred in anhydrous dichloromethane (DCM) in the presence of POCl_3_ overnight and subsequently treated with an excess of DIPEA and BF_3_·Et_2_O to afford the BODIPY fluorophore **20** as a red iridescent solid.

**Scheme 4:**
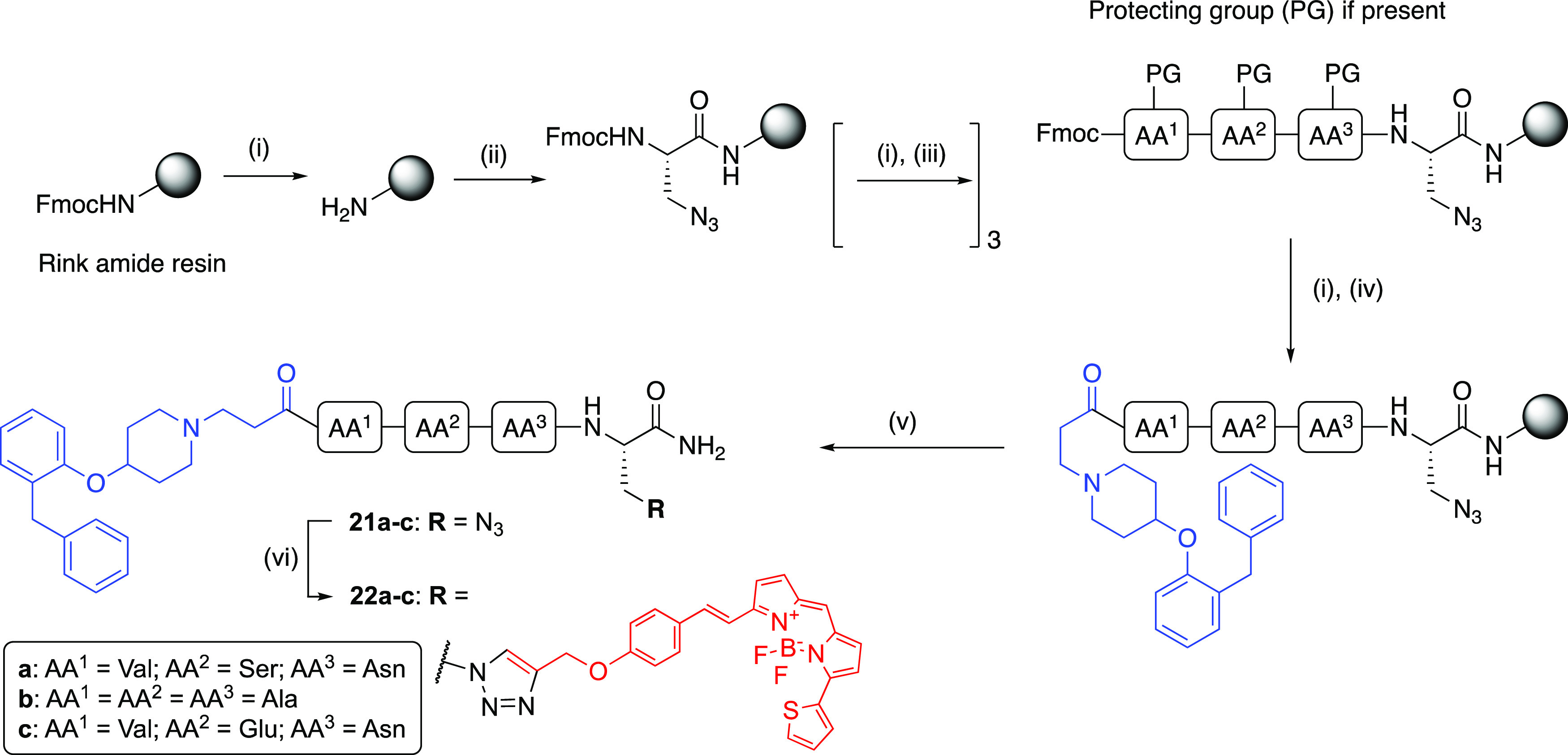
Synthesis of Fluorescent Ligands (**22a**–**c**) via CuAAC Reactions and conditions: (i) 20% piperidine/DMF, rt, 5 min. (ii) Fmoc-β-azido-Ala-OH, HBTU, HOBt, DIPEA, DMF, rt, 4 h; (iii) Fmoc-amino acid-OH (Fmoc-Val-OH, Fmoc-Ser(*t*Bu)-OH, Fmoc-Asn(Trt)-OH, Fmoc-Ala-OH, Fmoc-Glu(*t*Bu)-OH), HBTU, HOBt, DIPEA, DMF, rt, 4 h; (iv) **7**, HBTU, HOBt, DIPEA, DMF, rt, 4 h; (v) 90% TFA/H_2_O, rt, 3 hr, 2-3%; (vi) **20**, CuSO_4_, sodium ascorbate, 2,6-lutidine, DMSO, rt, 18 h, 60–79%.

As described earlier, inspection of the crystal structure of doxepin bound to the human H_1_R (PDB code: 3RZE) reveals basic residues (K179^ECL2^, K191^5.39^, and H450^7.35^) lining the entrance to the orthosteric binding site. Preliminary molecular docking studies (Supporting informationFigure S1) suggested that incorporating an acidic amino acid residue at AA^2^ might permit salt bridge formation with K179^ECL2^ and K191^5.39^; however, improvement in affinity was not achieved with **9i** (AA^1^ = Val, AA^2^ = Asp, AA^3^ = Ala) in our congener screen (Table 1). We decided to pursue this hypothesis further by incorporating glutamate at AA^2^ to probe for the desired salt bridge interaction, which was potentially achievable given the flexibility associated with the additional methylene spacer within the glutamate side group compared to the aspartate side group. Three analogues (**21a**–**c**) bearing either the Val-Ser-Asn (**21a**), linker which was determined to be the optimum peptide linker from the congener screen, Ala-Ala-Ala (**21b**) linker as a control, or Val-Glu-Asn (**21c**) linker were synthesized using SPPS and conjugated to **20** using CuAAC to afford their corresponding fluorescent ligands (**22a-c**) (Scheme 4).

**Scheme 5:**
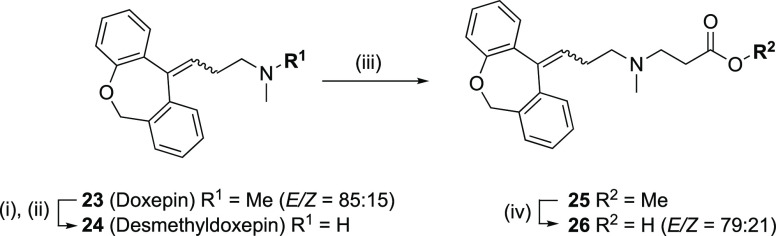
Synthesis of *N*-(2-Carboxyethyl)nordoxepin (**26**) Reactions and conditions: (i) trichloroethyl chloroformate, CH_2_Cl_2_, triethylamine, rt, N_2_ atm, 6 h; (ii) Zn powder, 1 M NaH_2_PO_4_, THF, rt, N_2_ atm, 15 h, 45% over two steps; (iii) methyl acrylate, DIPEA, 1,2-dichloroethane, 75 °C, 15 h, 69%; (iv) NaOH, H_2_O/THF, rt, 1 h, 96%.

The three main components of a fluorescent ligand, i.e., orthostere, linker, and fluorophore, may exert an effect on fluorescent ligand binding affinity.^47^ To this end, we conducted a series of investigations to understand the effects of each of these components on the binding affinity of our H_1_R fluorescent ligands. We hypothesized that doxepin, which possessed subnanomolar affinity for H_1_R (radioligand binding assay p*K*_i_ = 9.75),^48^ when incorporated into fluorescent ligands as the orthostere could improve overall ligand affinity at H_1_R relative to VUF13816 (radioligand binding assay p*K*_i_ = 8.20).^48^ This was achieved by incorporating the *N*-(2-carboxyethyl) analogue of desmethyldoxepin (**26**) instead of the VUF13816-based **7** as the orthostere within the congeners. Compound **26** was derived from doxepin hydrochloride (**23,** manufacturer reported *E*:*Z* ratio = 85:15) (Scheme 5). Doxepin hydrochloride (**23**) was treated with trichloroethyl chloroformate, and subsequent reductive cleavage of the trichloroethoxycarbonyl group using zinc powder in the presence of 1 M NaH_2_PO_4_ afforded nordoxepin (**24**). This was then subjected to Michael addition with methyl acrylate in the presence of DIPEA and the resulting methyl ester product (**25**) was hydrolyzed in excess NaOH with subsequent acidification affording the free acid (**26)** with *E*:*Z* ratio = 79:21 determined by ^1^H NMR.

**Scheme 6:**
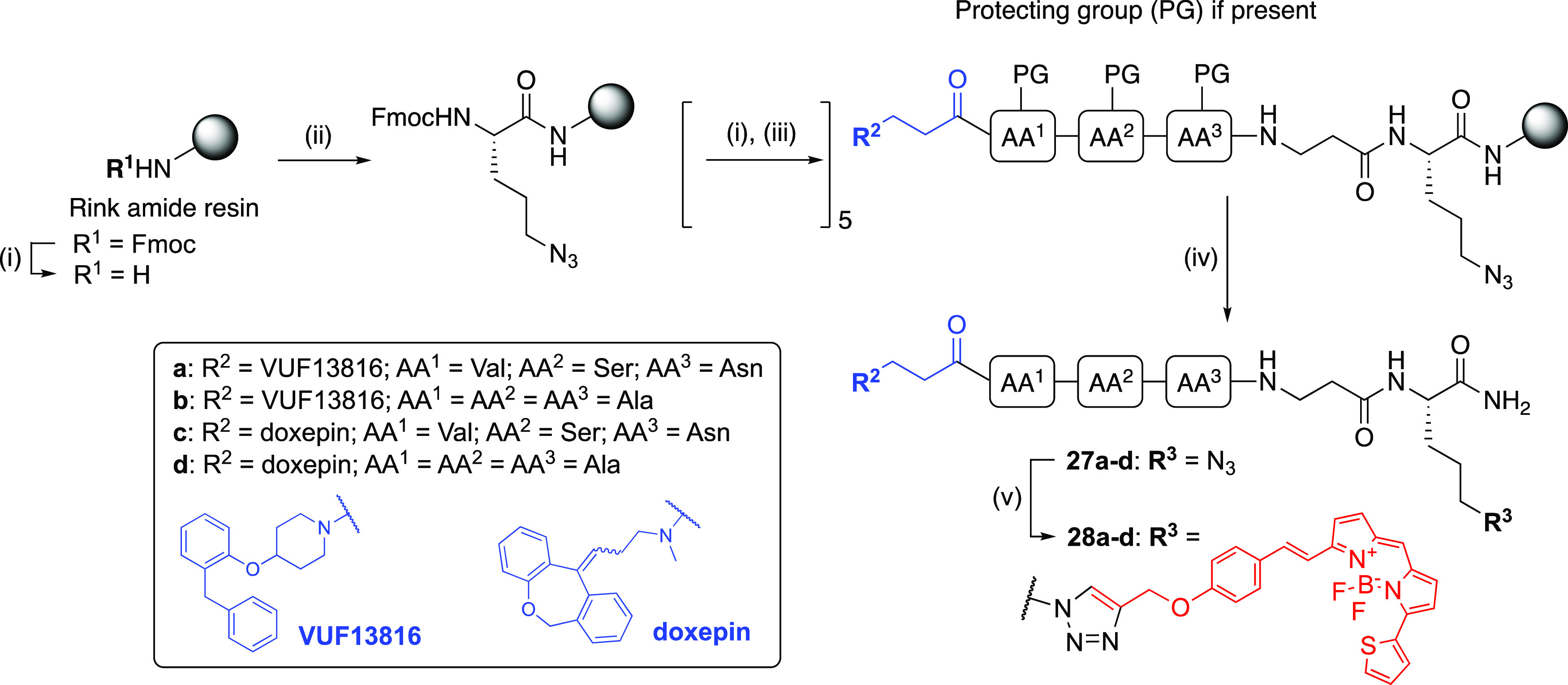
Synthesis of Fluorescent Ligands (**28a**–**d**) via CuAAC Reactions and conditions: (i) 20% piperidine/DMF, rt, 5 min. (ii) Fmoc-Orn(N_3_)-OH, HBTU, HOBt, DIPEA, DMF, rt, 4 h; (iii) **7** or **26** or Fmoc-amino acid-OH (Fmoc-Val-OH, Fmoc-Ser(*t*Bu)-OH, Fmoc-Asn(Trt)-OH, Fmoc-Ala-OH), HBTU, HOBt, DIPEA, DMF, rt, 4 h; (iv) 90% TFA/H_2_O, rt, 3 h, 5-11%; (v) **20**, CuSO_4_, sodium ascorbate, 2,6-lutidine, DMSO, rt, 18 h, 33–79%.

The initial series of fluorescent ligands (**22a**–**c**) were considerably shorter in overall length in comparison to our previously reported probe (**1**). As such, we sought to synthesize analogues, which were more comparable in size to **1**, through addition of a spacer between AA^3^ and the triazole link to the fluorophore. Congeners (**27a**–**d**) were synthesized using SPPS on Rink amide resin, initiated by resin loading with Fmoc-5-azido-l-norvaline-OH (Fmoc-Orn(N_3_)-OH) and the subsequent incorporation of β-alanine, the required tripeptide, and final capping with the orthostere. The propargyl-bearing fluorophore (**20**) was conjugated to congeners **27a**–**d** by CuAAC in the presence of 2,6-lutidine and a Cu(I) catalyst generated in situ through the reduction of Cu(II) by sodium ascorbate, to afford four fluorescent ligands (**28a**–**d**) with a linker length similar to that of **1** (Scheme 6). Compounds **28c,d** were pharmacologically characterized as a mixture of *E/Z* isomers (79:21), arising from the doxepin-based orthostere (**26**).

#### Synthesis of Amide-Coupled H_1_R Fluorescent Ligands

**Scheme 7:**
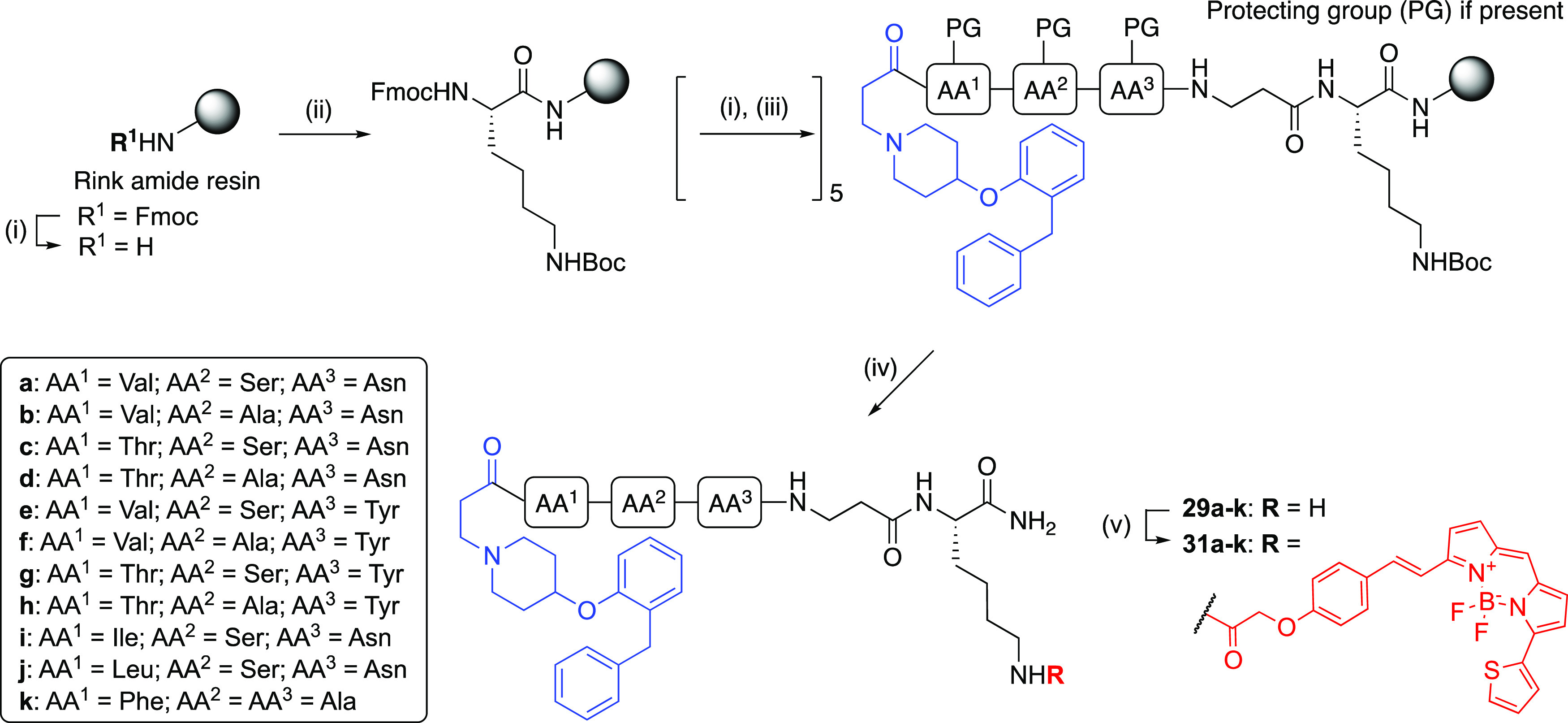
Synthesis of Fluorescent Ligands (**31a**–**k**) via Amide Coupling Reactions and conditions: (i) 20% piperidine/DMF, rt, 5 min. (ii) Fmoc-Lys(Boc)-OH, HBTU, HOBt, DIPEA, DMF, rt, 4 h; (iii) Fmoc-amino acid-OH (Fmoc-Val-OH, Fmoc-Ser(*t*Bu)-OH, Fmoc-Asn(Trt)-OH), Fmoc-Ala-OH, Fmoc-Thr(*t*Bu)-OH, Fmoc-Tyr(*t*Bu)-OH, Fmoc-Ile-OH, Fmoc-Leu-OH, Fmoc-Phe-OH) or **7**, HBTU, HOBt, DIPEA, DMF, rt, 4 h; (iv) 90% TFA/H_2_O, rt, 3 h, 4–8%; (v) **30**, HATU, DIPEA, DMF, rt, 2 h, 27–45%.

Next, we compared our triazole-linked approach to fluorophore installation with a selection of corresponding amide-linked analogues (Scheme 7). The peptide linker selection was based upon the amino acids previously observed to provide the greatest improvement in congener affinity, namely, AA^1^ = Val, AA^2^ = Ser or Ala, and AA^3^ = Asn or Tyr. We opted to explore these in all possible combinations as well as AA^1^ = Phe, AA^2^ = Ala, and AA^3^ = Ala (corresponding to **8d**) as a control. As significant improvement in congener affinity was only observed with the linker optimization at AA,^1^ we sought to explore the SAR at AA^1^ in further detail, through the incorporation of a hydrophilic Thr and the sterically bulkier Ile and Leu, which had not been previously explored at this position. The congeners (**29a**–**k**) were assembled on Rink amide resin via SPPS, starting with Fmoc-Lys(Boc)-OH for resin loading (*N*ε-Boc-protection masking the fluorophore attachment point), followed by β-alanine, the required tripeptide, and capping with **7**. Compounds **29a**–**k** were conjugated to a carboxylic acid-functionalized BODIPY 630/650-based fluorophore^49^ (**30**) in the presence of the coupling reagent 1-[bis-(dimethylamino)methylene]-1*H*-1,2,3-triazolo[4,5-*b*]pyridinium-3-oxide hexafluorophosphate (HATU) and DIPEA to afford fluorescent ligands **31a**–**k**.

**Scheme 8:**
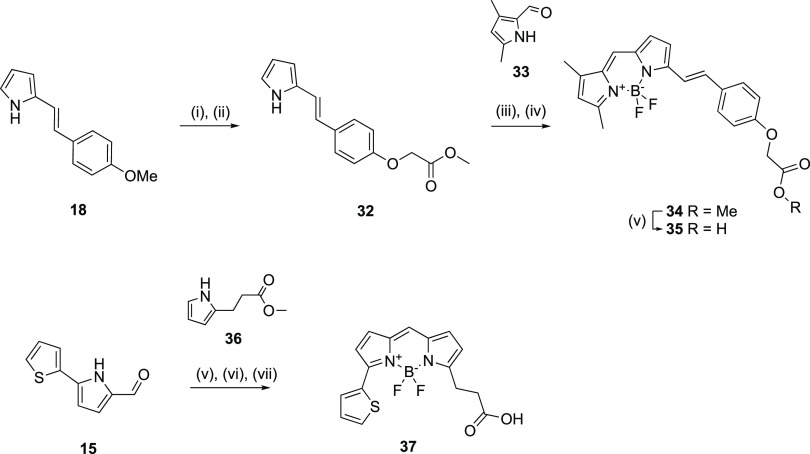
Synthesis of BODIPY A (35) and BODIPY B (**37**) Reactions and conditions: (i) NaSEt, DMF, reflux, N_2_, 15 h. (ii) methyl bromoacetate, K_2_CO_3_, MeCN, reflux, 15 h, 69%. (iii) **33**, POCl_3_, CH_2_Cl_2_, rt, 15 h. (iv) BF_3_·Et_2_O, DIPEA, CH_2_Cl_2_, rt, 2 h, 19%. (v) 85% H_3_PO_4_, 2:1 THF/H_2_O, 65 °C, N_2_ atm, 90 hr, 92%. (vi) **36**, POCl_3_, CH_2_Cl_2_, RT, 15 hr. (vii) BF_3_·Et_2_O, DIPEA, CH_2_Cl_2_, RT, N_2_ atm, 2 h, 17%. (viii) 85% H_3_PO_4_, 2:1 THF/H_2_O, 65 °C, N_2_ atm, 90 h, 63%.

**Scheme 9:**
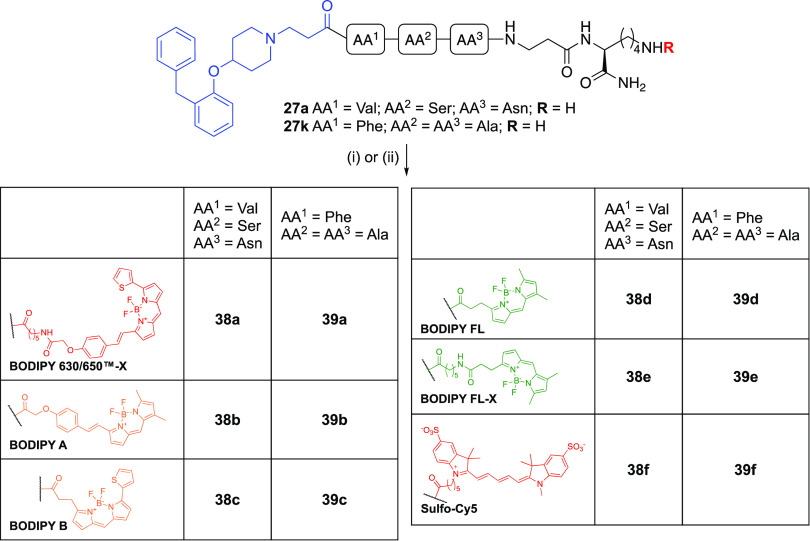
Synthesis of Fluorescent Ligands (**38a**–**f** and **39a**–**f**) via Amide Coupling Reactions and conditions: (i) **38a, 38d-f**, **39a**, and **39d-f**: Fluorophore-NHS ester, DMF, rt, 2 h; (ii) **38b,c** and **39b,c**: **35** or **37**, HATU, DIPEA, DMF, RT, 2 h, 41–82%

Our final area of SAR exploration was to investigate how the structure of fluorophore and linker moieties affected overall ligand binding affinity at the H_1_R by conjugating **29a** bearing the optimized linker (AA^1^ = Val, AA^2^ = Ser, AA^3^ = Asn) and **29k** bearing an unoptimized linker (AA^1^ = Phe, AA^2^ = AA^3^ = Ala) as controls, to six different fluorophores. We sought to explore the SAR relating to the BODIPY scaffold and also to compare lipophilic and hydrophilic fluorophores with similar absorption/emission profiles. The panel of commercially available *N*-reactive (as the *N-*hydroxysuccinimide (NHS) ester) fluorophores included those incorporating a flexible hexanoyl linker: BODIPY FL-X (green-emitting, lipophilic), BODIPY 630/650-X (red-emitting, lipophilic), and sulfo-Cyanine 5 (sulfo-Cy5, water soluble, red-emitting) and the corresponding BODIPY-FL (green-emitting, lipophilic), which lacks the hexanoyl linker. In order to compare the structures of BODIPY 630/650 and BODIPY-FL more effectively, we additionally designed and synthesized hybridized versions of these fluorophores: BODIPY A (**35)** and BODIPY B (**37**) from **32** and **15,** respectively (Schemes 8 and 9).

Compound **18** was subjected to demethylation with NaSEt before treating it with K_2_CO_3_ and alkylation with methyl bromoacetate to give the ester **32**. Condensation of **32** and 3,5-dimethyl-1*H*-pyrrole-2-carbaldehyde (**33**) in the presence of POCl_3_ and subsequent treatment with BF_3_·Et_2_O afforded the BODIPY ester **34**, which upon acid hydrolysis in the presence of H_3_PO_4_ afforded the free acid BODIPY A (**35)**.

BODIPY B (**37**) was synthesized in a similar manner with the condensation of **15** and methyl 3-(1*H*-pyrrol-2-yl)propanoate (**36**, followed by mild acidic hydrolysis of the resulting ester in the presence of H_3_PO_4_ to afford the free acid **37**. Finally, **29a** and **29k** were individually coupled to each fluorophore to afford twelve fluorescent ligands **38a**–**f** and **39a**–**f** (Scheme 9). The BODIPY A-based (**38b**, **39b**) and BODIPY B-based (**38c**, **39c**) fluorescent ligands were spectrally characterized on a FlexStation3 plate reader (Molecular Devices, Sunnyvale, CA) and their absorption/emission (λ_max_,_ex_/λ_max_,_em_) spectral profiles were determined to be 570 nm/586 nm and 560 nm/570 nm, respectively (Figures S2–S5). A figurative summary of all synthesized fluorescent compounds is included in the Supporting Information (Figure S6).

### Pharmacology

#### Binding Affinity Evaluation of Peptide Congeners (**8a**–**j, 9a**–**i, 10a**–**i, 11a**,**b**)

It has been previously reported that NanoBRET-based binding assays performed on Nanoluciferase-tagged human H_1_R (Nluc-H_1_R)-expressing HEK293T cells produced binding affinity values (p*K*_i_) comparable to those obtained from radioligand binding assays as well as p*K*_B_ values obtained from Ca^2+^ mobilization assays.^25^ As such, NanoBRET-based assays performed on Nluc-H_1_R-expressing HEK293T cells were selected as the primary assay for the pharmacological characterization of the unlabeled peptide congeners and the final fluorescent conjugates in this study. NanoBRET assays have several advantages over traditional radioligand binding assays including ease of performing the assay and the lack of safety issues associated with disposal of radioactive material.^13−17^

**Figure 3:**
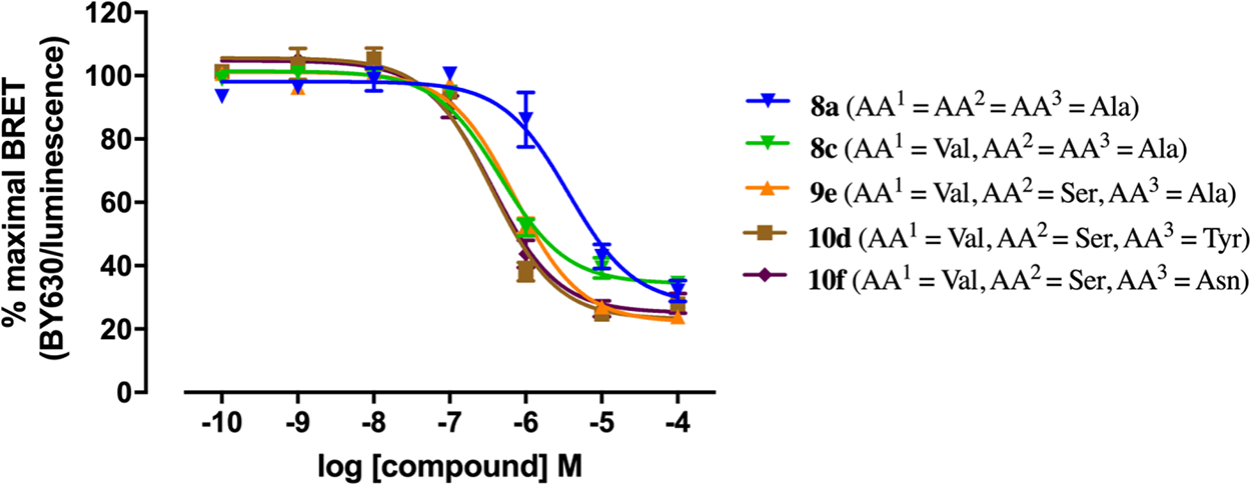
Baseline-corrected displacement curves for **8a**, **8c**, **9e**, **10d**, and **10f** determined by a NanoBRET competition assay on live Nluc-H_1_R expressing HEK293T cells treated with 25 nM **1** as the fluorophore-labeled competitive ligand with increasing concentrations of the unlabeled congeners. A gradual leftward shift of the displacement curve relative to that of **8a** was observed upon linker optimization at AA^1^ (**8c)**, AA^2^ (**9e**), and AA^3^**(10d** and **10f**). Linker optimization at AA^1^ (**8a** to **8c**) provided the greatest increase in affinity, whereas linker optimization at AA^2^ (**8c** to **9e**) and AA^3^ (**9e** to **10d** and **10f**) did not significantly improve affinity. Data were normalized to maximal BRET signal obtained in the absence of labeled competitive ligand (**1**) and data shown represent the combined mean ± SEM of three experiments performed in triplicate.

The binding affinity of **8a**–**j**, **9a**–**i,** and **10a**–**i** at the H_1_R was determined by competition with **1** at Nluc-H_1_R expressed on HEK293T cells in a whole cell NanoBRET equilibrium binding assay (Table 1). The affinities (*K*_i_) ranged from 95 nM (**10d/10f**) to 2399 nM (**9h**), indicating that the composition of the peptide linker does affect affinity. The analogues with the highest affinity **10d** (AA^1^ = Val, AA^2^ = Ser, AA^3^ = Tyr; *K*_i_ = 95 nM) and **10f** (AA^1^ = Val, AA^2^ = Ser, AA^3^ = Asn; *K*_i_ = 95 nM) conferred a 10-fold improvement in affinity compared to **8a** (AA^1^ = AA^2^ = AA^3^ = Ala; *K*_i_ = 912 nM), which served as the starting point for our optimization effort. These observations provided confidence that the synthesis of a fluorescent ligand with a higher binding affinity at H_1_R compared to **1** could be achieved through peptide linker optimization. Although we observed a stepwise, gradual improvement in affinity progressing along the three series, the greatest improvement in affinity from the linker optimization effort was attributed to the incorporation of Val at AA^1^ (**8c**, AA^1^ = Val, AA^2^ = AA^3^ = Ala; *K*_i_ = 158 nM), which provided a 6-fold improvement in affinity over **8a**. Optimization efforts at AA^2^ (from **8c** to **9e**) and AA^3^ (from **9e** to **10d** and **10f**) did not significantly improve affinity (less than 2-fold) as indicated by the lack of significant leftward shifts in the position of displacement curves (Figure 3). In addition, we observed that the configuration of the terminal phenylalanine in our set of congeners had a context-dependent effect on overall affinity. In the case of **11b** (AA^1^ = Val, AA^2^ = Ser, AA^3^ = Tyr; *K*_i_ = 437 nM), H_1_R affinity was 5-fold lower than that of the d-Phe epimer **10d** (*K*_i_ = 95 nM). In contrast, **11a** (AA^1^ = Val, AA^2^ = Ser, AA^3^ = Asn; *K*_i_ = 89 nM) and its epimer **10f** (*K*_i_ = 95 nM) had comparable affinity. The lower affinity of **11b** compared to its epimer, **10d** may be explained by unfavorable intramolecular interactions that might occur in one epimer due to the adjacent aromatic groups present in AA^3^ (Tyr) and Phe. Given the later incorporation of a lipophilic fluorophore, we opted to proceed with only the Val-Ser-Asn linker being incorporated into CuAAC-conjugated H_1_R fluorescent ligands. The presence of Asn at AA^3^ rather than Tyr confers improved overall physicochemical properties to the molecule in the context of the significant lipophilicity ultimately added by the fluorophore.

#### Binding Affinity Evaluation of CuAAC-Coupled Fluorescent Ligands (**22a**–**c, 28a**–**d**)

The binding affinity of fluorescent ligands **22a**–**c** and **28a**–**d** was determined by a NanoBRET saturation binding assay performed on Nluc-H_1_R expressed in HEK293T cells, whereby all seven fluorescent ligands displayed concentration-dependent, saturable and reversible binding to Nluc-H_1_R (Figures S7 and S8). It was interesting to observe that the SAR determined in the linker optimization study did not translate through in the context of the full CuAAC-coupled fluorescent ligand as compounds **22a**, **28a,** and **28c** (all with AA^1^ = Val, AA^2^ = Ser, AA^3^ = Asn) did not exhibit a significant improvement in binding affinity compared to their corresponding control compounds **22b**, **28b,** and **28d,** respectively (AA^1^ = AA^2^ = AA^3^ = Ala) (Table 2). Moreover, doxepin-based fluorescent ligand **28d** (AA^1^ = AA^2^ = AA^3^ = Ala; *K*_D_ = 5 nM) exhibited an affinity that was 4-fold higher than **28c** (AA^1^ = Val, AA^2^ = Ser, AA^3^ = Asn; *K*_D_ = 19 nM) at the H_1_R.

**Table 2:**
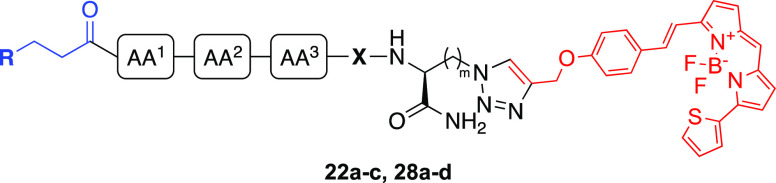

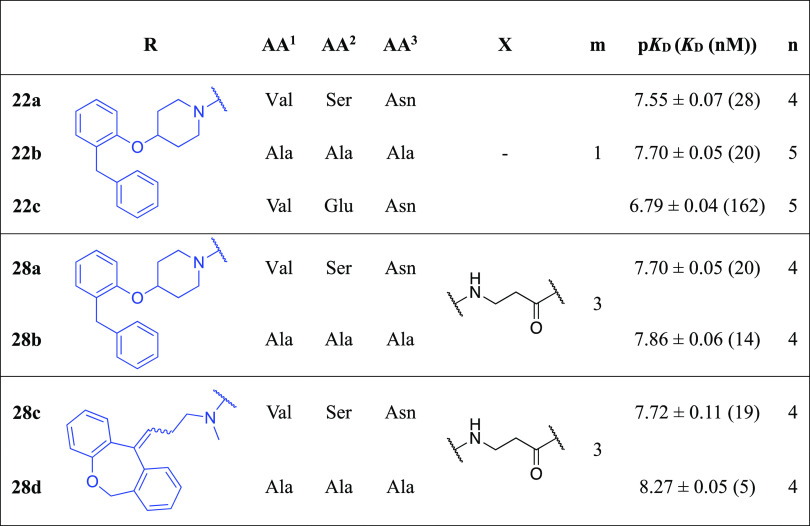
Binding Affinity of 22a-c and 28a-d at Nluc-H_1_R Expressed in HEK293T Cells^*a*^

a Binding affinity (p*K*_D_/*K*_D_) determined from saturation binding curves in NanoBRET saturation binding assay conducted in Nluc-H_1_R expressing HEK293T cells. The data shown are the means ± SEM from *n* separate experiments.

When comparing ligands with a short spacer between the tripeptide sequence and the fluorophore (**22a** and **22b**) with ligands bearing the same tripeptide sequence, but a longer spacer (**28a** and **28b** respectively), the difference in spacer length had no discernible effect on overall ligand affinity. Similarly, comparing ligands with a constant tripeptide linker and spacer length, but varying the orthostere from VUF13816 (**28a-b**) to doxepin (**28c-d**), had no significant effect on overall H_1_R affinity.

This indicates that the fluorescent ligands, which differ from their corresponding unlabeled peptide congeners in terms of their overall structure, size, and physicochemical properties, can present divergent SAR from the congener screen. This is in agreement to previous work by ourselves and others, whereby fluorescent ligands or their congeners must be treated as new pharmacological entities in their own rights rather than simple extensions of the original orthostere.^13,42,47,50−53^

Our efforts to engage the three basic residues at the entrance of the binding pocket (K179^45.49^, K191^5.39^, and H450^7.35^) via salt bridge interactions using acidic side-chain bearing peptide linkers did not confer the anticipated improvement to congener affinity as seen in the case of **8j** (AA^1^ = Asp; AA^2^ = AA^3^ = Ala; K_i_ = 1023 nM), **9i** (AA^1^ = Val, AA^2^ = Asp, AA^3^ = Ala; K_i_ = 550 nM), and **10i** (AA^1^ = Val, AA^2^ = Ser, AA^3^ = Asp; K_i_ = 174 nM). This was also mirrored in the fluorescent ligands, as switching AA^2^ from the polar, neutral serine in **22a** (AA^1^ = Val, AA^2^ = Ser, AA^3^ = Asn; *K*_D_ = 28 nM) to the acidic glutamate in **22c** (AA^1^ = Val, AA^2^ = Glu, AA^3^ = Asn; *K*_D_ = 162 nM) led to a 6-fold loss in affinity. We therefore concluded that it was not possible to improve fluorescent ligand affinity through the acidic linker approach.

Although there were initial concerns regarding the CuAAC-derived triazole interfering with fluorescent ligand binding, our results have demonstrated that CuAAC is a feasible approach towards fluorophore conjugation with the synthesis of high affinity H_1_R fluorescent ligands (*K*_D_ values ranging from 6 to 31 nM). Therefore, CuAAC should be considered as an alternative fluorophore conjugation strategy in the synthesis of fluorescent ligands, especially where the orthostere or linker moieties bear nucleophilic moieties liable to partake in undesirable side reactions when using a classical N-reactive fluorophore approach to generate an amide bond linkage. Furthermore, while protective group strategies could be considered in this situation, their removal following fluorophore conjugation is not always feasible without detriment to fluorophore stability.

#### Binding Affinity Evaluation of Amide-Coupled Fluorescent Ligands (**31a**–**k, 38a**–**f, 39a**–**f**)

The binding affinity of **31a**–**k**, **38a**–**f,** and **39a**–**f** was determined by a NanoBRET saturation binding assay performed on Nluc-H_1_R expressed in HEK293T cells, whereby all twenty-three fluorescent ligands displayed concentration-dependent, saturable and reversible binding to Nluc-H_1_R (Figures S9-S12). For **31a**–**k,***K*_D_ values ranged from 4–16 nM, representing a 4-fold difference overall (Table 3). Compound **31a** (AA^1^ = Val, AA^2^ = Ser, AA^3^ = Asn; *K*_D_ = 4 nM) showed a 3-fold improvement in binding affinity over **31k** (AA^1^ = Phe, AA^2^ = AA^3^ = Ala; *K*_D_ = 12 nM), considerably lower than the 14-fold increase in binding affinity observed between the corresponding congeners **10f** (AA^1^ = Val, AA^2^ = Ser, AA^3^ = Asn; *K*_i_ = 95 nM) and **8d** (AA^1^ = Phe, AA^2^ = AA^3^ = Ala; *K*_i_ = 1318 nM). This provides further evidence that the addition of a fluorophore to the congener seems to override the SAR observed in the context of the congeners alone.

**Table 3:**
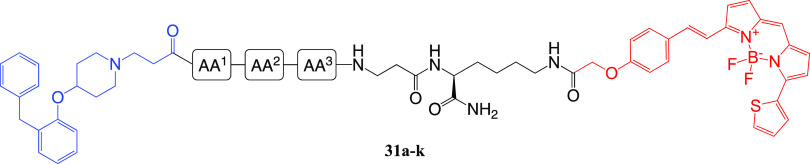
Binding Affinity of **31a**–**k** at Nluc-H_1_R Expressed in HEK293T Cells^a^

	AA^1^	AA^2^	AA^3^	p*K*_D_	*K*_D_ (nM)
**31a**	Val	Ser	Asn	8.40 ± 0.08	4
**31b**	Val	Ala	Asn	8.34 ± 0.09	5
**31c**	Thr	Ser	Asn	8.20 ± 0.07	6
**31d**	Thr	Ala	Asn	8.29 ± 0.07	5
**31e**	Val	Ser	Tyr	7.80 ± 0.11	16
**31f**	Val	Ala	Tyr	7.96 ± 0.11	11
**31g**	Thr	Ser	Tyr	8.16 ± 0.12	7
**31h**	Thr	Ala	Tyr	8.23 ± 0.17	6
**31i**	Ile	Ser	Asn	8.33 ± 0.09	5
**31j**	Leu	Ser	Asn	8.23 ± 0.06	6
**31k**	Phe	Ala	Ala	7.92 ± 0.08	12

a Binding affinity (p*K*_D_/*K*_D_) determined from saturation binding curves in A NanoBRET saturation binding assay conducted in Nluc-H_1_R expressing HEK293T cells. The data shown are the means ± SEM from four separate experiments.

Previous work on fluorescent ligands has shown that the linker and the fluorophore could affect the pharmacology of the final fluorescent conjugate.^47^ In this study, we observed that the affinities of our peptide congeners series (p*K*_i_ = 5.62–7.05) were at least 10-fold lower when compared to **VUF14454** (radioligand binding assay p*K*_i_ at H_1_R = 8.2),^54^ an *N*-methylated VUF13816 analogue, which retains a tertiary amine structure as seen in our VUF13816-based orthostere. This suggests that linker incorporation has a detrimental effect to overall ligand affinity, in contrast to our initial intention to improve ligand–receptor interaction and thus affinity via an optimized linker. Indeed, affinity could not be improved solely based on ligand-receptor interaction without the consideration of potentially unfavorable ligand binding orientation within the receptor and/or desolvation energy, which might be the case for our peptide congeners. In addition, we also observed a large increase in the binding affinity of fluorescent ligands compared to their corresponding congeners. For example, compound **31a** (AA^1^ = Val, AA^2^ = Ser, AA^3^ = Asn; *K*_D_ = 4 nM) and **31k** (AA^1^ = Phe, AA^2^ = AA^3^ = Ala; *K*_D_ = 12 nM) had significantly higher binding affinities at H_1_R compared to their corresponding congeners **10f** (AA^1^ = Val, AA^2^ = Ser, AA^3^ = Asn; *K*_i_ = 95 nM) and **8d** (AA^1^ = Phe, AA^2^ = AA^3^ = Ala; *K*_i_ = 1318 nM), respectively, with a difference of more than 100-fold seen between **31k** and **8d**. This observation suggests the fluorophore itself has a key role to play in determining overall ligand binding affinity and is not merely a passive bystander moiety in this regard. In addition, in our previous development of BODIPY 630/650-based fluorescent ligands at a range of receptors, we have observed that this particular fluorophore appears to confer a high overall ligand binding affinity.^42,47,52,55^

A clear trend was observed for the binding affinity of fluorescent ligands **38a**–**f** and **39a**–**f** determined through a NanoBRET binding assay (Table 4). Interestingly, fluorescent ligand binding affinity decreased gradually in varying magnitude with decreasing fluorophore lipophilicity (cLogP, Table 4) from BODIPY 630/650-X to Sulfo-Cy5 and this was consistent across **38a**–**f** (AA^1^ = Val, AA^2^ = Ser, AA^3^ = Asn) and **39a**–**f** (AA^1^ = Phe, AA^2^ = AA^3^ = Ala). Once again in the context of the whole fluorescent ligand, where each fluorophore was kept the same, the congener composition had little effect on overall affinity (i.e., **31a** compared to **31k** and each of **38a**–**f** compared to **39a**–**f**). The fluorescent ligands incorporating the additional hexanoyl linker, namely, BODIPY 630/650-X-containing **38a** (*K*_D_ = 18 nM) and **39a** (*K*_D_ = 10 nM) and BODIPY FL-X-containing **38e** (*K*_D_ = 5248 nM) and **39e** (*K*_D_ = 2692 nM) broadly retained comparable binding affinity to their shorter counterparts without the hexanoyl linker **31a** (*K*_D_ = 4 nM), **31k** (*K*_D_ = 12 nM), **38d** (*K*_D_ = 1288 nM), and **39d** (*K*_D_ = 1445 nM) respectively. However, the relatively small difference between the shorter and longer ligands was more pronounced where AA^1^ = Val, AA^2^ = Ser, and AA^3^ = Asn.

**Table 4:**
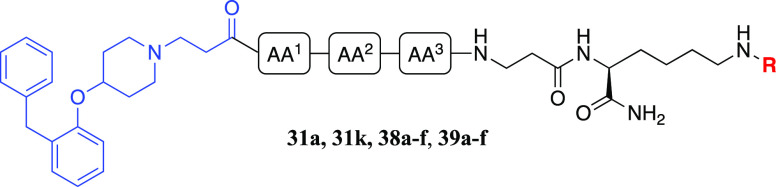
Binding Affinity of **31a**, **31k**, **38a**–**f**, and **39a**–**f** at Nluc-H_1_R Expressed in HEK293T Cells^*a*^ and Human H_1_R Expressed in CHO Cells^b^

	**AA^1^ = Val; AA^2^ = Ser; AA^3^ = Asn**			**AA^1^ = Phe; AA^2^ = AA^3^ = Ala**		
**R** (cLogP of Fluorophore)^c^		p*K*_D_ (*K*_D_ (nM))^a^	p*K*_B_ (*K*_B_ (nM))^b^		p*K*_D_ (*K*_D_ (nM))^a^	p*K*_B_ (*K*_B_ (nM))^b^
BODIPY 630/650	**31a**	8.40 ± 0.08 (4)	8.56 ± 0.13 (3)	**31k**	7.92 ± 0.08 (12)	8.59 ± 0.13 (3)
(5.75)						
BODIPY 630/650^TM^-X	**38a**	7.74 ± 0.06 (18)	-	**39a**	7.98 ± 0.01 (10)	-
(5.80)						
BODIPY A	**38b**	7.18 ± 0.11 (66)	6.96 ± 0.11 (110)	**39b**	7.36 ± 0.03 (44)	6.99 ± 0.10 (102)
(4.62)						
BODIPY B	**38c**	6.68 ± 0.10 (209)	6.52 ± 0.18 (302)	**39c**	6.14 ± 0.06 (724)	6.44 ± 0.14 (363)
(2.76)						
BODIPY FL	**38d**	5.89 ± 0.10 (1288)	6.95 ± 0.12 (112)	**39d**	5.84 ± 0.13 (1445)	7.46 ± 0.07 (35)
(2.67)						
BODIPY FL-X	**38e**	5.28 ± 0.06 (5248)	-	**39e**	5.57 ± 0.05 (2692)	-
(2.65)						
Sulfo-Cy5	**38f**	5.66 ± 0.02 (2188)	5.33 ± 0.12 (4677)	**39f**	5.66 ± 0.06 (2188)	6.09 ± 0.26 (813)
(-6.92)						

a Binding affinity (p*K*_D_/*K*_D_) determined from saturation binding curves in NanoBRET saturation binding assay conducted in Nluc-H_1_R expressing HEK293T cells. The data shown are the means ± SEM from four separate experiments.

b Binding affinity (p*K*_B_*/K*_B_) calculated using the Gaddum equation from the shift in EC_25_ obtained from the histamine stimulated dose-response curve in the presence of 1 μM of the antagonist obtained from a Ca^2+^ mobilization assay performed on human H_1_R-expressing CHO cells. The data shown are the means ± SEM from four separate experiments. ‘-’ = not determined.

c cLogP values of the fluorophore moiety up to and including the amide bond which serves as the point of attachment, determined from ChemBioDraw 19.1.

These data are remarkable and indicate that the fluorophore moiety itself has a significant impact on the binding affinity of our H_1_R fluorescent ligands irrespective of the peptide linker composition, with the BODIPY 630/650 fluorophore significantly contributing toward the high binding affinity of our series of fluorescent ligands.

BODIPY FL-based fluorescent ligands (**38d-e** and **39d-e**) and their corresponding Sulfo-Cy5-based fluorescent ligands (**38f** and **39f**) had similarly low binding affinity at Nluc-H_1_R (*K*_D_ < 1 μM), despite large differences in terms of the physicochemical properties and size of their respective fluorophores in which the BODIPY FL fluorophore is small and lipophilic, whereas the Sulfo-Cy5 fluorophore is relatively larger and hydrophilic. This might suggest that the lipophilic contribution of the fluorophore alone does not sufficiently account for observed increases in affinity. Fluorophore size and possible shape also appear to be important factors; thus, it seems less likely that non-specific interactions are governing the observed fluorophore SAR (e.g., simple insertion of the fluorophore into the lipophilic membrane environment). It may be that the fluorophore is able to make specific binding interactions with parts of the receptor – a concept that has not been extensively explored previously. Accordingly, we sought to rationalize the observed SAR through molecular docking studies (see section below and Figures 8–10). It is interesting to note that the observed effects of the fluorophore on the fluorescent ligand binding affinity was not observed in a study involving six adenosine A_2A_ receptor-selective fluorescent ligands, which exhibited affinities (*K*_D_) ranging from 20 to 83 nM despite significant differences in their fluorophores (BODIPY 630/650, BODIPY 630/650-X, BODIPY FL, BODIPY FL-X, Sulfo-Cy5, Alexa Fluor 647).^56^ This suggests that the effects of the fluorophore on fluorescent ligand binding affinity cannot be assumed and are not translatable to ligands targeting all GPCRs.

**Figure 4:**
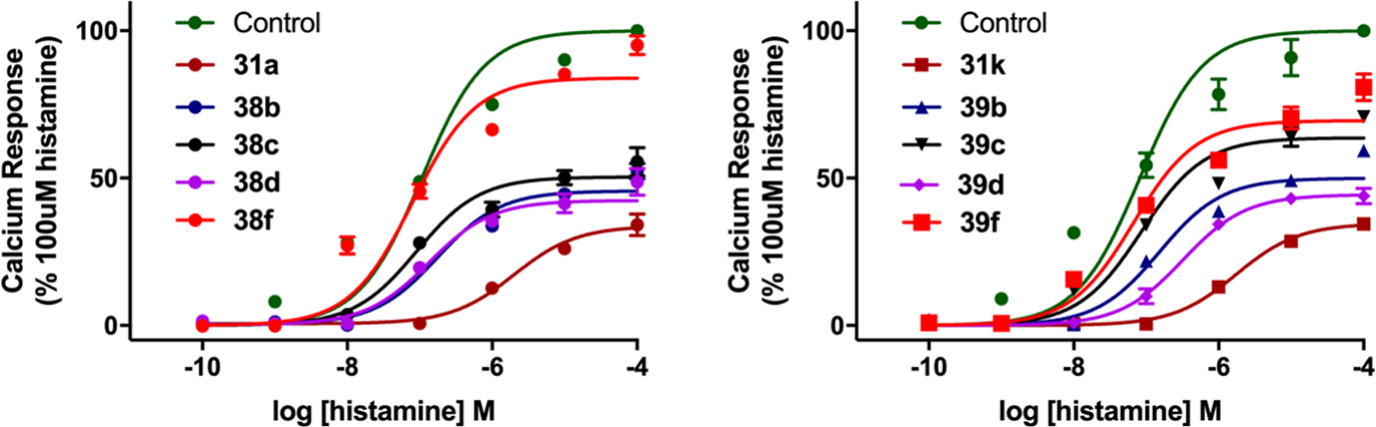
Depression of E_max_ and rightward shift of histamine mediated calcium release response curves in the presence of the fluorescent ligands at a concentration of 1 μM. Data were normalized to basal (in the absence of histamine or antagonist) and 100 μM histamine for each experiment. The data shown represent the mean ± SEM of four experiments performed in duplicate.

We conducted a secondary assay to determine the binding affinity of ten selected fluorescent ligands (**31a**, **31k**, **38b**–**d**, **38f**, **39b**–**d**, **39f**) in a Ca^2+^ mobilization assay performed on H_1_R-expressing Chinese hamster ovary (CHO) cells, in order to confirm our observation in a functional paradigm. All 10 fluorescent ligands shifted the histamine-mediated concentration–response curves rightward, indicating receptor antagonism (Figure 4). Maximum Ca^2+^ response (*E*_max_) was not achieved in the presence of all fluorescent ligands due to the transient nature of the agonist response and the consequent non-equilibrium kinetics of the calcium mobilization assay.^57^ As such, EC_25_ values were used instead of EC_50_ to estimate the binding affinity (*K*_B_) of the fluorescent compounds.^25,58,59^*K*_B_ values were in close agreement to *K*_D_ values obtained from NanoBRET saturation assay (*K*_D_), with the exception of BODIPY-FL containing fluorescent ligands **38d** and **39d**. This provides convincing evidence that the BODIPY 630/650 fluorophore is a significant contributing factor toward the synthesis of high binding affinity H_1_R fluorescent ligands (Table 4).

#### H_1_R Selectivity against the H_3_R And H_4_R Subtypes and Binding Kinetics Profile at H_1_R of **31a** and **31k**

**Figure 5:**
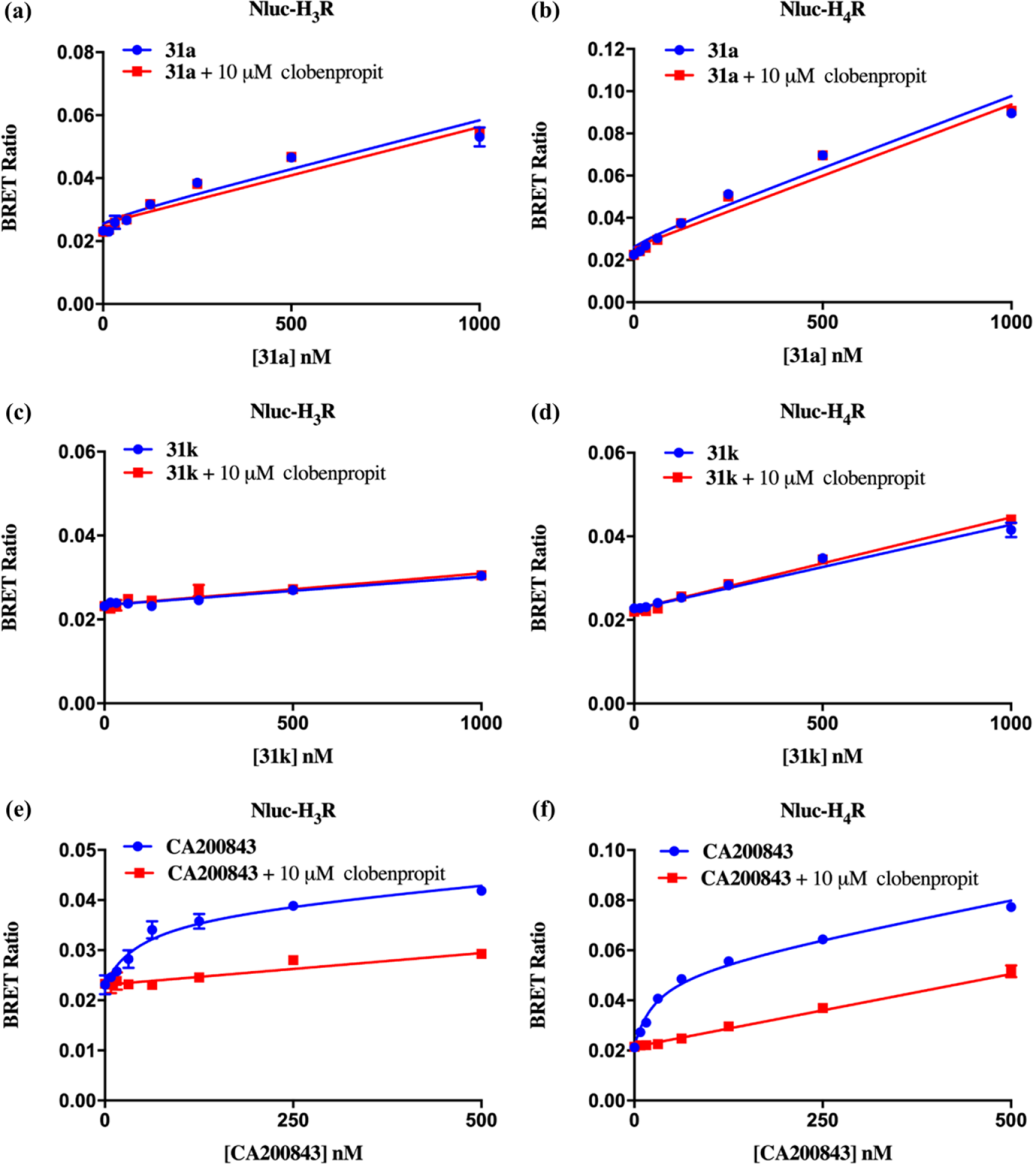
Saturation binding curves from NanoBRET experiments in cell homogenates prepared from Nluc-H_3_R (**a**, **c**, **e**) and Nluc-H_4_R expressing HEK293T cells (**b**, **d**, **f**) with increasing concentrations of fluorescent ligands **31a** (**a, b**), **31k** (**c, d**) and **CA200843** (**e, f**) in the absence (blue) or presence (red) of 10 μM clobenpropit. The data shown are representative of three independent experiments performed in duplicate and the data points are expressed in mean ± SEM and error bars are within the limits of the symbols if not shown.

Target selectivity is one of many desirable traits fluorescent ligands must possess for their wider pharmacological applications in both in vitro and in vivo paradigms. To this end, we sought to define the H_1_R selectivity of **31a** and **31k** against the H_3_R and H_4_R subtypes. As ligand binding is often driven by the orthostere, understanding the selectivity of the parent orthostere is crucial to fully defining the H_1_R selectivity of our fluorescent ligands. As such, the H_1_R selectivity profile of the VUF13816-based orthostere within our series of fluorescent ligands was determined by NanoBRET competition binding assay at H_3_R and H_4_R using **VUF14454** (radioligand binding assay p*K*_i_ at H_1_R = 8.2).^54^ NanoBRET competition binding assays involving the displacement of the commercially available clobenpropit-based BODIPY 630/650^TM^ H_3_R/H_4_R fluorescent ligand **CA200843** (HelloBio, Bristol UK) separately from Nluc-H_3_R and Nluc-H_4_R expressing HEK293T cell homogenates by increasing concentrations of **VUF14454** showed that **VUF14454** did not bind to both receptors at concentrations of up to 10 μM (Supporting InformationFigure S13), indicating high H_1_R selectivity (> 1000-fold over H_3_R and H_4_R). Subsequently, fluorescent ligand selectivity for the H_1_R over the H_3_R and H_4_R subtypes was determined by a NanoBRET saturation binding assay performed using **31a** (AA^1^ = Val, AA^2^ = Ser, AA^3^ = Asn) and **31k** (AA^1^ = Phe, AA^2^ = AA^3^ = Ala) on cell homogenates prepared from Nluc-H_3_R and Nluc-H_4_R expressing HEK293T cells. **CA200843** was included in the assay on the same assay plate as a positive control for both H_3_R and H_4_R binding experiments. Compound **31a** and **31k** showed little to no binding at both H_3_R and H_4_R at concentrations of up to 1 μM, whereas **CA200843** displayed concentration-dependent, saturable and reversible binding to Nluc-H_3_R and Nluc-H_4_R (Figure 5). The determined binding affinity of **CA200843** was in close agreement (difference of 3-fold) to values reported in existing literature^23^ (Table 5). This confirms that both **31a** and **31k** bind selectively to H_1_R over the H_3_R/H_4_R subtypes with more than or approximately 100-fold selectivity respectively and that the VUF13816-based orthostere serve as the main contributing factor for the H_1_R selectivity of **31a** and **31k**, with limited influence from the peptide linker.

**Table 5:** Binding Affinities of 31a, 31k and CA200843 at Nluc-H_3_R and Nluc-H_4_R^a^

	Nluc-H_3_R p*K*_D_ (*K*_D_ (nM))	Nluc-H_4_R p*K*_D_ (*K*_D_ (nM))
**31a**^b^	< 6 (< 1000)	< 6 (< 1000)
**31k**^b^	< 6 (< 1000)	< 6 (< 1000)
**CA200843**^c^	7.36 ± 0.05 (44)	7.51 ± 0.04 (31)

a The binding affinity of the fluorescent ligands at Nluc-H_3_R and Nluc-H_4_R was determined using a NanoBRET saturation binding assay performed on cell homogenates prepared from Nluc-H_3_R and Nluc-H_4_R expressing HEK293T cells. The data shown are the means ± SEM from three separate experiments performed in duplicate.

b The highest concentration used for the saturation binding assay was 1 μM.

c **CA200843** was commercially acquired from HelloBio (Bristol, UK). Binding affinity values reported in literature^23^ at Nluc-H_3_R and Nluc-H_4_R are 13 ± 1.9 nM (p*K*_D_ = 7.89) and 70 ± 30 nM (p*K*_D_ = 7.15) respectively.

**Figure 6:**
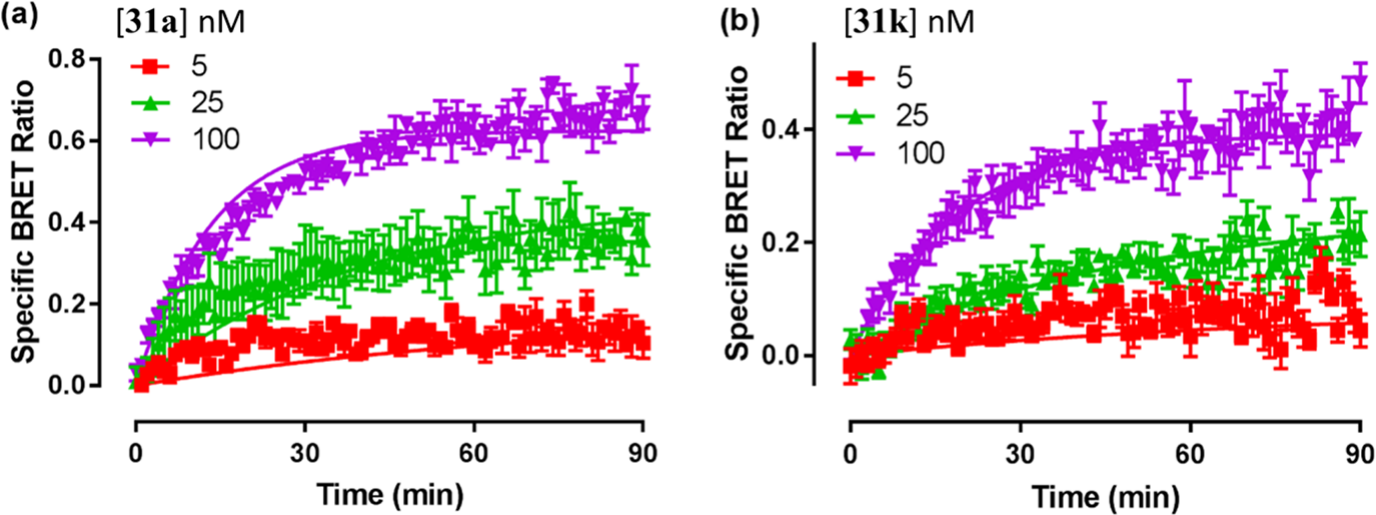
NanoBRET association kinetics assay performed on Nluc-H_1_R HEK293T cells treated with the indicated concentrations of **31a (a)** and **31k (b**), with BRET monitored at room temperature every min for 90 min. The data points shown are mean ± SEM and representative examples from five (**31a**) or four (**31k**) independent experiments performed in triplicate.

As compounds with similar binding affinities may have different binding kinetics at the target receptor, we were interested to understand the effect of peptide linker variation on the binding kinetics of **31a** and **31k** at H_1_R. This was achieved through measurement of NanoBRET association kinetics performed on Nluc-H_1_R expressed in HEK293T cells which involved real-time measurement of fluorescence/luminescence signal upon fluorescent ligand addition to the assay plate. Compounds **31a** and **31k** displayed similar binding kinetics in terms of on-rate (*k*_on_), off-rate (*k*_off_) and both possess mean residence times (MRT) of more than 1 hour, thus the composition of the peptide linker seems to have little effect on the overall binding affinity, subtype selectivity profile or binding kinetics of our peptide linker-based H_1_R fluorescent probes (Table 6, Figure 6). The long residence time of **31a** and **31k** also explains their susceptibility (due to hemi-equilibrium issues) to produce submaximal responses in the calcium mobilization assays.

**Table 6:** Association Rate (*k*_on_), Dissociation Rate (*k*_off_), Residence Time (*T*_r_) and Binding Affinity (p*K*_D_) of 31a And 31k^a^

	*k*_on_ ( x 10^6^ M^-1^min^-1^)	*k*_off_ (min^-1^)	MRT^b^ (min)	p*K*_D_^c^	n
**31a**	0.839 ± 0.103	0.014 ± 0.002	75.6 ± 10.2	7.75 ± 0.04	5
**31k**	0.686 ± 0.244	0.014 ± 0.003	78.8 ± 14.4	7.64 ± 0.12	4

a The *k*_on_, *k*_off_, *T*_r_ and p*K*_D_ of **31a** and **31k** were determined from real-time NanoBRET binding kinetic experiments conducted in Nluc-H_1_R expressing HEK293T cells. All values represent mean ± SEM from *n* separate experiments performed in triplicates.

b MRT, mean residence time (1/k_off_).

c p*K*_D_ determined from *k*_on_ and *k*_off_ (*k*_on_/*k*_off_).

### Confocal Microscopy Studies

**Figure 7:**
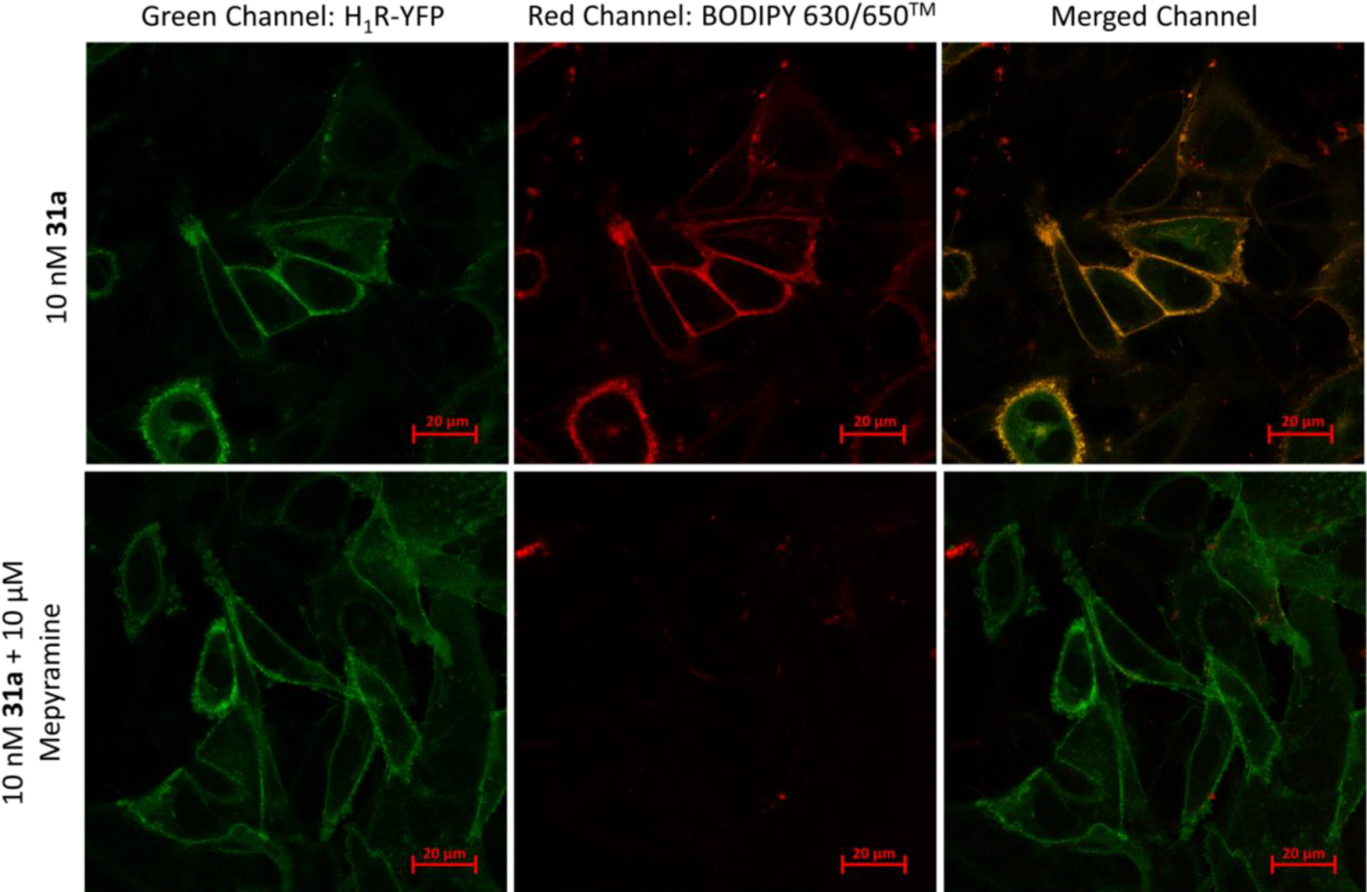
Confocal images of YFP-tagged H_1_R expressing CHO cells at 10 nM of **31a** in the absence and presence of 10 μM mepyramine observed under the red and green channels, both separately and together. The data shown are representative examples from three independent experiments performed.

Compound **31a** (AA^1^ = Val, AA^2^ = Ser, AA^3^ = Asn) was subsequently used for visualizing H_1_R at the single cell level using confocal microscopy to study the nature of the ligand binding and to determine whether cellular uptake occurred. Varying concentrations of **31a** (1 nM, 5 nM, 10 nM and 25 nM) were incubated for 30 min with CHO cells expressing yellow fluorescence protein (YFP)-tagged H_1_R at 37 °C in the presence and absence of 10 μM mepyramine pre-treatment to determine non-specific binding. Confocal imaging revealed that **31a** was localized to the plasma membrane of the cells with almost no intracellular uptake across all concentrations tested. These was attributed to the antagonist nature of **31a** (thus not promoting receptor internalization), as well as the peptide linker which reduced ligand lipophilicity and limits the ability of the membrane permeability of the fluorescent ligand. Receptor binding was specific as shown in the overlay of the green and red channels as well as the lack of signal in the red channel in the wells containing a high concentration of mepyramine (Figure 7). Compound **31a** allowed visualization of receptors on CHO cells expressing H_1_R at a concentration of 10 nM on a confocal microscope with limited non-specific signals to produce confocal images with good signal-to-noise ratio.

### Molecular Docking Studies

**Figure 8:**
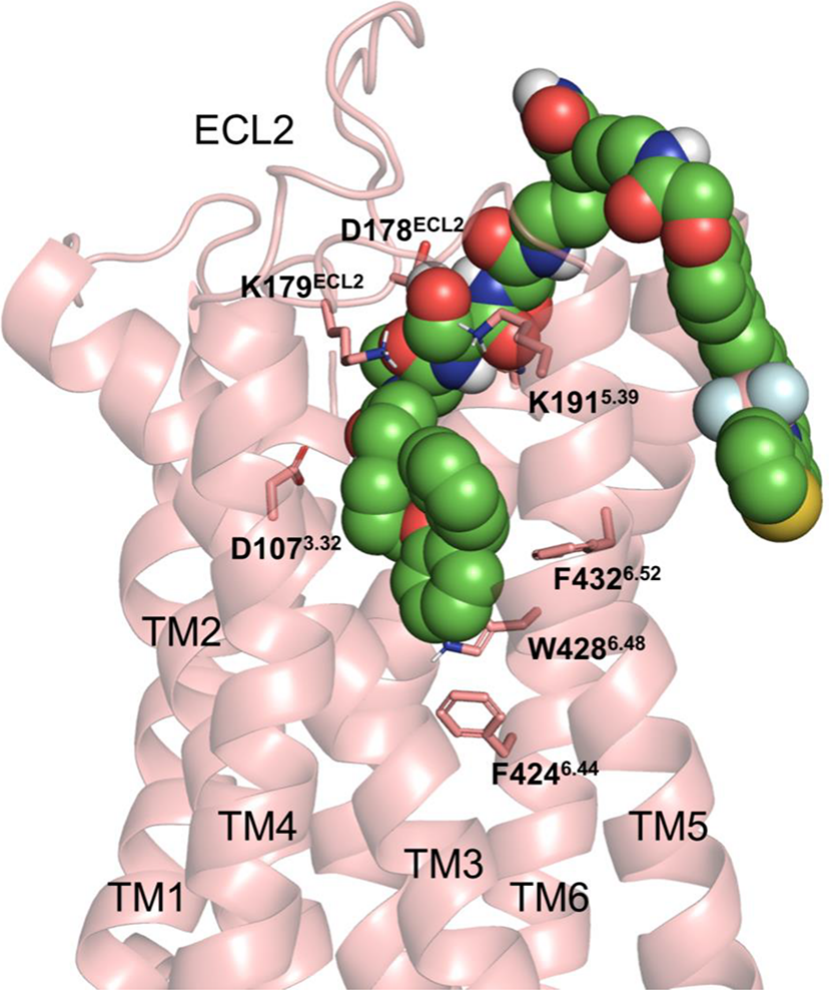
Overview of the predicted binding pose of **31a** at the H_1_R (green) determined using Glide docking. The transmembrane helices (TM) and amino acid residues which are predicted to interact with **31a** (green) are labeled.

In order to understand ligand-receptor interaction at the structural level, molecular docking studies were performed with **31a** (AA^1^ = Val, AA^2^ = Ser, AA^3^ = Asn) and the energy-minimized H_1_R crystal structure (PDB code: 3RZE, with the ligand doxepin bound)^45^ using Glide within the Schrödinger Molecular Modelling Suite. The only binding pose which formed the canonical salt bridge interaction between the protonated piperidine nitrogen present in the orthostere and D107^3.32^ as well as having good overlay between the orthostere and the co-crystallized ligand doxepin is shown in Figure 8.

**Figure 9:**
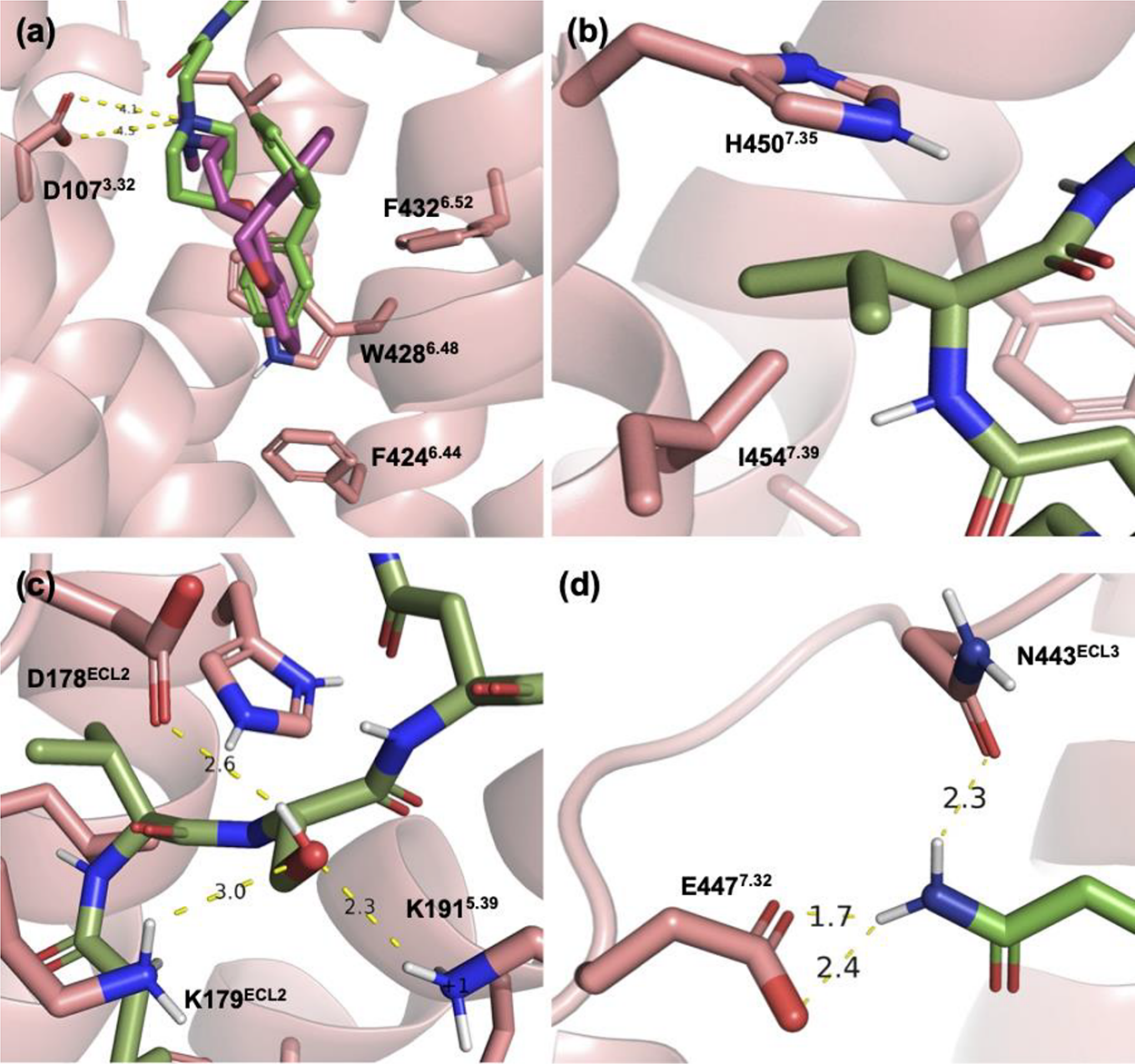
Predicted specific interactions made by 31a docked into the H_1_R crystal structure (PDB code: 3RZE).^45^ (a) The VUF13816 orthostere motif (green) is predicted to occupy the same (orthosteric) pocket as doxepin (purple) in the H_1_R crystal structure; (b) the isopropyl group of Val at AA^1^ occupies the space between H450^7.35^ and I454^7.39^; (c) the Ser hydroxyl side group at AA^2^ was predicted to engage in hydrogen bonding with the surrounding D178^ECL2^, K179^ECL2^ and K191^5.39^; (d) hydrogen bonding interactions between the side chain carboxamide moiety of Asn at AA^3^ and the amino acid residues of N443^ECL3^ and E447^7.32^.

The VUF13816 motif was predicted to occupy the same binding pocket as doxepin (i.e., the orthosteric site), whereby the aromatic moiety of the VUF13816 motif was located in close proximity to F424^6.44^, W428^6.48^ and 432^6.52^ as well as the protonated piperidine nitrogen forming a salt bridge interaction with D107^3.32^ (Figure 9a). The isopropyl side group of Val at AA^1^ within the peptide linker was predicted to occupy the space between I454^7.39^ and H450^7.35^ (Figure 9b), in which the same space was linked to the combined affinity/kinetics profile of rupatadine and its analogues.^60^ The hydroxymethyl group of Ser at AA^2^ within the peptide linker was in close proximity to D178^ECL2^ (2.6 Å), K179^ECL2^ (3.0 Å) and K191^5.39^ (2.3 Å) suggesting a network of charge-reinforced hydrogen bonding interactions (Figure 9c). The presence of the acidic D178^ECL2^ within the space predicted to be occupied by the side chain of AA^2^ on the peptide linker also provides an explanation for the lack of improved affinity when AA^2^ = Asp, due to electrostatic repulsion. The carboxamide moiety of Asn at AA^3^ was predicted to form hydrogen bond interactions with N443^ECL3^ (2.3 Å) and E447^7.32^ (1.7 Å) (Figure 9d).

Despite offering insight into the potential ligand-receptor interactions at AA^2^ (Ser) and AA^3^ (Asn) of the peptide linker, it is important to consider that the proposed receptor residues interacting with these side groups (D178^ECL2^, K179^ECL2^, K191^5.39^, N443^ECL3^ and E447^7.32^) are hydrophilic and located in or near the extracellular loop region of the receptor. As such, we propose that interactions between the peptide linker side groups and the receptor in these regions are likely formed at the expense of disrupting existing water hydrogen bond networks, thus the free energy gained from hydrogen bonding between the interaction offsets the energy required to disrupt these networks. The flexible nature of the extracellular loop region would also suggest that a fixed conformation is not likely to be adopted, making the extracellular vestibule of the receptor especially at AA^3^ hard to target for improving ligand-receptor interaction. This explains why linker optimization at AA^2^ and AA^3^ did not offer significant improvement to congener affinity, beyond those conferred by the optimization of AA.^1^

**Figure 10:**
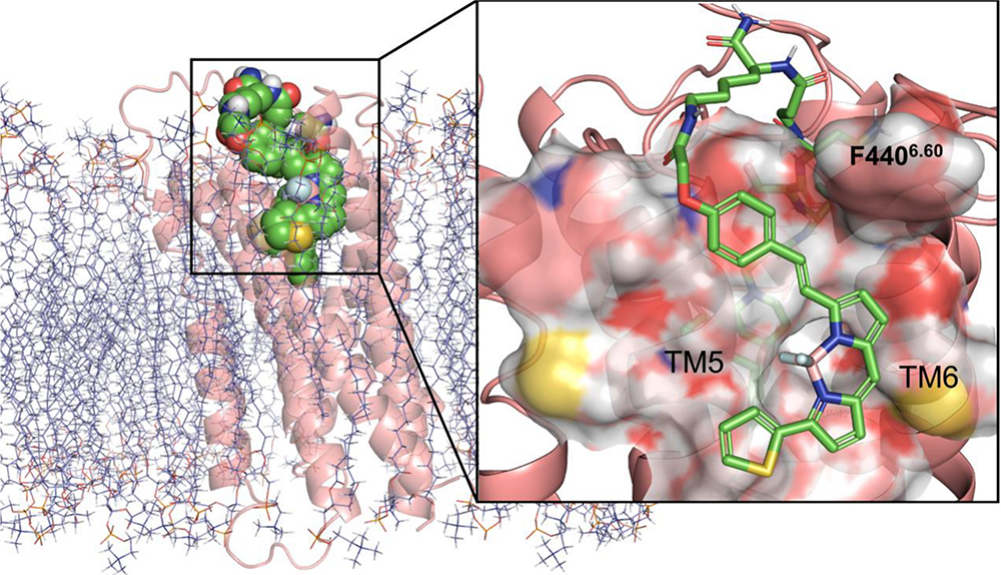
Molecular modeling of **31a** (green) bound to the human H_1_R (PDB code: 3RZE) embedded in the membrane bilayer (indigo). The BODIPY 630/650^TM^ fluorophore is predicted to be positioned within a space between TM5 and TM6, beneath the F440^6.60^ ‘lid’ which may form additional binding architecture specific to the BODIPY 630/650^TM^ fluorophore moiety surrounding the outer surface of the receptor.

Phosphatidylcholine-based phospholipid bilayer was modeled around the T4 lysozyme-truncated H_1_R crystal structure using ProBLM^61^and aligned with the predicted binding pose of **31a** using PyMOL 2.1.1 (Figure 10). The lipophilic BODIPY 630/650^TM^ fluorophore was predicted to be positioned in a space between TM5 and TM6 at the receptor outer surface, surrounded by the lipid bilayer and likely driven by the hydrophobic effect.^61^ Indeed, the gain in free energy from the hydrophobic effect attributed to the fluorophore may increase the overall ligand affinity as well as allowing the receptor to adopt non-energy minimum conformations to accommodate the various linker side groups, especially at AA^1^ where the Val side group significantly contributes to the observed linker SAR which is not seen in the corresponding fluorescent ligands. However, our data shows that H_1_R fluorescent ligands bearing lipophilic fluorophores (i.e., BODIPY B (**38c, 39c**), BODIPY FL (**38d, 39d**) and BODIPY FL-X (**38e, 39e**)) did not necessarily possess high H_1_R affinity (> 100 nM). In addition, most peptide congeners from the initial screen appear to have comparable H_1_R affinities to these fluorescent ligands, indicating that not all lipophilic fluorophores confer high H_1_R affinity, apart from the BODIPY 630/650^TM^ fluorophore. As such, we propose that although the hydrophobic effect is likely to have contributed to ligand binding, additional binding architecture surrounding the outer surface of the receptor specific to the BODIPY 630/650^TM^ fluorophore may be present. Interestingly, F440^6.60^ was predicted to be positioned directly above the BODIPY core of **31a (**Figure 10), potentially forming a ‘lid’ above the fluorophore moiety. This ‘lid’ and the space between TM5 and TM6 could well be the proposed binding architecture. This hypothesis, however, could not be investigated further without structural information determined experimentally via X-ray crystallography or cryogenic electron microscopy on the binding of our fluorescent ligands to H_1_R.

## Conclusions

In this study, we report the synthesis of a library of novel H_1_R fluorescent antagonist ligands with variations in the orthostere, peptide linker, and fluorophore moiety. Further exploration of compound **31a** reveals that it is a high-affinity H_1_R-selective fluorescent antagonist, with a *K*_D_ of 4 nM and a receptor residence time of more than 1 h at H_1_R, exhibiting at least 100-fold selectivity for H_1_R over H_3_R and H_4_R. Our initial optimization of the peptide linker through the synthesis of a library of non-fluorescent congeners established clear SAR relating to the specific amino acid residues incorporated into this region. Surprisingly, this SAR does not translate to the context of the full fluorescent ligand, having no significant impact on the potency, selectivity, or binding kinetics of our series of H_1_R fluorescent ligands. Almost all BODIPY 630/650-based fluorescent ligands (all but **22c**) synthesized and pharmacologically characterized in this study exhibited binding affinities of 4–28 nM at the H_1_R, regardless of differences in linker composition, length, and nature of the orthostere. These observations suggest that the fluorophore is a major determinant of overall ligand binding affinity in our series of H_1_R fluorescent ligands. Our subsequent systematic exploration of the effect of different fluorophores on overall fluorescent ligand affinity, using a pairwise matched approach demonstrates that SAR relating to the fluorophore itself is an important consideration when designing fluorescent ligands. To our knowledge, this exploration of fluorophore SAR in relation to target binding affinity is the first of its kind.

Molecular docking studies with **31a** provide insight into predicted ligand–receptor interactions and provide a rationale for the optimized linker employed, whereby the main improvement in congener affinity through linker optimization might be attributed to Val at AA^1^, which could effectively position itself into a space between H450^7.35^ and I454^7.39^. The fluorophore is predicted to embed itself between the H_1_R receptor outer surface and the extracellular face of phospholipid bilayer, which could significantly improve the binding of the fluorescent ligands through a combination of the hydrophobic effect and potentially fluorophore-driven ligand-receptor interaction. Experimentally determined structural information on how our fluorescent ligands bind to the H_1_R is desirable to further elucidate the role of the fluorophore in overall ligand binding to the receptor. Finally, we have demonstrated that **31a** is a valuable tool for cell imaging studies as it is capable of producing high quality images at concentrations as low as 10 nM in cell visualization experiments on the confocal microscope and thus could be a useful tool for the study of H_1_R pharmacology and receptor expression in endogenous expressing systems as well as to assess drug-target engagement in vivo.

## Experimental Section

### Chemistry General Information

All chemicals and solvents were obtained from Fischer Scientific UK, Acros Organics, Sigma-Aldrich, Merck Millipore, Fluorochem or Tocris Bioscience and used without further purification. BODIPY 630/650-X-NHS (D10000), BODIPY FL-NHS (D2184), and BODIPY FL-X-NHS (D6102) were purchased from Molecular Probes (Eugene, OR), whereas Sulfo-Cy5-NHS (23320) was purchased from Lumiprobe (Hunt Valley, MD). TLC was performed using Merck TLC Silica gel 60 Å F_254_ plates. TLC plate visualizations were conducted under UV light (256 and 366 nm). Column chromatography was carried out using Sigma Aldrich silica gel (pore size 60 Å, 230–440 mesh, 0.040–0.063 mm). LC-MS was recorded on a Shimadzu UFLCXR HPLC system combined with an Applied Biosystems MDS SCIEX API2000 electrospray ionization mass spectrometer. The column used was a Gemini 3 μm C18 110 Å, LC Column 50 × 2 mm, and the solvent system was an increasing gradient (from 5 to 95% over 5 min) of acetonitrile in water containing 0.1% formic acid, flowing at 0.5 mL/min. NMR spectra were recorded on a Bruker-AV 400 equipped with a 5 mm dual ^1^H/^13^C helium-cooled cryoprobe, which recorded the ^1^H and ^13^C NMR at 400.13 MHz and 101.62 MHz, respectively. The data was processed using iNMR (version 5.5.7), which referenced the spectra to those of the residual solvents. Chemical shifts (δ) were quoted in parts per million (ppm) and coupling constants (*J*) were reported to the nearest 0.1 Hz along with peak multiplicities using the following abbreviations: s, singlet; d, doublet; t, triplet; q, quartet; m, multiplet and br, broad. HRMS was conducted with a Bruker microTOF II mass spectrometer using electrospray ionization (ESI-TOF). Adducts within errors of ±6 ppm were reported. Analytical, semi-preparative and preparative RP HPLC were performed on a Waters 2767 sample manager coupled to Waters 2525 binary-gradient module and a Waters 2457 dual-wavelength absorbance detector or manually on a Rheodyne 7725i injector coupled to Waters 515 HPLC Pump and a Waters 996 photometric diode array detector. System 1 (for Analytical HPLC): Phenomenex Gemini 5μm reverse phase C18 column (250 × 4.6mm), a flow rate of 1.00 mL/min, and UV detection at 220, 254, and 330 nm. Linear gradient 5% to 95% solvent B over 25 min. Solvent A: 0.1% formic acid in water; solvent B: 0.1% formic acid in acetonitrile. Semi-preparative or preparative HPLC was conducted to purify the congeners and final fluorescent compound. Phenomenex Gemini 5μm reverse phase C18 column (250 x 21.2 mm), a flow rate of 20.00 mL/min, UV detection at 254 nm, and at various gradients of Solvent A:0.05% TFA in water; solvent B: 0.05% TFA in acetonitrile. System 2: 5% to 65% solvent B over 15 min. System 3: 20% to 50% solvent B over 10 min, System 4: 40% to 70% solvent B over 10 min. Phenomenex Gemini 5 μm reverse phase C18 column (250 × 10 mm), a flow rate of 5.00 mL/min, UV detection at 254 nm and at various gradients of Solvent A: 0.1% formic acid in water; solvent B: 0.1% formic acid in acetonitrile. System 5: 5% to 50% solvent B over 15 min. System 6: 30% to 60% solvent B over 15 min. System 7: 20% to 50% solvent B over 15 min. All pharmacologically tested compounds are >95% pure by HPLC.

### General Procedure 1: Solid Phase Peptide Synthesis for **8a**–**j**, **9a**–**i**, **10a**–**i**, **11a**,**b**, **21a**–**c**, **27a**–**d**, and **29a**–**k**

Fmoc-protected rink amide resin (Sigma Aldrich 533935; 50 mg, 0.05 mmol (reported resin loading of 1 mmol/g of resin)) was weighed and swelled in CH_2_Cl_2_ (5 mL) at rt overnight. The swollen resin was transferred to a small glass column, and CH_2_Cl_2_ was removed by drainage. The required oligopeptide was assembled on the resin in the following sequence of phases: loading phase, coupling phase × *n*, and capping phase, where *n* is the number of amino acids to be incorporated. Loading phase: (Step 1) The resin was washed with DMF (3 × 5 mL) and treated with 20% piperidine in DMF (2 mL) at rt for 5 min with agitation through gentle N_2_ bubbling. This procedure was repeated twice before proceeding with the next step. (Step 2) The resin was washed with DMF (3 × 5 mL) and treated with a coupling mixture of the required Fmoc-amino acid (**8a**–**j**, **9a**–**i**, **10a**–**i**: Fmoc-D-Phe-OH; **11a,b**: Fmoc-L-Phe-OH; **21a**–**c**: Fmoc-3-azido-l-alanine; **27a**–**d**: Fmoc-5-azido-l-norvaline; **29a**–**k**: Fmoc-Lys(Boc)-OH) (4 equiv, 0.20 mmol), HBTU (4 equiv, 76 mg, 0.20 mmol), HOBt hydrate (2 equiv, 16 mg, 0.10 mmol), and DIPEA (8 equiv, 70 μL, 0.40 mmol) in DMF (500 μL) at rt for 2 h. This procedure was repeated once before proceeding with the coupling phase. Coupling phase: (Step 1) The resin was washed with DMF (3 × 5 mL) and treated with 20% piperidine in DMF (2 mL) at rt for 5 min with agitation through gentle N_2_ bubbling. This procedure was repeated twice before proceeding with the next step. (Step 2) The resin was washed with DMF (3 × 5 mL) and treated with a coupling mixture of the required Fmoc-amino acid (4 equiv, 0.20 mmol), HBTU (4 equiv, 76 mg, 0.20 mmol), HOBt hydrate (2 equiv, 16 mg, 0.10 mmol), and DIPEA (8 equiv, 70 μL, 0.40 mmol) in DMF (500 μL) at rt for 2 h. This procedure was repeated once before the start of a new coupling phase. Capping phase: The resin was washed with DMF (3 × 5 mL) and treated with 20% piperidine in DMF (2 mL) at rt for 5 min with agitation through gentle N_2_ bubbling. This procedure was performed thrice before proceeding with the next step. Subsequently, the resin was washed with DMF (3 × 5 mL) and treated with a coupling mixture of the required carboxylic acid (**8a**–**j**, **9a**–**i**, **10a**–**i**, **11a**,**b**, **21a**–**c**, **27a**,**b**, **29a**–**k**: **7**; **27c**,**d**: **26**) (2 equiv, 0.10 mmol), HBTU (2 equiv, 38 mg, 0.10 mmol), HOBt hydrate (1 equiv, 8 mg, 0.05 mmol) and DIPEA (4 equiv, 35 μL, 0.2 mmol) in DMF (500 μL) at rt for 4 h.

The resin was washed with DMF (3 × 5 mL), DCM (1 × 5 mL), and hexane (1 × 5 mL) successively and left to dry overnight. The resin was subsequently treated with a cleavage mixture consisting of 90% TFA:10% water (3 mL), and the suspended resin was stirred by gentle N_2_ bubbling at rt for 3 h. The reaction mixture was pipetted into a round-bottom flask, concentrated over a rotary evaporator to remove TFA, re-dissolved in DMSO, filtered, and purified by semi-preparative or preparative HPLC (**8a**–**j**, **9a**–**i**, **10a**–**i**, **11a**,**b**: System 2; **21a**–**c**, **27a**–**d**: System 5; **29a**–**k**: System 3), and subsequent lyophilization afforded the product*.*

### General Procedure 2: Fluorophore Conjugation via CuAAC for **22a**–**c** and 2**8a**–**d**

The required azide congener (**21a-c** and **28a-d**; 1 equiv) was dissolved in DMSO (250 μL/μmol of congener) and transferred to a 5 mL microwave vial. (*E*)-5,5-Difluoro-7-(4-(prop-2-yn-1-yloxy)styryl)-3-(thiophen-2-yl)-5*H*-5λ^4^-dipyrrolo[1,2-*c*:2’,1’-*f*][1,3,2]diazaborinin-4-ium **(20)** (1.1 equiv), CuSO_4_·5H_2_O (3 equiv), and sodium ascorbate (9 equiv) were dissolved in DMSO (250 μL/μmol of congener) in a scintillation vial and sonicated for 5 min. The sonicated mixture was added to the vial, followed by three drops of 2,6-lutidine, and the reaction mixture stirred at rt overnight. The reaction mixture was purified using Semi-/Prep HPLC (system 7) and subsequent lyophilization afforded the product.

### General Procedure 3: Fluorophore Conjugation Via Amide Bond Formation with HATU for **31a**–**k**, **38b**,**c** and **39b**,**c**

The desired amine congener (**29a-k**; 1 equiv) was dissolved in DMF (1 mL/μmol of congener), treated with the desired fluorophore (0.9 equiv) (**31a-k**: **30**;^49^**38b,c**: **35**; **39b,c**: **37**), HATU (1.2 equiv), and DIPEA (6 equiv), and the reaction mixture was stirred at rt for 1 hr in the dark. The reaction mixture was diluted with DMF (1 mL) and purified using semi-preparative or preparative HPLC (**31a-k**: system 4; **38b**, **39b**: system 6; **38c**, **39c**: system 7), and subsequent lyophilization afforded the product.

### General Procedure 4: Fluorophore Conjugation Via Amide Bond Formation with NHS Esters for **38a**, **38d**–**f**, **39a**, and **39d**–**f**

The desired amine congener **27a** or **27k** (1 equiv) was dissolved in DMF (1 mL) and treated with the desired fluorophore (1 equiv) (**38a**, **39a**: BODIPY 630/650-X-NHS; **38d**, **39d**: BODIPY-FL-NHS; **38e**, **39e**: BODIPY FL-X-NHS; **38f**, **39f**: Sulfo-Cy5-NHS) and a few drops of DIPEA. The reaction mixture was stirred at rt for 2 h in the dark. The reaction mixture was purified using semi-preparative HPLC (system 7), and subsequent lyophilization afforded the product.

#### *tert*-Butyl 4-(2-benzylphenoxy)piperidine carboxylate (**4**)^25^

2-Benzylphenol (**2**) (1000 mg, 5.43 mmol) and 1092 mg (5.43 mmol) of 1-Boc-4-hydroxypiperidine (**3**) were dissolved in dry THF (5 mL) in a dry 25 mL 2-necked RBF treated with 1566 mg (5.97 mmol) of triphenylphosphine and 1175 μL (5.97 mmol) of diisopropyl azodicarboxylate, and the reaction mixture was stirred at rt overnight. The reaction mixture was filtered, and the filtrate was triturated with hexane. The resulting suspension was filtered, and the filtrate was concentrated over rotary evaporator. The resulting mixture was re-dissolved in EtOAc (100 mL), washed once with 1 M NaOH followed by saturated brine, dried over MgSO_4_, and filtered, and the filtrate was concentrated over a rotary evaporator and purified by silica gel column chromatography (1-50% EtOAc/PE + 1% Et_3_N) to afford a pale yellow oil (957 mg), which consisted of a mixture of the desired product and the elimination product *tert*-butyl 3,6-dihydropyridine-1(2*H*)-carboxylate in a molar ratio of 1:0.274 as determined by ^1^H NMR (88 wt % of desired product, 2.29 mmol, 42%). The product mixture was used in the next step of the synthesis without further purification. ^1^H NMR (400 MHz, CDCl_3_) δ 7.40–7.02 (m, 7H, Ar-C**H**), 7.02–6.72 (m, 2H, Ar-C**H**), 4.52–4.46 (m 1H, −C**H**), 3.99 (s, 2H, benzyl C**H_2_**), 3.53–3.42 (m, 2H, piperidine C**H_2_**), 3.42–3.28 (m, 2H, piperidine C**H_2_**), 1.90–1.77 (m, 2H, piperidine C**H_2_**), 1.77–1.61 (m, 2H, piperidine C**H_2_**), 1.48 (s, 9H, −C**(**C**H_3_)_3_**). ^13^C NMR (101 MHz, CDCl_3_) δ 171.2, 154.9, 141.2, 131.1, 130.6, 128.9, 128.3, 127.5, 125.9, 120.6, 112.5, 79.6, 71.4, 40.5, 36.6, 30.5, 28.5. LC-MS *m/z* calculated (M+H)^+^ for C_23_H_29_NO_3_ = 368.2, found = 368.1; *t*_R_*=* 3.35 min.

#### 4-(2-Benzylphenoxy)piperidine (**5**)^25^

*tert*-Butyl 4-(2-benzylphenoxy)piperidine carboxylate (**4**) (778 mg (88 wt % of 884 mg), 2.12 mmol) was dissolved in DCM (10 mL) and treated with TFA (1 mL, 13.1 mmol), and the reaction mixture stirred at rt overnight. The reaction mixture was concentrated over a rotary evaporator and re-dissolved in EtOAc. The organic layer was washed three times with NaHCO_3_ followed by saturated brine, dried over MgSO_4_, and filtered, and the filtrate was concentrated over a rotary evaporator to afford a yellow oil (525 mg, 1.97 mmol, 93%). ^1^H NMR (400 MHz, CDCl_3_) δ 7.30–7.23 (m, 2H, Ar-C**H**), 7.23–7.10 (m, 5H, Ar-C**H**), 6.93–6.79 (m, 2H, Ar-C**H**), 4.48–4.38 (m, 1H, −C**H**), 3.99 (s, 2H, benzyl C**H_2_**), 3.06–2.97 (m, 2H, Pip C**H_2_**), 2.91 (br s, 1H, N**H**), 2.79–2.69 (m, 2H, piperidine C**H_2_**), 2.03–1.91 (m, 2H, piperidine C**H_2_**), 1.74–1.61 (m, 2H Pip C**H_2_**). ^13^C NMR (101 MHz, CDCl_3_) δ 155.0, 141.4, 131.1, 130.7, 129.0, 128.3, 127.5, 125.9, 120.6, 112.7, 72.0, 43.2, 36.6, 31.6. LC-MS *m/z* calculated (M+H)^+^ for C_18_H_21_NO = 268.2, found = 267.9; *t*_R_*=* 2.22 min.

#### Methyl 3-(4-(2-Benzylphenoxy)piperidin-1-yl)propanoate (**6**)^25^

4-(2-Benzylphenoxy)piperidine (**5**) (525 mg, 1.97 mmol) was dissolved in 1,2-dichloroethane (2 mL) and treated with methyl acrylate (890 μL, 10mmol), and the reaction mixture was stirred at 70 °C under reflux for 4 h. The reaction mixture was concentrated over a rotary evaporator and purified by silica gel column chromatography (40% EtOAc/PE + 2% Et_3_N) to afford a pale-yellow oil (454 mg, 1.29 mmol, 66%). ^1^H NMR (400 MHz, CDCl_3_) δ 7.39–7.03 (m, 7H, Ar-C**H**), 6.97–6.74 (m, 2H, Ar-C**H**), 4.42–4.28 (m, 1H, −C**H**), 3.98 (s, 2H, benzyl C**H_2_**), 3.69 (s, 3H, −COOC**H_3_**), 2.72–2.63 (m, 2H, Pip C**H_2_**), 2.67 (t, *J* = 7.4 Hz, 2H, −C**H_2_**−), 2.50 (t, *J* = 7.4 Hz, 2H, −C**H_2_**−), 2.38–2.28 (m, 2H, Pip C**H_2_**), 1.98–1.87 (m, 2H, Pip C**H_2_**), 1.85–1.73 (m, 2H, Pip C**H_2_**). ^13^C NMR (101 MHz, CDCl_3_) δ 173.1, 155.2, 141.4, 131.0, 130.6, 129.0, 128.3, 127.4, 125.8, 120.4, 112.6, 71.7, 53.7, 51.8, 50.1, 36.5, 32.4, 30.8. LC-MS *m/z* calculated (M+H)^+^ for C_22_H_27_NO_3_ = 354.2, found = 353.8; *t*_R_*=* 2.32 min.

#### 3-(4-(2-Benzylphenoxy)piperidin-1-yl)propanoic Acid (**7**)^25^

Methyl 3-(4-(2-benzylphenoxy)piperidin-1-yl)propanoate (**6**) (342 mg, 0.97 mmol) was dissolved in THF (5 mL) and cooled in an ice bath. The reaction mixture was treated with NaOH (120 mg, 3 mmol) and dissolved in de-ionized water (4 mL). The reaction mixture was stirred for 5 h in an ice bath. The reaction mixture was concentrated over a rotary evaporator, and the pH of the reaction mixture was adjusted to 6 by gradual addition of 2 M aqueous HCl. The reaction mixture was diluted with water and extracted three times with CHCl_3_. The combined organic layer was washed with saturated brine, dried over MgSO_4_, and filtered, and the filtrate was concentrated over a rotary evaporator to afford a pale-yellow solid (244 mg, 0.72 mmol, 74%). ^1^H NMR (400 MHz, CD_3_OD) δ 7.32–7.19 (m, 4H, Ar-C**H**), 7.22–7.10 (m, 3H, Ar-C**H**), 7.00–6.90 (m, 2H, Ar-C**H**), 4.70 (p, *J* = 3.8 Hz, 1H, −C**H**), 4.01 (s, 2H, benzyl C**H_2_**), 3.21–3.10 (m, 2H, −C**H_2_**–CH_2_–COOH), 3.03 (t, *J* = 6.7 Hz, 2H, −C**H_2_**–COOH), 2.84–2.59 (m, 2H, piperidine C**H_2_**), 2.12–1.90 (m, 4H, 2 × piperidine C**H_2_**). ^13^C NMR (101 MHz, CD_3_OD) δ 176.7, 155.6, 143.4, 132.9, 130.8, 129.6, 129.4, 129.1, 127.1, 122.0, 113.2, 67.6, 54.9, 49.9, 37.9, 30.7, 28.6. LC-MS *m/z* calculated (M+H)^+^ for C_21_H_25_NO_3_ = 340.2, found = 339.8; *t*_R_*=* 2.32 min.

#### (*S*)-*N*-((*R*)-1-Amino-1-oxo-3-phenylpropan-2-yl)-2-((*S*)-2-((*S*)-2-(3-(4-(2-benzylphen oxy)piperidin-1-yl)-propana-mido)propanamido)propanamido)propanamide (**8a**)

The title compound was synthesized following the method described in general procedure 1, using Fmoc-d-Phe-OH (78 mg, 0.20 mmol) for the loading phase; Fmoc-Ala-OH (62 mg, 0.20 mmol), Fmoc-Ala-OH (62 mg, 0.20 mmol), and Fmoc-Ala-OH (62 mg, 0.20 mmol) for three separate coupling phases in the stated sequence; **7** (34 mg, 0.10 mmol) for the capping phase to afford a colorless solid (0.21 mg, 0.30 μmol, 1%). HRMS (Bruker MicroTOF)(*m/z*): Calculated (M+H)^+^ for C_39_H_50_N_6_O_6_ = 699.3865; measured = 699.3871. Analytical HPLC (system 1): 98% purity; *t*_R_ = 13.66 min.

#### (*S*)-*N*-((*R*)-1-Amino-1-oxo-3-phenylpropan-2-yl)-2-((*S*)-2-(2-(3-(4-(2-benzylphenoxy)piperidin-1-yl)propanamido)acetamido)propanamido)propanamide (**8b**)

The title compound was synthesized following the method described in general procedure 1, using Fmoc-d-Phe-OH (78 mg, 0.20 mmol) for the loading phase; Fmoc-Ala-OH (62 mg, 0.20 mmol), Fmoc-Ala-OH (62 mg, 0.20 mmol), and Fmoc-Gly-OH (60 mg, 0.20 mmol) for three separate coupling phases in the stated sequence; **7** (34 mg, 0.10 mmol) to afford a colorless solid (0.33 mg, 0.48 μmol, 1%). HRMS (Bruker MicroTOF)(*m/z*): Calculated (M+H)^+^ for C_38_H_48_N_6_O_6_ = 685.3708; measured = 685.3716. Analytical HPLC (system 1): 98% purity; *t*_R_*=* 13.73 min.

#### (*S*)-*N*-((*S*)-1-(((*S*)-1-(((*R*)-1-Amino-1-oxo-3-phenylpropan-2-yl)amino)-1-oxopropan-2-yl)amino)-1-oxopropan-2-yl)-2-(3-(4-(2-benzylphenoxy)piperidin-1-yl)propanamido)-3-methylbutanamide (**8c**)

The title compound was synthesized following the method described in general procedure 1, using Fmoc-d-Phe-OH (78 mg, 0.20 mmol) for the loading phase; Fmoc-Ala-OH (62 mg, 0.20 mmol), Fmoc-Ala-OH (62 mg, 0.20 mmol), and Fmoc-Val-OH (68 mg, 0.20 mmol) for three separate coupling phases in the stated sequence; **7** (34 mg, 0.10 mmol) for the capping phase to afford a colorless solid (0.52 mg, 0.72 μmol, 2%). HRMS (Bruker MicroTOF)(*m/z*): Calculated (M+H)^+^ for C_41_H_54_N_6_O_6_ = 727.4178; measured = 727.4188. Analytical HPLC (system 1): 98% purity; *t*_R_*=* 14.21 min.

#### (*S*)-*N*-((*S*)-1-(((*S*)-1-(((*R*)-1-Amino-1-oxo-3-phenylpropan-2-yl)amino)-1-oxopropan-2-yl)amino)-1-oxopropan-2-yl)-2-(3-(4-(2-benzylphenoxy)piperidin-1-yl)propanamido)-3-phenylpropanamide (**8d**)

The title compound was synthesized following the method described in general procedure 1, using Fmoc-d-Phe-OH (78 mg, 0.20 mmol) for the loading phase; Fmoc-Ala-OH (62 mg, 0.20 mmol), Fmoc-Ala-OH (62 mg, 0.20 mmol), and Fmoc-l-Phe-OH (78 mg, 0.20 mmol) for three separate coupling phases in the stated sequence; **7** (34 mg, 0.10 mmol) for the capping phase to afford a colorless solid (0.28 mg, 0.36 μmol, 1%). HRMS (Bruker MicroTOF)(*m/z*): Calculated (M+H)^+^ for C_45_H_54_N_6_O_6_ = 775.4178; measured = 775.4154. Analytical HPLC (system 1): 98% purity; *t*_R_*=* 14.08 min.

#### (*S*)-*N*-((*S*)-1-(((*S*)-1-(((*R*)-1-Amino-1-oxo-3-phenylpropan-2-yl)amino)-1-oxopropan-2-yl)amino)-1-oxopropan-2-yl)-2-(3-(4-(2-benzylphenoxy)piperidin-1-yl)propanamido)-3-(4-hydroxyphenyl)propanamide (**8e**)

The title compound was synthesized following the method described in general procedure 1, using Fmoc-d-Phe-OH (78 mg, 0.20 mmol) for the loading phase; Fmoc-Ala-OH (62 mg, 0.20 mmol), Fmoc-Ala-OH (62 mg, 0.20 mmol), and Fmoc-Tyr(tBu)-OH (92 mg, 0.20 mmol) for three separate coupling phases in the stated sequence; **7** (34 mg, 0.10 mmol) for the capping phase to afford a colorless solid (0.26 mg, 0.33 μmol, 1%). HRMS (Bruker MicroTOF)(*m/z*): Calculated (M+H)^+^ for C_45_H_54_N_6_O_7_ = 791.4127; measured = 791.4130. Analytical HPLC (*System 1*): 98% purity; *t*_R_*=* 14.08 min.

#### (*S*)-*N*-((*S*)-1-(((*S*)-1-(((*R*)-1-Amino-1-oxo-3-phenylpropan-2-yl)amino)-1-oxopropan-2-yl)amino)-1-oxopropan-2-yl)-2-(3-(4-(2-benzylphenoxy)piperidin-1-yl)propanamido)-3-hydroxypropanamide (**8f**)

The title compound was synthesized following the method described in general procedure 1, using Fmoc-d-Phe-OH (78 mg, 0.20 mmol) for the loading phase; Fmoc-Ala-OH (62 mg, 0.20 mmol), Fmoc-Ala-OH (62 mg, 0.20 mmol), and Fmoc-Ser(tBu)-OH (77 mg, 0.20 mmol) for three separate coupling phases in the stated sequence; **7** (34 mg, 0.10 mmol) for the capping phase to afford a colorless solid (0.46 mg, 0.64 μmol, 2%). HRMS (Bruker MicroTOF)(*m/z*): Calculated (M+H)^+^ for C_39_H_50_N_6_O_7_ = 715.3814; measured = 715.3835. Analytical HPLC (system 1): 98% purity; *t*_R_*=* 13.42 min.

#### (*S*)-*N*-((S)-1-(((*S*)-1-(((*R*)-1-Amino-1-oxo-3-phenylpropan-2-yl)amino)-1-oxopropan-2-yl)amino)-1-oxopropan-2-yl)-2-(3-(4-(2-benzylphenoxy)piperidin-1-yl)-propanamido)succinamide (**8g**)

The title compound was synthesized following the method described in general procedure 1, using Fmoc-d-Phe-OH (78 mg, 0.20 mmol) for the loading phase; Fmoc-Ala-OH (62 mg, 0.20 mmol), Fmoc-Ala-OH (62 mg, 0.20 mmol), and Fmoc-Asn(Trt)-OH (120 mg, 0.20 mmol) for three separate coupling phases in the stated sequence; **7** (34 mg, 0.10 mmol) for the capping phase to afford a colorless solid (0.50 mg, 0.67 μmol, 2%). HRMS (Bruker MicroTOF)(*m/z*): Calculated (M+H)^+^ for C_40_H_51_N_7_O_7_ = 742.3923; measured = 742.3923. Analytical HPLC (system 1): 97% purity; *t*_R_*=* 13.35 min.

#### (*S*)-*N*-((S)-1-(((*S*)-1-(((*R*)-1-Amino-1-oxo-3-phenylpropan-2-yl)amino)-1-oxopropan-2-yl)amino)-1-oxopropan-2-yl)-2-(3-(4-(2-benzylphenoxy)piperidin-1-yl)propanamido)-3-(1*H*-imidazol-5-yl)propanamide (**8h**)

The title compound was synthesized following the method described in general procedure 1, using Fmoc-d-Phe-OH (78 mg, 0.20 mmol) for the loading phase; Fmoc-Ala-OH (62 mg, 0.20 mmol), Fmoc-Ala-OH (62 mg, 0.20 mmol), and Fmoc-His(Trt)-OH (124 mg, 0.20 mmol) for three separate coupling phases in the stated sequence; **7** (34 mg, 0.10 mmol) for the capping phase to afford a colorless solid (0.27 mg, 0.34 μmol, 1%). HRMS (Bruker MicroTOF)(*m/z*): Calculated (M+Na)^+^ for C_42_H_52_N_8_O_6_ = 787.3882; measured = 787.3882. Analytical HPLC (system 1): 95% purity; *t*_R_*=* 11.82 min.

#### (*S*)-6-Amino-*N*-(*(S*)-1-(((*S*)-1-(((*R*)-1-amino-1-oxo-3-phenylpropan-2-yl)amino)-1-oxopropan-2-yl)-amino)-1-oxopropan-2-yl)-2-(3-(4-(2-benzylphenoxy)piperidin-1-yl)-propanamido)hexanamide (**8i**)

The title compound was synthesized following the method described in general procedure 1, using Fmoc-d-Phe-OH (78 mg, 0.20 mmol) for the loading phase; Fmoc-Ala-OH (62 mg, 0.20 mmol), Fmoc-Ala-OH (62 mg, 0.20 mmol), and Fmoc-Lys(Boc)-OH (94 mg, 0.20 mmol) for three separate coupling phases in the stated sequence; **7** (34 mg, 0.10 mmol) for the capping phase to afford a colorless solid (0.53 mg, 0.70 μmol, 2%). HRMS (Bruker MicroTOF)(*m/z*): Calculated (M+H)^+^ for C_42_H_57_N_7_O_6_ = 756.4443; measured = 756.4428. Analytical HPLC (system 1): 98% purity; *t*_R_*=* 11.77 min.

#### (*S*)-4-(((*S*)-1-(((*S*)-1-(((*R*)-1-Amino-1-oxo-3-phenylpropan-2-yl)amino)-1-oxopropan-2-yl)amino)-1-oxopropan-2-yl)amino)-3-(3-(4-(2-benzylphenoxy)piperidin-1-yl)propanamido)-4-oxobutanoic Acid (**8j**)

The title compound was synthesized following the method described in general procedure 1, using Fmoc-d-Phe-OH (78 mg, 0.20 mmol) for the loading phase; Fmoc-Ala-OH (62 mg, 0.20 mmol), Fmoc-Ala-OH (62 mg, 0.20 mmol), and Fmoc-Asp(OtBu)-OH (83 mg, 0.20 mmol) for three separate coupling phases in the stated sequence; **7** (34 mg, 0.10 mmol) for the capping phase to afford a colorless solid (0.67 mg, 0.90 μmol, 1%). HRMS (Bruker MicroTOF)(*m/z*): Calculated (M+H)^+^ for C_40_H_50_N_6_O_8_ = 743.3763; Measured = 743.3765. Analytical HPLC (system 1): 98% purity; *t*_R_*=* 13.60 min.

#### (*S*)-*N*-(2-(((*S*)-1-(((*R*)-1-Amino-1-oxo-3-phenylpropan-2-yl)amino)-1-oxopropan-2-yl)amino)-2-oxoethyl)-2-(3-(4-(2-benzylphenoxy)piperidin-1-yl)propanamido)-3-methylbutanamide (**9a**)

The title compound was synthesized following the method described in general procedure 1, using Fmoc-d-Phe-OH (78 mg, 0.20 mmol) for the loading phase; Fmoc-Ala-OH (62 mg, 0.20 mmol), Fmoc-Gly-OH (60 mg, 0.20 mmol), and Fmoc-Val-OH (68 mg, 0.20 mmol) for three separate coupling phases in the stated sequence; **7** (34 mg, 0.10 mmol) for the capping phase to afford a colorless solid (1.27 mg, 1.78 μmol, 4%). HRMS (Bruker MicroTOF)(*m/z*): Calculated (M+H)^+^ for C_40_H_52_N_6_O_6_ = 713.4021; measured = 713.4048. Analytical HPLC (system 1): 97% purity; *t*_R_*=* 14.69 min.

#### (*S*)-*N*-((*S*)-1-(((*R*)-1-Amino-1-oxo-3-phenylpropan-2-yl)amino)-1-oxopropan-2-yl)-2-((*S*)-2-(3-(4-(2-benzylphenoxy)piperidin-1-yl)propanamido)-3-methylbutanamido)-3-methylbutanamide (**9b**)

The title compound was synthesized following the method described in general procedure 1, using Fmoc-d-Phe-OH (78 mg, 0.20 mmol) for the loading phase; Fmoc-Ala-OH (62 mg, 0.20 mmol), Fmoc-Val-OH (68 mg, 0.20 mmol), and Fmoc-Val-OH (68 mg, 0.20 mmol) for three separate coupling phases in the stated sequence; **7** (34 mg, 0.10 mmol) for the capping phase to afford a colorless solid (1.62 mg, 2.15 μmol, 5%). HRMS (Bruker MicroTOF)(*m/z*): Calculated (M+H)^+^ for C_43_H_58_N_6_O_6_ = 755.4491; measured = 755.4515. Analytical HPLC (system 1): 90% purity; *t*_R_*=* 15.34 min.

#### (*S*)-*N*-((*S*)-1-(((*S*)-1-(((*R*)-1-Amino-1-oxo-3-phenylpropan-2-yl)amino)-1-oxopropan-2-yl)amino)-1-oxo-3-phenylpropan-2-yl)-2-(3-(4-(2-benzylphenoxy)piperidin-1-yl)-propanamido)-3-methylbutanamide (**9c**)

The title compound was synthesized following the method described in general procedure 1, using Fmoc-d-Phe-OH (78 mg, 0.20 mmol) for the loading phase; Fmoc-Ala-OH (62 mg, 0.20 mmol), Fmoc-l-Phe-OH (78 mg, 0.20 mmol), and Fmoc-Val-OH (68 mg, 0.20 mmol) for three separate coupling phases in the stated sequence; **7** (34 mg, 0.10 mmol) for the capping phase to afford a colorless solid (1.94 mg, 2.42 μmol, 6%). HRMS (Bruker MicroTOF)(*m/z*): Calculated (M+H)^+^ for C_47_H_58_N_6_O_6_ = 803.4491; measured = 803.4510. Analytical HPLC (system 1): 98% purity; *t*_R_*=* 16.28 min.

#### (*S*)-*N*-((*S*)-1-(((*S*)-1-(((*R*)-1-Amino-1-oxo-3-phenylpropan-2-yl)amino)-1-oxopropan-2-yl)amino)-3-(4-hydroxyphenyl)-1-oxopropan-2-yl)-2-(3-(4-(2-benzylphenoxy)-piperidin-1-yl)propanamido)-3-methylbutanamide (**9d**)

The title compound was synthesized following the method described in general procedure 1, using Fmoc-d-Phe-OH (78 mg, 0.20 mmol) for the loading phase; Fmoc-Ala-OH (62 mg, 0.20 mmol), Fmoc-Tyr(tBu)-OH (92 mg, 0.20 mmol), and Fmoc-Val-OH (68 mg, 0.20 mmol) for three separate coupling phases in the stated sequence; **7** (34 mg, 0.10 mmol) for the capping phase to afford a colorless solid (2.36 mg, 2.88 μmol, 7%). HRMS (Bruker MicroTOF)(*m/z*): Calculated (M+H)^+^ for C_47_H_58_N_6_O_7_ = 819.4440; measured = 819.4457. Analytical HPLC (system 1): 96% purity; *t*_R_*=* 15.32 min.

#### (*S*)-*N*-((*S*)-1-(((*S*)-1-(((*R*)-1-Amino-1-oxo-3-phenylpropan-2-yl)amino)-1-oxopropan-2-yl)amino)-3-hydroxy-1-oxo-propan-2-yl)-2-(3-(4-(2-benzylphenoxy)piperidin-1-yl)-propanamido)-3-methylbutanamide (**9e**)

The title compound was synthesized following the method described in general procedure 1, using Fmoc-d-Phe-OH (78 mg, 0.20 mmol) for the loading phase; Fmoc-Ala-OH (62 mg, 0.20 mmol), Fmoc-Ser(tBu)-OH (77 mg, 0.20 mmol), and Fmoc-Val-OH (68 mg, 0.20 mmol) for three separate coupling phases in the stated sequence; **7** (34 mg, 0.10 mmol) for the capping phase to afford a colorless solid (2.87 mg, 3.87 μmol, 10%). HRMS (Bruker MicroTOF)(*m/z*): Calculated (M+H)^+^ for C_41_H_54_N_6_O_7_ = 743.4127; measured = 743.4153. Analytical HPLC (system 1): 98% purity; *t*_R_*=* 14.47 min.

#### (*S*)-*N*1-((*S*)-1-(((*R*)-1-Amino-1-oxo-3-phenylpropan-2-yl)amino)-1-oxopropan-2-yl)-2-((*S*)-2-(3-(4-(2-benzylphenoxy)piperidin-1-yl)propanamido)-3-methylbutanamido)succinamide (**9f**)

The title compound was synthesized following the method described in general procedure 1, using Fmoc-d-Phe-OH (78 mg, 0.20 mmol) for the loading phase; Fmoc-Ala-OH (62 mg, 0.20 mmol), Fmoc-Asn(Trt)-OH (120 mg, 0.20 mmol), and Fmoc-Val-OH (68 mg, 0.20 mmol) for three separate coupling phases in the stated sequence; **7** (34 mg, 0.10 mmol) for the capping phase to afford a colorless solid (2.86 mg, 3.72 μmol, 9%). HRMS (Bruker MicroTOF)(*m/z*): Calculated (M+H)^+^ for C_42_H_55_N_7_O_7_ = 770.4236; measured = 770.4266. Analytical HPLC (system 1): 98% purity; *t*_R_*=* 14.28 min.

#### (*S*)-*N*-((*S*)-1-(((*S*)-1-(((*R*)-1-Amino-1-oxo-3-phenylpropan-2-yl)amino)-1-oxopropan-2-yl)amino)-3-(1*H*-imidazol-5-yl)-1-oxopropan-2-yl)-2-(3-(4-(2-benzylphenoxy)piperidin-1-yl)propanamido)-3-methylbutanamide (**9g**)

The title compound was synthesized following the method described in general procedure 1, using Fmoc-d-Phe-OH (78 mg, 0.20 mmol) for the loading phase; Fmoc-Ala-OH (62 mg, 0.20 mmol), Fmoc-His(Trt)-OH (124 mg, 0.20 mmol), and Fmoc-Val-OH (68 mg, 0.20 mmol) for three separate coupling phases in the stated sequence; **7** (34 mg, 0.10 mmol) for the capping phase to afford a colorless solid (3.12 mg, 3.94 μmol, 10%). HRMS (Bruker MicroTOF)(*m/z*): Calculated (M+H)^+^ for C_44_H_56_N_8_O_6_ = 793.4396; measured = 793.4412. Analytical HPLC (system 1): 98% purity; *t*_R_*=* 13.14 min.

#### (*S*)-6-Amino-*N*-((*S*)-1-(((*R*)-1-amino-1-oxo-3-phenylpropan-2-yl)amino)-1-oxopropan-2-yl)-2-((*S*)-2-(3-(4-(2-benzylphenoxy)piperidin-1-yl)propanamido)-3-methylbutanamido)hexanamide (**9h**)

The title compound was synthesized following the method described in general procedure 1, using Fmoc-d-Phe-OH (78 mg, 0.20 mmol) for the loading phase; Fmoc-Ala-OH (62 mg, 0.20 mmol), Fmoc-Lys(Boc)-OH (94 mg, 0.20 mmol), and Fmoc-Val-OH (68 mg, 0.20 mmol) for three separate coupling phases in the stated sequence; **7** (34 mg, 0.10 mmol) for the capping phase to afford a colorless solid (2.92 mg, 3.73 μmol, 9%). HRMS (Bruker MicroTOF)(*m/z*): Calculated (M+H)^+^ for C_44_H_61_N_7_O_6_ = 784.4756; measured = 787.4772. Analytical HPLC (system 1): 98% purity; *t*_R_*=* 12.97 min.

#### (*S*)-4-(((*S*)-1-(((*R*)-1-Amino-1-oxo-3-phenylpropan-2-yl)amino)-1-oxopropan-2-yl)amino)-3-((*S*)-2-(3-(4-(2-benzylphenoxy)piperidin-1-yl)propanamido)-3-methylbutanamido)-4-oxobutanoic Acid (**9i**)

The title compound was synthesized following the method described in general procedure 1, using Fmoc-d-Phe-OH (78 mg, 0.20 mmol) for the loading phase; Fmoc-Ala-OH (62 mg, 0.20 mmol), Fmoc-Asp(OtBu)-OH (83 mg, 0.20 mmol), and Fmoc-Val-OH (68 mg, 0.20 mmol) for three separate coupling phases in the stated sequence; **7** (34 mg, 0.10 mmol) for the capping phase to afford a colorless solid (3.71 mg, 4.82 μmol, 12%). HRMS (Bruker MicroTOF)(*m/z*): Calculated (M+H)^+^ for C_42_H_54_N_6_O_8_ = 771.4076; measured = 771.4082. Analytical HPLC (system 1): 98% purity; *t*_R_*=* 14.60 min.

#### (*S*)-*N*-((*S*)-1-((2-(((*R*)-1-Amino-1-oxo-3-phenylpropan-2-yl)amino)-2-oxoethyl)amino)-3-hydroxy-1-oxopropan-2-yl)-2-(3-(4-(2-benzylphenoxy)piperidin-1-yl)propanamido)-3-methylbutanamide (**10a**)

The title compound was synthesized following the method described in general procedure 1, using Fmoc-d-Phe-OH (78 mg, 0.20 mmol) for the loading phase; Fmoc-Gly-OH (60 mg, 0.20 mmol), Fmoc-Ser(tBu)-OH (77 mg, 0.20 mmol), and Fmoc-Val-OH (68 mg, 0.20 mmol) for three separate coupling phases in the stated sequence; **7** (34 mg, 0.10 mmol) for the capping phase to afford a colorless solid (2.67 mg, 3.67 μmol, 9%). HRMS (Bruker MicroTOF)(*m/z*): Calculated (M+H)^+^ for C_40_H_52_N_6_O_7_ = 729.3970; measured = 729.3984. Analytical HPLC (system 1): 97% purity; *t*_R_*=* 14.23 min.

#### (*S*)-*N*-((*R*)-1-Amino-1-oxo-3-phenylpropan-2-yl)-2-((*S*)-2-((*S*)-2-(3-(4-(2-benzylphenoxy)piperidin-1-yl)propanamido)-3-methylbutanamido)-3-hydroxypropanamido)-3-methylbutanamide (**10b**)

The title compound was synthesized following the method described in general procedure 1, using Fmoc-d-Phe-OH (78 mg, 0.20 mmol) for the loading phase; Fmoc-Val-OH (68 mg, 0.20 mmol), Fmoc-Ser(tBu)-OH (77 mg, 0.20 mmol), and Fmoc-Val-OH (68 mg, 0.20 mmol) for three separate coupling phases in the stated sequence; **7** (34 mg, 0.10 mmol) for the capping phase to afford a colorless solid (1.58 mg, 2.05 μmol, 5%). HRMS (Bruker MicroTOF)(*m/z*): Calculated (M+H)^+^ for C_43_H_58_N_6_O_7_ = 771.4440; measured = 771.4452. Analytical HPLC (system 1): 98% purity; *t*_R_*=* 15.14 min.

#### (*S*)-*N*-((*S*)-1-(((*S*)-1-(((*R*)-1-Amino-1-oxo-3-phenylpropan-2-yl)amino)-1-oxo-3-phenylpropan-2-yl)amino)-3-hydroxy-1-oxopropan-2-yl)-2-(3-(4-(2-benzylphenoxy)piperidin-1-yl)propanamido)-3-methylbutanamide (**10c**)

The title compound was synthesized following the method described in general procedure 1, using Fmoc-d-Phe-OH (78 mg, 0.20 mmol) for the loading phase; Fmoc-l-Phe-OH (78 mg, 0.20 mmol), Fmoc-Ser(tBu)-OH (77 mg, 0.20 mmol), and Fmoc-Val-OH (68 mg, 0.20 mmol) for three separate coupling phases in the stated sequence; **7** (34 mg, 0.10 mmol) for the capping phase to afford a colorless solid (2.35 mg, 2.87 μmol, 7%). HRMS (Bruker MicroTOF)(*m/z*): Calculated (M+H)^+^ for C_47_H_58_N_6_O_7_ = 819.4440; measured = 819.4474. Analytical HPLC (system 1): 98% purity; *t*_R_*=* 15.85 min.

#### (*S*)-*N*-((*S*)-1-(((*S*)-1-(((*R*)-1-Amino-1-oxo-3-phenylpropan-2-yl)amino)-3-(4-hydroxyphenyl)-1-oxopropan-2-yl)amino)-3-hydroxy-1-oxopropan-2-yl)-2-(3-(4-(2-benzylphenoxy)piperidin-1-yl)propanamido)-3-methylbutanamide (**10d**)

The title compound was synthesized following the method described in general procedure 1, using Fmoc-d-Phe-OH (78 mg, 0.20 mmol) for the loading phase; Fmoc-Tyr(tBu)-OH (92 mg, 0.20 mmol), Fmoc-Ser(tBu)-OH (77 mg, 0.20 mmol), and Fmoc-Val-OH (68 mg, 0.20 mmol) for three separate coupling phases in the stated sequence; **7** (34 mg, 0.10 mmol) for the capping phase to afford a colorless solid (2.31 mg, 2.77 μmol, 7%). HRMS (Bruker MicroTOF)(*m/z*): Calculated (M+H)^+^ for C_47_H_58_N_6_O_8_ = 835.4389; measured = 835.4398. Analytical HPLC (system 1): 98% purity; *t*_R_*=* 14.78 min.

#### (*S*)-*N*-((*S*)-1-(((*S*)-1-(((*R*)-1-Amino-1-oxo-3-phenylpropan-2-yl)amino)-3-hydroxy-1-oxopropan-2-yl)amino)-3-hydroxy-1-oxopropan-2-yl)-2-(3-(4-(2-benzylphenoxy)piperidin-1-yl)propanamido)-3-methylbutanamide (**10e**)

The title compound was synthesized following the method described in general procedure 1, using Fmoc-d-Phe-OH (78 mg, 0.20 mmol) for the loading phase; Fmoc-Ser(tBu)-OH (77 mg, 0.20 mmol), Fmoc-Ser(tBu)-OH (77 mg, 0.20 mmol), and Fmoc-Val-OH (68 mg, 0.20 mmol) for three separate coupling phases in the stated sequence; **7** (34 mg, 0.10 mmol) for the capping phase to afford a colorless solid (0.39 mg, 0.51 μmol, 1%). HRMS (Bruker MicroTOF)(*m/z*): Calculated (M+H)^+^ for C_41_H_54_N_6_O_8_ = 759.4076; measured = 759.4088. Analytical HPLC (system 1): 88% purity; *t*_R_*=* 14.03 min.

#### (*S*)-*N*1-((*R*)-1-Amino-1-oxo-3-phenylpropan-2-yl)-2-((*S*)-2-((*S*)-2-(3-(4-(2-benzylphenoxy)piperidin-1-yl)propanamido)-3-methylbutanamido)-3-hydroxypropanamido)succinamide (**10f**)

The title compound was synthesized following the method described in general procedure 1, using Fmoc-d-Phe-OH (78 mg, 0.20 mmol) for the loading phase; Fmoc-Asn(Trt)-OH (120 mg, 0.20 mmol), Fmoc-Ser(tBu)-OH (77 mg, 0.20 mmol), and Fmoc-Val-OH (68 mg, 0.20 mmol) for three separate coupling phases in the stated sequence; **7** (34 mg, 0.10 mmol) for the capping phase to afford a colorless solid (1.90 mg, 2.42 μmol, 6%). HRMS (Bruker MicroTOF)(*m/z*): Calculated (M+H)^+^ for C_42_H_55_N_7_O_8_ = 786.4185; measured = 786.4192. Analytical HPLC (system 1): 98% purity; *t*_R_*=* 13.90 min.

#### (*S*)-*N*-((*S*)-1-(((*S*)-1-(((*R*)-1-Amino-1-oxo-3-phenylpropan-2-yl)amino)-3-(1*H*-imidazol-4-yl)-1-oxopropan-2-yl)amino)-3-hydroxy-1-oxopropan-2-yl)-2-(3-(4-(2-benzylphenoxy)piperidin-1-yl)propanamido)-3-methylbutanamide (**10g**)

The title compound was synthesized following the method described in general procedure 1, using Fmoc-d-Phe-OH (78 mg, 0.20 mmol) for the loading phase; Fmoc-Ser(tBu)-OH (77 mg, 0.20 mmol), Fmoc-His(Trt)-OH (124 mg, 0.20 mmol), and Fmoc-Val-OH (68 mg, 0.20 mmol) for three separate coupling phases in the stated sequence; **7** (34 mg, 0.10 mmol) for the capping phase to afford a colorless solid (3.03 mg, 3.75 μmol, 9%). HRMS (Bruker MicroTOF)(*m/z*): Calculated (M+H)^+^ for C_44_H_56_N_8_O_7_ = 809.4345; measured = 809.4345. Analytical HPLC (system 1): 98% purity; *t*_R_*=* 12.67 min.

#### (*S*)-6-Amino-*N*-((*R*)-1-amino-1-oxo-3-phenylpropan-2-yl)-2-((*S*)-2-((*S*)-2-(3-(4-(2-benzylphenoxy)piperidin-1-yl)propanamido)-3-methylbutanamido)-3-hydroxypropanamido)hexanamide (**10h**)

The title compound was synthesized following the method described in general procedure 1, using Fmoc-d-Phe-OH (78 mg, 0.20 mmol) for the loading phase; Fmoc-Lys(Boc)-OH (94 mg, 0.20 mmol), Fmoc-Ser(tBu)-OH (77 mg, 0.20 mmol), and Fmoc-Val-OH (68 mg, 0.20 mmol) for three separate coupling phases in the stated sequence; **7** (34 mg, 0.10 mmol) for the capping phase to afford a colorless solid (2.79 mg, 3.49 μmol, 9%). HRMS (Bruker MicroTOF)(*m/z*): Calculated (M+H)^+^ for C_44_H_61_N_7_O_7_ = 800.4705; measured = 800.4681. Analytical HPLC (system 1): 98% purity; *t*_R_*=* 12.52 min.

#### (*S*)-4-(((*R*)-1-Amino-1-oxo-3-phenylpropan-2-yl)amino)-3-((*S*)-2-((*S*)-2-(3-(4-(2-benzylphenoxy)piperidin-1-yl)propanamido)-3-methylbutanamido)-3-hydroxypropanamido)-4-oxobutanoic Acid (**10i**)

The title compound was synthesized following the method described in general procedure 1, using Fmoc-d-Phe-OH (78 mg, 0.20 mmol) for the loading phase; Fmoc-Asp(OtBu)-OH (83 mg, 0.20 mmol), Fmoc-Ser(tBu)-OH (77 mg, 0.20 mmol), and Fmoc-Val-OH (68 mg, 0.20 mmol) for three separate coupling phases in the stated sequence; **7** (34 mg, 0.10 mmol) for the capping phase to afford a colorless solid (1.98 mg, 2.52 μmol, 6%). HRMS (Bruker MicroTOF)(*m/z*): Calculated (M+H)^+^ for C_42_H_54_N_6_O_9_ = 787.4025; measured = 787.4044. Analytical HPLC (system 1): 98% purity; *t*_R_*=* 14.23 min.

#### (*S*)-*N*-((*S*)-1-(((*S*)-1-(((*S*)-1-Amino-1-oxo-3-phenylpropan-2-yl)amino)-3-(4-hydroxyphenyl)-1-oxopropan-2-yl)amino)-3-hydroxy-1-oxopropan-2-yl)-2-(3-(4-(2-benzylphenoxy)piperidin-1-yl)propanamido)-3-methylbutanamide (**11a**)

The title compound was synthesized following the method described in general procedure 1, using Fmoc-l-Phe-OH (78 mg, 0.20 mmol) for the loading phase; Fmoc-Tyr(tBu)-OH (92 mg, 0.20 mmol), Fmoc-Ser(tBu)-OH (77 mg, 0.20 mmol), and Fmoc-Val-OH (68 mg, 0.20 mmol) for three separate coupling phases in the stated sequence; **7** (34 mg, 0.10 mmol) for the capping phase to afford a colorless solid (0.81 mg, 0.97 μmol, 2%). HRMS (Bruker MicroTOF)(*m/z*): Calculated (M+H)^+^ for C_47_H_58_N_6_O_8_ = 835.4389; measured = 835.4369. Analytical HPLC (system 1): 95% purity; *t*_R_ = 15.15 min.

#### (*S*)-*N1*-((*S*)-1-Amino-1-oxo-3-phenylpropan-2-yl)-2-((*S*)-2-((*S*)-2-(3-(4-(2-benzylphenoxy)piperidin-1-yl)propanamido)-3-methylbutanamido)-3-hydroxypropanamido)succinimide (**11b**)

The title compound was synthesized following the method described in general procedure 1, using Fmoc-l-Phe-OH (78 mg, 0.20 mmol) for the loading phase; Fmoc-Asn(Trt)-OH (120 mg, 0.20 mmol), Fmoc-Ser(tBu)-OH (77 mg, 0.20 mmol), and Fmoc-Val-OH (68 mg, 0.20 mmol) for three separate coupling phases in the stated sequence; **7** (34 mg, 0.10 mmol) for the capping phase to afford a colorless solid (1.07 mg, 1.36 μmol, 3%). HRMS (Bruker MicroTOF)(*m/z*): Calculated (M+H)^+^ for C_42_H_55_N_7_O_8_ = 786.4185; measured = 785.4197. Analytical HPLC (system 1): 98% purity; *t*_R_ = 14.17 min.

#### *tert*-Butyl 2-(Dimethoxymethyl)-1*H*-pyrrole-1-carboxylate (**13**)

2-Formylpyrrole (1.0 g, 10.5 mmol) was dissolved in DCM (20 mL) and treated with di-*tert*-butyl decarbonate (2.53 g, 11.6 mmol), triethylamine (1.70 mL, 11.6 mmol), and 4-dimethylaminopyridine (64 mg, 0.53 mmol). The reaction mixture was stirred at rt for 30 min. The reaction mixture was filtered through a silica plug with DCM washing and monitored by TLC. The filtrate was concentrated over a rotary evaporator to afford a pale brown oil (2.04 g, 10.4 mmol, 99%). The oil (1.0 g, 5.12 mmol) was dissolved in MeOH (5 mL) and treated with trimethyl orthoformate (1.10 mL, 10.3 mmol) and *p*-toluenesulfonic acid (20 mg, 0.103 mmol). The reaction mixture was stirred at rt for 3 h. The reaction mixture was diluted with saturated aqueous NaHCO_3_ (10 mL) and water (10 mL) and extracted once with EtOAc, the organic layer was washed with saturated brine, dried over MgSO_4_, and filtered, and the filtrate was concentrated over a rotary evaporator to afford a dark brown gum (1.07 g, 4.46 mmol, 87%). ^1^H NMR (400 MHz, CDCl_3_) δ 7.20 (dd, *J* = 3.3, 1.9 Hz, 1H, Pyrrole C5**H**), 6.45–6.39 (m, 1H, pyrrole C3**H**), 6.12–6.08 (m, 1H, pyrrole C4**H**), 5.88 (s, 1H, −C**H**(OMe)_2_), 3.32 (s, 6H, 2 × −OC**H_3_**), 1.58 (s, 9H, −OC(C**H_3_**)_3_). ^13^C NMR (101 MHz, CDCl_3_) δ 148.9, 131.4, 122.5, 113.4, 109.8, 98.4, 83.9, 53.4, 28.0.

#### (5-Formyl-1*H*-pyrrol-2-yl)boronic Acid (**14**)

*tert*-Butyl 2-(dimethoxymethyl)-1*H*-pyrrole-1-carboxylate (**13**) (1.20 g, 5.0 mmol) and triisopropylborate (1.80 mL, 7.80 mmol) were dissolved in dry THF (6 mL) and cooled in an ice bath under N_2_. The reaction mixture was treated dropwise with 2 M lithium diisopropylamide solution in THF/heptane/ethylbenzene (6.5 mL, 13.0 mmol) over a 15 min period and left to stir for 30 min in an ice bath. The reaction mixture was quenched with saturated NH_4_Cl, poured into a stirred aq. solution of 10% NaHSO_4_ (25 mL), and stirred at 70 °C for 2 h. The pH of the mixture was adjusted to 2 by gradual addition of solid NaHSO_4_, and the reaction mixture was stirred at 70 °C for 3 h. The reaction mixture was allowed to cool to rt and extracted three times with EtOAc. The combined organic layer was washed with saturated brine, dried over MgSO_4_, and filtered, and the filtrate concentrated over a rotary evaporator to afford a black gum. The resulting gum was triturated with 50% diisopropyl ether/hexane (10 mL), and subsequent filtration afforded a black solid (235 mg, 1.69 mmol, 34%). ^1^H NMR (400 MHz, DMSO-*d*_6_) δ 11.67 (s, 1H, pyrrole-N1**H**), 9.56 (s, 1H, −C**H**O), 8.18 (s, 2H, −B(O**H**)_2_), 6.96 (dd, *J* = 3.7, 2.2 Hz, 1H, pyrrole-C4**H**), 6.75 (dd, *J* = 3.7, 2.2 Hz, 1H, pyrrole-C3**H**). ^13^C NMR (101 MHz, DMSO-*d*_6_) δ 180.1, 135.5, 120.0, 119.3 (N.B. C2 carbon not observed due to quadrupolar boron). LC-MS *m/z* calculated (M+H)^+^ for C_5_H_6_BNO_3_ = 139.0, not found (*m/z* < 150); *t*_R_*=* 2.43 min.

#### 5-(Thiophen-2-yl)-1*H*-pyrrole-2-carbaldehyde (**15**)

(5-Formyl-1*H*-pyrrol-2-yl)boronic acid (**14**) (235 mg, 1.69 mmol) and 2-bromothiophene (140 μL, 1.44 mmol) were dissolved in 10% deionized water/dioxane (15 mL), and the reaction mixture was degassed by N_2_ sparging for 15 min. The reaction mixture was treated with Na_2_CO_3_ 178 mg, 1.69 mmol) and Pd(PPh_3_)_2_Cl_2_ (50 mg, 0.07 mmol). The reaction mixture was stirred at 100 °C for 3 h under N_2_. The reaction mixture was allowed to cool to rt, diluted with water (15 mL), and extracted three times with diethyl ether. The combined organic layer was washed with saturated brine, dried over MgSO_4_, and filtered, and the filtrate was concentrated over a rotary evaporator and purified by silica gel column chromatography (15% EtOAc/hexane) to afford a yellow-orange solid (82 mg, 0.46 mmol, 32%). ^1^H NMR (400 MHz, CDCl_3_) δ 9.96 (s, 1H, pyrrole-N1**H**), 9.49 (s, 1H, −C**H**O), 7.38 (d, *J* = 3.6 Hz, 1H, thiophene-C3**H**), 7.32 (d, *J* = 5.1 Hz, 1H, thiophene-C5**H**), 7.11–7.06 (m, 1H, thiophene-C4**H**), 7.01–6.97 (m, 1H, pyrrole-C4**H**), 6.55–6.50 (m, 1H, pyrrole-C3**H**). ^13^C NMR (101 MHz, CDCl_3_) δ 178.8, 134.9, 133.9, 133.0, 128.3, 125.9, 124.5, 123.1, 109.5. LC-MS *m/z* calculated (M+H)^+^ for C_9_H_7_NOS = 178.0, found = 178.1; *t*_R_*=* 2.61 min.

#### Tributyl(4-methoxybenzyl)phosphonium Chloride (**17**)

4-Methoxybenzyl chloride (**16**) (2.0 mL, 14.8 mmol) and tributylphosphine (6.0 mL, 22.5 mmol) were dissolved in dry toluene (15 mL), and the reaction mixture was heated under reflux for 18 h under N_2_. The reaction mixture was left to cool to rt and concentrated under N_2_ flow. The reaction mixture was subsequently triturated with diethyl ether and filtered, and the solids were dried in a vacuum oven to afford a colorless hygroscopic solid (4.62 g, 12.9 mmol, 86%). ^1^H NMR (400 MHz, DMSO-*d*_6_) δ 7.28 (dd, *J* = 8.8, 2.4 Hz, 2H, Ph-C2**H**), 6.98 (d, *J* = 8.5 Hz, 2H, Ph-C3**H**), 3.79 (d, *J* = 14.9 Hz, 2H, −C**H_2_**P−), 3.75 (s, 3H, −OC**H_3_**), 2.26–2.03 (m, 6H, 3 x -PC**H_2_**CH_2_CH_2_CH_3_), 1.44–1.33 (m, 12H, 3 × −PCH_2_C**H_2_**C**H_2_**CH_3_), 0.91–0.86 (m, 9H, 3 x -PCH_2_CH_2_CH_2_C**H_3_**). ^13^C NMR (101 MHz, DMSO-*d*_6_) δ 158.9 (d, *J* = 3.4 Hz), 131.1 (d, *J* = 4.8 Hz), 120.6 (d, *J* = 8.5 Hz), 114.8 (d, *J* = 2.7 Hz), 55.2, 24.5 (d, *J* = 44.6 Hz), 23.4 (d, *J* = 15.6 Hz), 22.5 (d, *J* = 4.6 Hz), 17.3 (d, *J* = 47.0 Hz), 13.2. LC-MS *m/z* calculated (M-Cl)^+^ for C_20_H_36_OP^+^ = 324.3, found = 324.4; *t*_R_*=* 2.39 min.

#### (*E*)-2-(4-Methoxystyryl)-1*H*-pyrrole (**18**)

Tributyl(4-methoxybenzyl)phosphonium chloride (**17**) (1.0 g, 2.79 mmol) was treated with NaOH (112 mg, 2.79 mmol) dissolved in deionized water (1 mL), and the reaction mixture was vortexed for 1 min and sonicated for 3 min to afford a white suspension. The reaction mixture was treated with 2-formylpyrrole (239 mg, 2.51 mmol) and heated in the MW to 100 °C for 25 min. The reaction mixture was diluted with water and extracted three times with DCM, and the combined organic layer was washed with saturated brine, dried over MgSO_4_, and filtered. The filtrate was washed through a silica plug with DCM and monitored by TLC. The resulting filtrate was concentrated over a rotary evaporator to afford a blue-green solid (380 mg, 1.91 mmol, 76%). ^1^H NMR (400 MHz, CDCl_3_) δ 8.30 (s, 1H), 7.41 – 7.34 (m, 2H, 2 × Ph-C2**H**), 6.90–6.87 (m, 2H, 2 × Ph-C3**H**), 6.84 (d, *J* = 16.6 Hz, 1H, −CHC**H**Ph), 6.82–6.76 (m, 1H, pyrrole-C5**H**), 6.63 (d, *J* = 16.5 Hz, 1H, −C**H**CHPh), 6.33–6.30 (m, 1H, pyrrole-C3**H**), 6.28–6.21 (m, 1H, pyrrole-C4**H**), 3.82 (s, 3H, −OC**H_3_**). ^13^C NMR (101 MHz, CDCl_3_) δ 159.0, 131.2, 130.5, 127.1, 123.3, 118.8, 117.3, 114.3, 110.0, 108.5, 55.5. LC-MS *m/z* calculated (M+H)^+^ for C_13_H_13_NO = 200.1, found = 200.0; *t*_R_*=* 2.93 min.

#### (*E*)-2-(4-(Prop-2-yn-1-yloxy)styryl)-1*H*-pyrrole (**19**)

(*E*)-2-(4-Methoxystyryl)-1*H*-pyrrole (**18**) (200 mg, 1.0 mmol) was dissolved in dry DMF (6 mL), and the reaction mixture was degassed by N_2_ sparging for 15 min. The reaction mixture was treated with sodium ethanethiolate (168 mg, 2.0 mmol), and the reaction mixture was stirred at 145 °C for 18 hr under N_2_. The reaction mixture was diluted with EtOAc (10 mL) and washed once with 0.5 M NH_4_Cl followed by saturated brine, dried over MgSO_4_, and filtered, and the filtrate was concentrated over a rotary evaporator to afford a black solid. The solid was dissolved in acetonitrile (5 mL) and treated with K_2_CO_3_ (208 mg, 1.51 mmol), and the reaction mixture was heated to reflux under N_2_ for 30 min. The reaction mixture was left to cool at rt, subsequently treated with propargyl bromide (100 μL, 1.10 mmol) and heated under reflux for 18 h. The reaction mixture was left to cool at rt and filtered. The precipitate was washed three times with acetonitrile, and the resulting filtrate was concentrated over a rotary evaporator to afford a gray solid (197 mg, 0.88 mmol, 88%). ^1^H NMR (400 MHz, CDCl_3_) δ 8.32 (s, 1H, Pyrrole **H**), 7.40–7.33 (m, 2H, 2 × Ph-C2**H**), 6.99 – 6.91 (m, 2H, 2 × Ph-C3**H**), 6.85 (d, *J* = 16.5 Hz, 1H, −CHC**H**Ph), 6.81–6.77 (m, 1H, pyrrole-C5**H**), 6.62 (d, *J* = 16.5 Hz, 1H, −C**H**CHPh), 6.36–6.30 (m, 1H, pyrrole-C3**H**), 6.28–6.21 (m, 1H, pyrrole-C4**H**), 4.70 (d, *J* = 2.4 Hz, 2H, OC**H_2_**C≡CH), 2.53 (t, *J* = 2.4 Hz, 1H, −OCH_2_C≡C**H**). ^13^C NMR (101 MHz, CDCl_3_) δ 156.8, 131.3, 131.0, 127.1, 123.0, 119.0, 117.7, 115.3, 110.0, 108.6, 78.7, 75.7, 56.0. LC-MS: *m/z* calculated (M+H)^+^ for C_15_H_13_NO = 224.1, found = 223.8; *t*_R_*=* 2.94 min.

#### (*E*)-5,5-Difluoro-7-(4-(prop-2-yn-1-yloxy)styryl)-3-(thiophen-2-yl)-5*H*-5λ^4^-dipyrrolo[1,2-*c*:2’,1’-*f*][1,3,2]diazaborinin-4-ium (**20**)

5-(Thiophen-2-yl)-1*H*-pyrrole-2-carbaldehyde (**15**) (151 mg, 0.85 mmol) and (*E*)-2-(4-(prop-2-yn-1-yloxy)styryl)-1*H*-pyrrole (**19**) (190 mg, 0.85 mmol) were dissolved in 1:10 dry MeOH/DCM (33 mL), and the reaction mixture was degassed by N_2_ sparging for 15 min. The reaction mixture was treated dropwise with POCl_3_ (80 μL, 0.85 mmol), and the reaction mixture stirred at rt for 15 h in the dark under N_2_. The reaction mixture was concentrated by N_2_ flow and diluted with DCM (150 mL). The reaction mixture was treated with DIPEA (1.40 mL, 10.2 mmol) and boron trifluoride diethyl etherate (1.25 mL, 10.2 mmol). The reaction mixture was stirred at rt for 2 h in the dark under N_2_. The reaction mixture was subsequently washed once with saturated brine, dried over MgSO_4_, and filtered, and the filtrate was concentrated over a rotary evaporator and purified by silica gel column chromatography (20% EtOAc/hexane) to afford a red iridescent solid (251 mg, 0.58 mmol, 69%). ^1^H NMR (400 MHz, DMSO-*d*_6_) δ 8.05 (dd, *J* = 3.8, 1.1 Hz, 1H, thiophene-C**H**), 7.84 (dd, *J* = 5.0, 1.0 Hz, 1H, thiophene-C**H**), 7.75 (d, *J* = 16.3 Hz, 1H, −CHC**H**Ph), 7.65–7.59 (m, 3H, 3 × Ar-C**H**), 7.41 (d, *J* = 16.2 Hz, 1H, -C**H**CHPh), 7.38 (d, *J* = 4.7 Hz, 1H, Ar-C**H**), 7.33–7.26 (m, 3H, 3 × Ar-C**H**), 7.12 (d, *J* = 8.8 Hz, 2H, Ar-C**H**), 6.96 (d, *J* = 4.3 Hz, 1H, Ar-C**H**), 4.88 (d, *J* = 2.4 Hz, 2H, −OC**H_2_**C≡CH), 3.63 (t, *J* = 2.4 Hz, 1H, −OCH_2_C≡C**H**). ^13^C NMR (101 MHz, DMSO) δ 158.6, 156.4, 147.7, 138.7, 136.5, 136.4, 133.7, 131.3, 130.3, 129.7, 129.2, 129.0, 128.9, 125.1, 119.4, 118.2, 116.08, 116.06, 115.7, 78.9, 78.6, 55.6. HRMS (Bruker MicroTOF)(*m/z*): Calculated (M+H)^+^ for C_24_H_17_B_1_F_2_N_2_O_1_S_1_ = 431.1195; measured = 431.1206. LC-MS: *m/z* calculated (M+H)^+^ for C_24_H_17_BF_2_N_2_OS = 430.1, found = 430.8; *t*_R_*=* 3.23 min.

#### (*S*)-*N*1-((*S*)-1-Amino-3-azido-1-oxopropan-2-yl)-2-((*S*)-2-((*S*)-2-(3-(4-(2-benzylphenoxy)piperidin-1-yl)propanamido)-3-methylbutanamido)-3-hydroxypropanamido)succinamide (**21a**)

The title compound was synthesized following the method described in general procedure 1, using Fmoc-ß-azido-Ala-OH (71 mg, 0.20 mmol) for the loading phase; Fmoc-Asn(Trt)-OH (120 mg, 0.20 mmol), Fmoc-Ser(tBu)-OH (77 mg, 0.20 mmol), and Fmoc-Val-OH (68 mg, 0.20 mmol) for three separate coupling phases in the stated sequence; **7** (34 mg, 0.10 mmol) for the capping phase to afford a colorless solid (0.5 mg, 0.67 μmol, 2%). HRMS (Bruker MicroTOF)(*m/z*): Calculated (M+H)^+^ for C_36_H_50_N_10_O_8_ = 751.3886; measured = 751.3859.

#### (*S*)-*N*-((S)-1-Amino-3-azido-1-oxopropan-2-yl)-2-((S)-2-((S)-2-(3-(4-(2-benzylphenoxy)piperidin-1-yl)propanamido)propanamido)propanamido)propanamide (**21b**)

The title compound was synthesized following the method described in general procedure 1, using Fmoc-ß-azido-Ala-OH (71 mg, 0.20 mmol) for the loading phase; Fmoc-Ala-OH (63 mg, 0.20 mmol), Fmoc-Ala-OH (63 mg, 0.20 mmol), and Fmoc-Ala-OH (63 mg, 0.20 mmol) for three separate coupling phases in the stated sequence; **7** (34 mg, 0.10 mmol) for the capping phase to afford a colorless solid (0.6 mg, 0.90 μmol, 2%). HRMS (Bruker MicroTOF)(*m/z*): Calculated (M+H)^+^ for C_33_H_45_N_9_O_6_ = 664.3566; measured = 664.3578.

#### (*S*)-5-(((*S*)-4-Amino-1-(((*S*)-1-amino-3-azido-1-oxopropan-2-yl)amino)-1,4-dioxobutan-2-yl)amino)-4-((*S*)-2-(3-(4-(2-benzylphenoxy)piperidin-1-yl)propanamido)-3-methylbutanamido)-5-oxopentanoic Acid (**21c**)

The title compound was synthesized following the method described in general procedure 1, using Fmoc-ß-azido-Ala-OH (71 mg, 0.20 mmol) for the loading phase; Fmoc-Glu(OtBu)-OH (85 mg, 0.20 mmol), Fmoc-Ser(tBu)-OH (77 mg, 0.20 mmol), and Fmoc-Val-OH (68 mg, 0.20 mmol) for three separate coupling phases in the stated sequence; **7** (34 mg, 0.10 mmol) for the capping phase to afford a colorless solid (0.5 mg, 0.61 μmol, 2%). HRMS (Bruker MicroTOF)(*m/z*): Calculated (M+H)+ for C38H52N10O9 = 815.3811; measured = 815.3770.

#### (*S*)-*N*1-((*S*)-1-Amino-3-(4-((4-((*E*)-2-(5,5-difluoro-7-(thiophen-2-yl)-5*H*-5λ^4^,6λ^4^-dipyrrolo[1,2-*c*:2’,1’-*f*][1,3,2]diazaborinin-3-yl)vinyl)phenoxy)methyl)-1*H*-1,2,3-triazol-1-yl)-1-oxopropan-2-yl)-2-((*S*)-2-((*S*)-2-(3-(4-(2-benzylphenoxy)piperidin-1-yl)propanamido)-3-methylbutanamido)-3-hydroxypropanamido)succinamide (**22a**)

The title compound was synthesized following the method described in general procedure 2, using **21a** (0.5 mg, 0.67 μmol) to afford a blue solid (0.53 mg, 0.45 μmol, 67%). HRMS (Bruker MicroTOF)(*m/z*): Calculated (M+H)^+^ for C_60_H_67_B_1_F_2_N_12_O_9_S_1_ = 1181.5009; Measured = 1181.4954; Error = 5.5ppm. Analytical HPLC (system 1): 96% purity; *t*_R_ = 15.90 min.

#### (*S*)-*N*-((*S*)-1-Amino-3-(4-((4-((*E*)-2-(5,5-difluoro-7-(thiophen-2-yl)-5*H*-5λ^4^,6λ^4^-dipyrrolo[1,2-*c*:2’,1’-*f*][1,3,2]diazaborinin-3-yl)vinyl)phenoxy)methyl)-1*H*-1,2,3-triazol-1-yl)-1-oxopropan-2-yl)-2-((*S*)-2-((*S*)-2-(3-(4-(2-benzylphenoxy)piperidin-1-yl)propanamido)propanamido)propanamido)propenamide (**22b**)

The title compound was synthesized following the method described in general procedure 2, using **21b** (0.6 mg, 0.90 μmol) to afford a blue solid (0.77 mg, 0.70 μmol, 79%). HRMS (Bruker MicroTOF)(*m/z*): Calculated (M+H)^+^ for C_57_H_62_B_1_F_2_N_11_O_7_S_1_ = 1094.4688; measured = 1094.4711. Analytical HPLC (system 1): 98% purity; *t*_R_ = 15.85 min.

#### (*S*)-5-(((*S*)-4-Amino-1-(((*S*)-1-amino-3-(4-((4-((*E*)-2-(5,5-difluoro-7-(thiophen-2-yl)-5*H*-5λ^4^,6λ^4^-dipyrrolo[1,2-*c*:2’,1’-*f*][1,3,2]diazaborinin-3-yl)vinyl)phenoxy)methyl)-1*H*-1,2,3-triazol-1-yl)-1-oxopropan-2-yl)amino)-1,4-dioxobutan-2-yl)amino)-4-((*S*)-2-(3-(4-(2-benzylphenoxy)piperidin-1-yl)propanamido)-3-methylbutanamido)-5-oxopentanoic Acid (**22c**)

The title compound was synthesized following the method described in general procedure 2, using **21c** (0.5 mg, 0.61 μmol) to afford a blue solid (0.45 mg, 0.37 μmol, 60%). HRMS (Bruker MicroTOF)(*m*/*z*): Calculated (M+H)+ for C_62_H_69_B_1_F_2_N_12_O_10_S_1_ = 1223.5114, measured = 1223.5051. Analytical HPLC (System 2): 95% purity; *t*_R_ = 16.21 min.

#### (*E*)-3-(dibenzo[*b*,*e*]oxepin-11(6*H*)-ylidene)-*N*-methylpropan-1-amine(desmethyldoxepin) (**24**)

Doxepin hydrochloride (**23**) (500 mg, 1.58 mmol) with a reported *E:Z* isomeric ratio of 85:15 (Tocris Bioscience) was dissolved in DCM (25 mL) and washed once with saturated NaHCO_3_ solution (25 mL). The resulting organic layer was washed with saturated brine, dried over MgSO_4_, and filtered, and the filtrate was concentrated over a rotary evaporator. The resulting gum was dissolved in chloroform (5 mL) and treated with trichloroethyl-chloroformate (240 μL, 1.74 mmol) and triethylamine (243 μL, 1.74 mmol). The reaction mixture was stirred at rt for 6 h. The reaction mixture was concentrated over a rotary evaporator and purified by silica gel column chromatography (20% EtOAc/hexane) to afford a yellow oil (536 mg), which was used in the next step of the reaction without further purification. The oil (491 mg, 1.11 mmol) was dissolved in THF (4 mL) under N_2_ and treated with 1 M NaH_2_PO_4_ (1.0 mL, 1.0 mmol) and zinc powder (1.00 g, 16.7 mmol). The reaction mixture was stirred at rt overnight. The reaction mixture was acidified with 2 M HCl to pH 1 and washed three times with DCM. The pH of the aqueous layer was adjusted to 14 by gradual addition of 2 M NaOH and extracted three times with DCM. The combined organic layer was washed once with saturated brine, dried over MgSO_4_, and filtered, and the filtrate was concentrated over a rotary evaporator to afford a pale-yellow oil (170 mg, 0.64 mmol, 45% over 2 steps). ^1^H NMR (400 MHz, CDCl_3_) δ 7.40–7.21 (m, 5H, Ar-C**H**), 7.16–7.09 (m, 1H, Ar-C**H**), 6.92–6.82 (m, 1H, Ar-C**H**), 6.76 (dd, *J* = 8.2, 1.3 Hz, 1H, Ar-C**H**), 6.02 (t, *J* = 7.4 Hz, 1H, −CC**H**CH_2_−), 5.54 (br s, 1H, PhOC**H_2_**Ph), 4.83 (br s, 1H, PhOC**H_2_**Ph), 2.71 (t, *J* = 7.1 Hz, 2H, −C**H_2_**CH_2_N-), 2.44–2.36 (m, 5H, −CH_2_C**H_2_**N– and −NC**H_3_**). ^13^C NMR (101 MHz, CDCl_3_) δ 155.2, 141.2, 140.9, 134.3, 130.2, 129.6, 129.2, 128.7, 128.3, 128.1, 127.9, 127.4, 121.1, 119.3, 70.2, 51.7, 36.2, 29.7. LC-MS *m/z* calculated (M+H)^+^ for C_18_H_19_NO = 266.2, found = 265.7; *t*_R_*=* 2.16 min.

#### Methyl (*E*)-3-((3-(Dibenzo[*b*,*e*]oxepin-11(6*H*)-ylidene)propyl)(methyl)amino)propanoate (**25**)

(*E*)-3-(Dibenzo[*b*,*e*]oxepin-11(6*H*)-ylidene)-*N*-methylpropan-1-amine (**24**) (161 mg, 0.61 mmol) was dissolved in DCE (5 mL) and treated with methyl acrylate (660 μL, 7.32 mmol) and DIPEA (1.0 mL, 5.75 mmol). The reaction mixture was stirred under reflux overnight. The reaction mixture was concentrated over a rotary evaporator and purified by silica gel column chromatography (20% EtOAc/hexane + 1% Et_3_N) to afford a pale-yellow oil (148 mg, 0.42 mmol, 69%). ^1^H NMR (400 MHz, CDCl_3_) δ 7.40–7.19 (m, 5H, Ar-C**H**), 7.15–7.06 (m, 1H, Ar-C**H**), 6.91–6.82 (m, 1H, Ar-C**H**), 6.75 (dd, *J* = 8.1, 1.3 Hz, 1H, Ar-C**H**), 6.01 (t, *J* = 7.4 Hz, 1H, −CC**H**CH_2_−), 5.54 (br s, 1H, PhOC**H_2_**Ph), 4.84 (br s, 1H, PhOC**H_2_**Ph), 3.62 (s, 3H, COOC**H_3_**), 2.66 (t, *J* = 7.2 Hz, 2H, −NC**H_2_**CH_2_CO−), 2.48 (t, *J* = 7.3 Hz, 2H, −CH_2_C**H_2_**N−), 2.43 (t, *J* = 7.2 Hz, 2H, −NCH_2_C**H_2_**CO−), 2.37–2.27 (m, 2H, −C**H_2_**CH_2_N−), 2.14 (s, 3H, −NC**H_3_**). ^13^C NMR (101 MHz, CDCl_3_) δ 173.1, 155.2, 141.4, 140.1, 134.4, 130.3, 130.2, 129.1, 128.6, 128.2, 128.0, 127.8, 127.5, 121.1, 119.2, 70.2, 57.1, 52.8, 51.7, 41.8, 32.5, 27.3. HRMS (Bruker MicroTOF)(*m/z*): Calculated (M+Na)^+^ for C_22_H_25_N_1_O_3_ = 374.1727; measured (M+Na)^+^ = 374.1740. LC-MS *m/z* calculated (M+H)^+^ for C_22_H_25_NO_3_= 352.2, found = 351.6; *t*_R_*=* 2.21 min.

#### (*E*)-3-((3-(Dibenzo[*b*,*e*]oxepin-11(6*H*)-ylidene)propyl)(methyl)amino)propanoic Acid (**26**)

Methyl (*E*)-3-((3-(dibenzo[*b*,*e*]oxepin-11(6*H*)-ylidene)propyl)(methyl)amino)-propanoate (**25**) (140 mg, 0.40 mmol) was dissolved in THF (2.5 mL) and treated dropwise with NaOH (56 mg, 1.40 mmol) dissolved in deionized water (5 mL). The reaction mixture was stirred at rt for 1 h. The reaction mixture was concentrated over a rotary evaporator, and the pH of the reaction mixture was adjusted to 6. The reaction mixture was diluted with deionized water (10 mL) and extracted four times with CHCl_3_. The combined organic layer was washed once with saturated brine, dried over MgSO_4_, and filtered, and the filtrate was concentrated over a rotary evaporator to afford a pale-yellow gum (130 mg, 0.39 mmol, 96%). NMR indicated an *E*:*Z* ratio of 79:21. ^1^H NMR (400 MHz, CD_3_OD) δ 7.46–7.36 (m, 3H, Ar-C**H**), 7.33–7.28 (m, 2H, Ar-C**H**), 7.15–7.09 (m, 1H, Ar-C**H**), 6.90–6.85 (m, 1H, Ar-C**H**), 6.71 (dd, *J* = 8.3, 1.3 Hz, 1H, Ar-C**H**), 6.01 (t, *J* = 7.4 Hz, 1H, −CC**H**CH_2_−), 5.55 (br s, 1H, PhOC**H_2_**Ph), 5.21 (br s, 1H, PhOC**H_2_**Ph), 3.20 (t, *J* = 7.4 Hz, 2H, −CH_2_C**H_2_**N−), 3.16 (t, *J* = 6.4 Hz, 2H, −NCH_2_C**H_2_**CO−) 2.67–2.60 (m, 2H, −C**H_2_**CH_2_N−), 2.65 (s, 3H, −NC**H_3_**) 2.48 (t, *J* = 6.4 Hz, 2H, −NC**H_2_**CH_2_CO−). ^13^C NMR (101 MHz, CD_3_OD) δ 177.7, 156.7, 144.6, 141.6, 136.0, 131.1, 130.5, 129.9, 129.5, 129.5, 128.6, 127.9, 126.1, 122.1, 120.2, 70.9, 56.2, 54.8, 39.7, 31.1, 25.8. HRMS (Bruker MicroTOF)(*m/z*): Calculated (M+H)^+^ for C_21_H_23_N_1_O_3_ = 338.1751; measured = 338.1758. LC-MS *m/z* calculated (M+H)^+^ for C_21_H_23_NO_3_ = 338.2, found = 337.6; *t*_R_*=* 2.23 min.

#### (*S*)-*N*1-(3-(((*S*)-1-Amino-5-azido-1-oxopentan-2-yl)amino)-3-oxopropyl)-2-((*S*)-2-((*S*)-2-(3-(4-(2-benzylphenoxy)piperidin-1-yl)propanamido)-3-methylbutanamido)-3-hydroxypropanamido)succinamide (**27a**)

The title compound was synthesized following the method described in general procedure 1, using Fmoc-Orn(N_3_)-OH (76 mg, 0.20 mmol) for the loading phase; Fmoc-ß-Ala-OH (63 mg, 0.20 mmol), Fmoc-Asn(Trt)-OH (120 mg, 0.20 mmol), Fmoc-Ser(tBu)-OH (77 mg, 0.20 mmol), and Fmoc-Val-OH (68 mg, 0.20 mmol) for four separate coupling phases in the stated sequence; **7** (34 mg, 0.10 mmol) for the capping phase to afford a colorless solid (1.8 mg, 2.12 μmol, 5%). HRMS (Bruker MicroTOF)(*m/z*): Calculated (M+H)^+^ for C_41_H_59_N_11_O_9_ = 850.4570; measured = 850.4596. Analytical HPLC (system 1): 91% purity; *t*_R_*=* 12.50 min.

#### (*S*)-5-Azido-2-((6*S*,9*S*,12*S*)-16-(4-(2-benzylphenoxy)piperidin-1-yl)-6,9,12-trimethyl-5,8,11,14-tetraoxo-4,7,10,13-tetraazahexadecanamido)pentanamide (**27b**)

The title compound was synthesized following the method described in general procedure 1, using Fmoc-Orn(N_3_)-OH (76 mg, 0.20 mmol) for the loading phase; Fmoc-ß-Ala-OH (63 mg, 0.20 mmol), Fmoc-Ala-OH (63 mg, 0.20 mmol), Fmoc-Ala-OH (63 mg, 0.20 mmol), and Fmoc-Ala-OH (63 mg, 0.20 mmol) for four separate coupling phases in the stated sequence; **7** (34 mg, 0.10 mmol) for the capping phase to afford a colorless solid (2.2 mg, 2.89 μmol, 7%). HRMS (Bruker MicroTOF)(*m/z*): Calculated (M+H)^+^ for C_38_H_54_N_10_O_7_ = 763.4250; measured = 763.4274. Analytical HPLC (system 1): 92% purity; *t*_R_*=* 12.39 min.

#### (*S*)-*N*1-(3-(((*S*)-1-Amino-5-azido-1-oxopentan-2-yl)amino)-3-oxopropyl)-2-((*S*)-2-((*S*)-2-(3-(((*E*)-3-(dibenzo[*b*,*e*]oxepin-11(6*H*)-ylidene)propyl)amino)propanamido)-3-methylbutanamido)-3-hydroxypropanamido)succinamide (**27c**)

The title compound was synthesized following the method described in general procedure 1, using Fmoc-Orn(N_3_)-OH (76 mg, 0.20 mmol) for the loading phase; Fmoc-ß-Ala-OH (63 mg, 0.20 mmol), Fmoc-Asn(Trt)-OH (120 mg, 0.20 mmol), Fmoc-Ser(tBu)-OH (77 mg, 0.20 mmol), and Fmoc-Val-OH (68 mg, 0.20 mmol) for four separate coupling phases in the stated sequence; **26** (34 mg, 0.10 mmol) for the capping phase to afford a colorless solid (3.3 mg, 3.89 μmol, 10%). HRMS (Bruker MicroTOF)(*m/z*): Calculated (M+H)^+^ for C_41_H_57_N_11_O_9_ = 848.4413; measured = 848.4459. Analytical HPLC (system 1): 92% purity; *t*_R_*=* 12.43 min.

#### (*S*)-5-Azido-2-((6*S*,9*S*,12*S,E*)-20-(dibenzo[*b*,*e*]oxepin-11(6*H*)-ylidene)-6,9,12-trimethyl-5,8,11,14-tetraoxo-4,7,10,13,17-pentaazaicosanamido)pentanamide (**27d**)

The title compound was synthesized following the method described in general procedure 1, using Fmoc-Orn(N_3_)-OH (76 mg, 0.20 mmol) for the loading phase; Fmoc-ß-Ala-OH (63 mg, 0.20 mmol), Fmoc-Ala-OH (63 mg, 0.20 mmol), Fmoc-Ala-OH (63 mg, 0.20 mmol), and Fmoc-Ala-OH (63 mg, 0.20 mmol) for four separate coupling phases in the stated sequence; **26** (34 mg, 0.10 mmol) for the capping phase to afford a colorless solid (3.4 mg, 4.47 μmol, 11%). HRMS (Bruker MicroTOF)(*m/z*): Calculated (M+H)^+^ for C_38_H_52_N_10_O_7_ = 761.4093; measured = 761.4097. Analytical HPLC (system 1): 94% purity; *t*_R_*=* 12.38 min.

#### (*S*)-*N*1-(3-(((*S*)-1-Amino-5-(4-((4-((*E*)-2-(5,5-difluoro-7-(thiophen-2-yl)-5*H*-5λ^4^,6λ^4^-dipyrrolo[1,2-*c*:2’,1’-*f*][1,3,2]diazaborinin-3-yl)vinyl)phenoxy)methyl)-1*H*-1,2,3-triazol-1-yl)-1-oxopentan-2-yl)amino)-3-oxopropyl)-2-((*S*)-2-((*S*)-2-(3-(4-(2-benzylphenoxy)piperidin-1-yl)propanamido)-3-methylbutanamido)-3-hydroxypropanamido)succinamide (**28a**)

The title compound was synthesized following the method described in general procedure 2, using **27a** (0.9 mg, 1.06 μmol) to afford a blue solid (0.73 mg, 0.57 μmol, 54%). HRMS (Bruker MicroTOF)(*m/z*): Calculated (M+H)^+^ for C_65_H_76_B_1_F_2_N_13_O_10_S_1_ = 1280.5693; measured = 1280.5718. Analytical HPLC (system 1): 98% purity; *t*_R_ = 15.92 min**.**

#### (*S*)-2-((6*S*,9*S*,12*S*)-16-(4-(2-Benzylphenoxy)piperidin-1-yl)-6,9,12-trimethyl-5,8,11,14-tetraoxo-4,7,10,13-tetraazahexadecanamido)-5-(4-((4-((*E*)-2-(5,5-difluoro-7-(thiophen-2-yl)-5*H*-5*λ*^4^,6*λ*^4^-dipyrrolo[1,2-*c*:2’,1’-*f*][1,3,2]diazaborinin-3-yl)vinyl)phenoxy)methyl)-1*H*-1,2,3-triazol-1-yl)pentanamide (**28b**)

The title compound was synthesized following the method described in general procedure 2, using **27b** (1.1 mg, 1.45 μmol) to afford a blue solid (0.78 mg, 0.65 μmol, 45%). HRMS (Bruker MicroTOF)(*m/z*): Calculated (M+H)^+^ for C_62_H_71_B_1_F_2_N_12_O_8_S_1_ = 1193.5372; measured = 1193.5443. Analytical HPLC (system 1): 98% purity; *t*_R_ = 15.95 min**.**

#### (*S*)-*N*1-(3-(((*S*)-1-Amino-5-(4-((4-((*E*)-2-(5,5-difluoro-7-(thiophen-2-yl)-5*H*-5λ^4^,6λ^4^-dipyrrolo[1,2-*c*:2’,1’-*f*][1,3,2]diazaborinin-3-yl)vinyl)phenoxy)methyl)-1*H*-1,2,3-triazol-1-yl)-1-oxopentan-2-yl)amino)-3-oxopropyl)-2-((*S*)-2-((*S*)-2-(3-(((*E*)-3-(dibenzo[*b*,*e*]oxepin-11(6*H*)-ylidene)propyl)(methyl)amino)propanamido)-3-methylbutanamido)-3-hydroxypropanamido)succinamide (28c)

The title compound was synthesized following the method described in general procedure 2, using **27c** (1.1 mg, 1.88 μmol) to afford a blue solid (0.79 mg, 0.62 μmol, 33%). HRMS (Bruker MicroTOF)(*m/z*): Calculated (M+H)^+^ for C_65_H_74_B_1_F_2_N_13_O_10_S_1_ = 1278.5536; measured = 1278.5584. Analytical HPLC (system 1): 98% purity; *t*_R_ = 16.35 min**.**

#### (*S*)-2-((6*S*,9*S*,12*S*,*E*)-20-(Dibenzo[*b*,*e*]oxepin-11(6*H*)-ylidene)-6,9,12-trimethyl-5,8,11,14-tetraoxo-4,7,10,13,17-pentaazaicosanamido)-5-(4-((4-((E)-2-(5,5-difluoro-7-(thiophen-2-yl)-5*H*-5λ^4^,6λ^*4*^-dipyrrolo[1,2-*c*:2’,1’-*f*][1,3,2]diazaborinin-3-yl)vinyl)phenoxy)methyl)-1*H*-1,2,3-triazol-1-yl)pentanamide (**28d**)

The title compound was synthesized following the method described in general procedure 2, using **27d** (1.7 mg, 2.24 μmol) to afford a blue solid (0.88 mg, 0.74 μmol, 33%). HRMS (Bruker MicroTOF)(*m/z*): Calculated (M+H)^+^ for C_62_H_69_B_1_F_2_N_12_O_8_S_1_ = 1191.5216; measured = 1191.5283. Analytical HPLC (system 1): 98% purity; *t*_R_ = 16.23 min.

#### (*S*)-2-((*S*)-2-((*S*)-2-(3-(4-(2-Benzylphenoxy)piperidin-1-yl)propanamido)-3-methylbutanamido)-3-hydroxypropanamido)-*N*1-(3-(((*S*)-1,6-diamino-1-oxohexan-2-yl)amino)-3-oxopropyl)succinamide (**29a**)

The title compound was synthesized following the method described in general procedure 1, using Fmoc-Lys(Boc)-OH (94 mg, 0.20 mmol) for the loading phase; Fmoc-ß-Ala-OH (63 mg, 0.20 mmol), Fmoc-Asn(Trt)-OH (120 mg, 0.20 mmol), Fmoc-Ser(tBu)-OH (77 mg, 0.20 mmol), and Fmoc-Val-OH (68 mg, 0.20 mmol) for four separate coupling phases in the stated sequence; **7** (34 mg, 0.10 mmol) for the capping phase to afford a colorless solid (1.4 mg, 1.67 μmol, 4%). HRMS (Bruker MicroTOF)(*m/z*): Calculated (M+H)^+^ for C_42_H_63_N_9_O_9_ = 838.4822; measured = 838.4829. Analytical HPLC (system 1): 98% purity; *t*_R_*=* 11.77 min.

#### (*S*)-2-((*S*)-2-((*S*)-2-(3-(4-(2-Benzylphenoxy)piperidin-1-yl)propanamido)-3-methylbutanamido)-propanamido)-*N*1-(3-(((*S*)-1,6-diamino-1-oxohexan-2-yl)amino)-3-oxo-propyl)succinamide (**29b**)

The title compound was synthesized following the method described in general procedure 1, using Fmoc-Lys(Boc)-OH (94 mg, 0.20 mmol) for the loading phase; Fmoc-ß-Ala-OH (63 mg, 0.20 mmol), Fmoc-Asn(Trt)-OH (120 mg, 0.20 mmol), Fmoc-Ala-OH (63 mg, 0.20 mmol), and Fmoc-Val-OH (68 mg, 0.20 mmol) for four separate coupling phases in the stated sequence; **7** (34 mg, 0.10 mmol) for the capping phase to afford a colorless solid (2.4 mg, 2.92 μmol, 7%). HRMS (Bruker MicroTOF)(*m/z*): Calculated (M+H)^+^ for C_42_H_63_N_9_O_8_ = 822.4872; measured = 822.4879. Analytical HPLC (system 1): 98% purity; *t*_R_*=* 11.82 min.

#### (*S*)-2-((*S*)-2-((2*S*,3*R*)-2-(3-(4-(2-Benzylphenoxy)piperidin-1-yl)propanamido)-3-hydroxybutanamido)-3-hydroxypropanamido)-*N*1-(3-(((S)-1,6-diamino-1-oxohexan-2-yl)amino)-3-oxopropyl)succinamide (**29c**)

The title compound was synthesized following the method described in general procedure 1, using Fmoc-Lys(Boc)-OH (94 mg, 0.20 mmol) for the loading phase; Fmoc-ß-Ala-OH (63 mg, 0.20 mmol), Fmoc-Asn(Trt)-OH (120 mg, 0.20 mmol), Fmoc-Ser(tBu)-OH (77 mg, 0.20 mmol), and Fmoc-Thr(tBu)-OH (80 mg, 0.20 mmol) for four separate coupling phases in the stated sequence; **7** (34 mg, 0.10 mmol) for the capping phase to afford a colorless solid (1.4 mg, 1.67 μmol, 4%). HRMS (Bruker MicroTOF)(*m/z*): Calculated (M+H)^+^ for C_41_H_64_N_9_O_10_ = 840.4614; measured = 840.4581. Analytical HPLC (system 1): 98% purity; *t_R_=* 11.45 min.

#### (*S*)-2-((*S*)-2-((2*S*,3*R*)-2-(3-(4-(2-Benzylphenoxy)piperidin-1-yl)propanamido)-3-hydroxybutanamido)propanamido)-*N*1-(3-(((*S*)-1,6-diamino-1-oxohexan-2-yl)amino)-3-oxopropyl)succinamide (**29d**)

The title compound was synthesized following the method described in general procedure 1, using Fmoc-Lys(Boc)-OH (94 mg, 0.20 mmol) for the loading phase; Fmoc-ß-Ala-OH (63 mg, 0.20 mmol), Fmoc-Asn(Trt)-OH (120 mg, 0.20 mmol), Fmoc-Ala-OH (63 mg, 0.20 mmol), and Fmoc-Thr(tBu)-OH (80 mg, 0.20 mmol) for four separate coupling phases in the stated sequence; **7** (34 mg, 0.10 mmol) for the capping phase to afford a colorless solid (1.9 mg, 2.31 μmol, 6%). HRMS (Bruker MicroTOF)(*m/z*): Calculated (M+H)^+^ for C_41_H_61_N_9_O_9_ = 824.4665; measured = 824.4663. Analytical HPLC (system 1): 98% purity; *t*_R_*=* 11.52 min.

#### (*S*)-6-Amino-2-((6*S*,9*S*,12*S*)-16-(4-(2-benzylphenoxy)piperidin-1-yl)-6-(4-hydroxybenzyl)-9-(hydroxymethyl)-12-isopropyl-5,8,11,14-tetraoxo-4,7,10,13-tetraaza-hexadecanamido)hexanamide (**29e**)

The title compound was synthesized following the method described in general procedure 1, using Fmoc-Lys(Boc)-OH (94 mg, 0.20 mmol) for the loading phase; Fmoc-ß-Ala-OH (63 mg, 0.20 mmol), Fmoc-Tyr(tBu)-OH (92 mg, 0.20 mmol), Fmoc-Ser(tBu)-OH (77 mg, 0.20 mmol), and Fmoc-Val-OH (68 mg, 0.20 mmol) for four separate coupling phases in the stated sequence; **7** (34 mg, 0.10 mmol) for the capping phase to afford a colorless solid (1.9 mg, 2.14 μmol, 5%). HRMS (Bruker MicroTOF)(*m/z*): Calculated (M+H)^+^ for C_47_H_66_N_8_O_9_ = 887.5026; measured = 887.5032. Analytical HPLC (system 1): 98% purity; *t*_R_*=* 12.23 min.

#### (*S*)-6-Amino-2-((6*S*,9*S*,12*S*)-16-(4-(2-benzylphenoxy)piperidin-1-yl)-6-(4-hydroxybenzyl)-12-isopropyl-9-methyl-5,8,11,14-tetraoxo-4,7,10,13-tetraazahexadecanamido)hexanamide (**29f**)

The title compound was synthesized following the method described in general procedure 1, using Fmoc-Lys(Boc)-OH (94 mg, 0.20 mmol) for the loading phase; Fmoc-ß-Ala-OH (63 mg, 0.20 mmol), Fmoc-Tyr(tBu)-OH (92 mg, 0.20 mmol), Fmoc-Ala-OH (63 mg, 0.20 mmol), and Fmoc-Val-OH (68 mg, 0.20 mmol) for four separate coupling phases in the stated sequence; **7** (34 mg, 0.10 mmol) for the capping phase to afford a colorless solid (2.4 mg, 2.76 μmol, 7%). HRMS (Bruker MicroTOF)(*m/z*): Calculated (M+H)^+^ for C_47_H_66_N_8_O_8_ = 871.5076; measured = 871.5036. Analytical HPLC (system 1): 98% purity; *t*_R_*=* 12.34 min.

#### (*S*)-6-Amino-2-((6*S*,9*S*,12*S*)-16-(4-(2-benzylphenoxy)piperidin-1-yl)-6-(4-hydroxy-benzyl)-12-((*R*)-1-hydroxyethyl)-9-(hydroxymethyl)-5,8,11,14-tetraoxo-4,7,10,13-tetraazahexadecanamido)hexanamide (**29g**)

The title compound was synthesized following the method described in general procedure 1, using Fmoc-Lys(Boc)-OH (94 mg, 0.20 mmol) for the loading phase; Fmoc-ß-Ala-OH (63 mg, 0.20 mmol), Fmoc-Tyr(tBu)-OH (92 mg, 0.20 mmol), Fmoc-Ser(tBu)-OH (77 mg, 0.20 mmol), and Fmoc-Thr(tBu)-OH (80 mg, 0.20 mmol) for four separate coupling phases in the stated sequence; **7** (34 mg, 0.10 mmol) for the capping phase to afford a colorless solid (1.9 mg, 2.14 μmol, 5%). HRMS (Bruker MicroTOF)(*m/z*): Calculated (M+H)^+^ for C_46_H_64_N_8_O_10_ = 889.4818; measured = 889.4811. Analytical HPLC (system 1): 98% purity; *t*_R_*=* 11.85 min.

#### (*S*)-6-Amino-2-((6*S*,9*S*,12*S*)-16-(4-(2-benzylphenoxy)piperidin-1-yl)-6-(4-hydroxybenzyl)-12-((*R*)-1-hydroxyethyl)-9-methyl-5,8,11,14-tetraoxo-4,7,10,13-tetraazahexadecanamido)hexanamide (**29h**)

The title compound was synthesized following the method described in general procedure 1, using Fmoc-Lys(Boc)-OH (94 mg, 0.20 mmol) for the loading phase; Fmoc-ß-Ala-OH (63 mg, 0.20 mmol), Fmoc-Tyr(tBu)-OH (80 mg, 0.20 mmol), Fmoc-Ala-OH (63 mg, 0.20 mmol), and Fmoc-Thr(tBu)-OH (68 mg, 0.20 mmol) for four separate coupling phases in the stated sequence; **7** (34 mg, 0.10 mmol) for the capping phase to afford a colorless solid (2.3 mg, 2.64 μmol, 7%). HRMS (Bruker MicroTOF)(*m/z*): Calculated (M+H)^+^ for C_46_H_64_N_8_O_9_ = 873.4869; measured = 873.4842. Analytical HPLC (system 1): 98% purity; *t*_R_*=* 12.00 min.

#### (*S*)-2-((*S*)-2-((2*S*,3*S*)-2-(3-(4-(2-Benzylphenoxy)piperidin-1-yl)propanamido)-3-methylpentanamido)-3-hydroxypropanamido)-*N*1-(3-(((*S*)-1,6-diamino-1-oxohexan-2-yl)amino)-3-oxopropyl)succinamide (**29i**)

The title compound was synthesized following the method described in general procedure 1, using Fmoc-Lys(Boc)-OH (94 mg, 0.20 mmol) for the loading phase; Fmoc-ß-Ala-OH (63 mg, 0.20 mmol), Fmoc-Asn(Trt)-OH (120 mg, 0.20 mmol), Fmoc-Ser(tBu)-OH (77 mg, 0.20 mmol), and Fmoc-Ile-OH (71 mg, 0.20 mmol) for four separate coupling phases in the stated sequence; **7** (34 mg, 0.10 mmol) for the capping phase to afford a colorless solid (2.3 mg, 2.70 μmol, 7%). HRMS (Bruker MicroTOF)(*m/z*): Calculated (M+H)^+^ for C_43_H_65_N_9_O_9_ = 852.4978; measured = 852.4969. Analytical HPLC (system 1): 98% purity; *t*_R_ = 12.30 min.

#### (*S*)-2-((*S*)-2-((*S*)-2-(3-(4-(2-Benzylphenoxy)piperidin-1-yl)propanamido)-4-methylpentanamido)-3-hydroxypropanamido)-*N*1-(3-(((*S*)-1,6-diamino-1-oxohexan-2-yl)amino)-3-oxopropyl)succinamide (**29j**)

The title compound was synthesized following the method described in general procedure 1, using Fmoc-Lys(Boc)-OH (94 mg, 0.20 mmol) for the loading phase; Fmoc-ß-Ala-OH (63 mg, 0.20 mmol), Fmoc-Asn(Trt)-OH (120 mg, 0.20 mmol), Fmoc-Ser(tBu)-OH (77 mg, 0.20 mmol), and Fmoc-Leu-OH (71 mg, 0.20 mmol) for four separate coupling phases in the stated sequence; **7** (34 mg, 0.10 mmol) for the capping phase to afford a colorless solid (1.3 mg, 1.53 μmol, 4%). HRMS (Bruker MicroTOF)(*m/z*): Calculated (M+H)^+^ for C_43_H_65_N_9_O_9_ = 852.4978; measured = 852.4964. Analytical HPLC (system 1): 94% purity; *t*_R_ = 12.62 min.

#### (*S*)-6-Amino-2-((6*S*,9*S*,12*S*)-12-benzyl-16-(4-(2-benzylphenoxy)piperidin-1-yl)-6,9-dimethyl-5,8,11,14-tetraoxo-4,7,10,13-tetraazahexadecanamido)hexanamide (**29k**)

The title compound was synthesized following the method described in general procedure 1, using Fmoc-Lys(Boc)-OH (94 mg, 0.20 mmol) for the loading phase; Fmoc-ß-Ala-OH (63 mg, 0.20 mmol), Fmoc-Ala-OH (63 mg, 0.20 mmol), Fmoc-Ala-OH (63 mg, 0.20 mmol), and Fmoc-Phe-OH (78 mg, 0.20 mmol) for four separate coupling phases in the stated sequence; **7** (34 mg, 0.10 mmol) for the capping phase to afford a colorless solid (2.5 mg, 3.02 μmol, 8%). HRMS (Bruker MicroTOF)(*m/z*): Calculated (M+H)^+^ for C_45_H_62_N_8_O_7_ = 827.4814; measured = 827.4807. Analytical HPLC (system 1): 98% purity; *t*_R_ = 13.02 min.

#### (*S*)-*N*1-(3-(((*S*)-1-Amino-6-(2-(4-((*E*)-2-(5,5-difluoro-7-(thiophen-2-yl)-5*H*-5λ^4^,6λ^4^-dipyrrolo[1,2-*c*:2’,1’-*f*][1,3,2]diazaborinin-3-yl)vinyl)phenoxy)acetamido)-1-oxohexan-2-yl)amino)-3-oxopropyl)-2-((*S*)-2-((*S*)-2-(3-(4-(2-benzylphenoxy)piperidin-1-yl)propanamido)-3-methylbutanamido)-3-hydroxypropanamido)succinamide (**31a**)

The title compound was synthesized following the method described in general procedure 3, using **29a** (1.4 mg, 1.67 μmol) to afford a blue solid (0.73 mg, 0.57 μmol, 34%). HRMS (Bruker MicroTOF)(*m/z*): Calculated (M+H)^+^ for C_65_H_78_B_1_F_2_N_11_O_11_S_1_ = 1270.5737; measured = 1270.5687. Analytical HPLC (system 1): 98% purity; *t*_R_*=* 17.97 min.

#### (*S*)-*N*1-(3-(((*S*)-1-Amino-6-(2-(4-((*E*)-2-(5,5-difluoro-7-(thiophen-2-yl)-5*H*-5λ^4^,6λ^4^-dipyrrolo[1,2-*c*:2’,1’-*f*][1,3,2]diazaborinin-3-yl)vinyl)phenoxy)acetamido)-1-oxohexan-2-yl)amino)-3-oxopropyl)-2-((*S*)-2-((*S*)-2-(3-(4-(2-benz-ylphenoxy)piperidin-1-yl)propanamido)-3-methylbutanamido)propanamido)succinamide (**31b**)

The title compound was synthesized following the method described in general procedure 3, using **29b** (2.4 mg, 2.92 μmol) to afford a blue solid (1.65 mg, 1.32 μmol, 45%). HRMS (Bruker MicroTOF)(*m/z*): Calculated (M+H)^+^ for C_65_H_78_B_1_F_2_N_11_O_10_S_1_ = 1254.5788; measured = 1254.5761. Analytical HPLC (system 1): 98% purity; *t*_R_*=* 18.15 min.

#### (*S*)-*N*1-(3-(((*S*)-1-Amino-6-(2-(4-((*E*)-2-(5,5-difluoro-7-(thiophen-2-yl)-5*H*-5λ^4^,6λ^4^-dipyrrolo[1,2-*c*:2’,1’-*f*][1,3,2]diazaborinin-3-yl)vinyl)phenoxy)acetamido)-1-oxohexan-2-yl)amino)-3-oxopropyl)-2-((*S*)-2-((*2S,3R*)-2-(3-(4-(2-benzylphenoxy)piperidin-1-yl)propanamido)-3-hydroxy-butanamido)-3-hydroxypropanamido)succinamide (**31c**)

The title compound was synthesized following the method described in general procedure 3, using **29c** (1.4 mg, 1.67 μmol) to afford a blue solid (0.83 mg, 0.65 μmol, 39%). HRMS (Bruker MicroTOF)(*m/z*): Calculated (M+H)^+^ for C_64_H_76_B_1_F_2_N_11_O_12_S_1_ = 1272.5530; measured = 1272.5508. Analytical HPLC (system 1): 98% purity; *t*_R_*=* 17.50 min.

#### (*S*)-*N*1-(3-(((*S*)-1-Amino-6-(2-(4-((*E*)-2-(5,5-difluoro-7-(thiophen-2-yl)-5*H*-5λ^4^,6λ^4^-dipyrrolo[1,2-*c*:2’,1’-*f*][1,3,2]diazaborinin-3-yl)vinyl)phenoxy)acetamido)-1-oxohexan-2-yl)amino)-3-oxopropyl)-2-((*S*)-2-((*2S,3R*)-2-(3-(4-(2-benzylphenoxy)piperidin-1-yl)propanamido)-3-hydroxybutanamido)propanamido)succinamide (**31d**)

The title compound was synthesized following the method described in general procedure 3, using **29d** (11.9 mg, 2.31 μmol) to afford a blue solid (0.95 mg, 0.76 μmol, 33%). HRMS (Bruker MicroTOF)(*m/z*): Calculated (M+H)^+^ for C_64_H_76_B_1_F_2_N_11_O_11_S_1_ = 1256.5580; measured = 1256.5538. Analytical HPLC (system 1): 98% purity; *t*_R_*=* 17.68 min.

#### (*S*)-2-((6*S*,9*S*,12*S*)-16-(4-(2-Benzylphenoxy)piperidin-1-yl)-6-(4-hydroxybenzyl)-9-(hydroxymethyl)-12-isopropyl-5,8,11,14-tetraoxo-4,7,10,13-tetraazahexadecanamido)-6-(2-(4-((*E*)-2-(5,5-difluoro-7-(thiophen-2-yl)-5*H*-5λ^4^,6λ^4^-dipyrrolo[1,2-*c*:2’,1’-*f*][1,3,2]diazaborinin-3-yl)vinyl)phenoxy)acetamido)hexanamide (**31e**)

The title compound was synthesized following the method described in general procedure 3, using **29e** (1.9 mg, 2.14 μmol) to afford a blue solid (1.11 mg, 0.84 μmol, 39%). HRMS (Bruker MicroTOF)(*m/z*): Calculated (M+H)^+^ for C_70_H_81_B_1_F_2_N_10_O_11_S_1_ = 1319.5941; measured = 1319.5883. Analytical HPLC (system 1): 97% purity; *t*_R_*=* 18.52 min.

#### (*S*)-2-((6*S*,9*S*,12*S*)-16-(4-(2-Benzylphenoxy)piperidin-1-yl)-6-(4-hydroxybenzyl)-12-isopropyl-9-methyl-5,8,11,14-tetraoxo-4,7,10,13-tetraazahexadecanamido)-6-(2-(4-((*E*)-2-(5,5-difluoro-7-(thiophen-2-yl)-5*H*-5λ^4^,6λ^4^-di-pyrrolo[1,2-*c*:2’,1’-*f*][1,3,2]diazaborinin-3-yl)vinyl)phenoxy)acetamido)hexanamide (**31f**)

The title compound was synthesized following the method described in general procedure 3, using **29f** (2.4 mg, 2.76 μmol) to afford a blue solid (0.96 mg, 0.74 μmol, 27%). HRMS (Bruker MicroTOF)(*m/z*): Calculated (M+H)^+^ for C_70_H_81_B_1_F_2_N_10_O_10_S_1_ = 1303.5992; measured = 1303.5943. Analytical HPLC (system 1): 98% purity; *t*_R_*=* 18.75 min.

#### (*S*)-2-((6*S*,9*S*,12*S*)-16-(4-(2-Benzylphenoxy)piperidin-1-yl)-6-(4-hydroxybenzyl)-12-((*R*)-1-hydroxyethyl)-9-(hydroxymethyl)-5,8,11,14-tetraoxo-4,7,10,13-tetraazahexadecanamido)-6-(2-(4-((*E*)-2-(5,5-difluoro-7-(thiophen-2-yl)-5*H*-5λ^4^,6λ^4^-dipyrrolo[1,2-*c*:2’,1’-*f*][1,3,2]diazaborinin-3-yl)vinyl)phenoxy)acetamido)hexanamide (**31g**)

The title compound was synthesized following the method described in general procedure 3, using **29g** (1.9 mg, 2.14 μmol) to afford a blue solid (0.77 mg, 0.58 μmol, 27%). HRMS (Bruker MicroTOF)(*m/z*): Calculated (M+H)^+^ for C_69_H_79_B_1_F_2_N_10_O_12_S_1_ = 1321.5734; measured = 1321.5741. Analytical HPLC (system 1): 98% purity; *t*_R_*=* 18.02 min.

#### (*S*)-2-((6*S*,9*S*,12*S*)-16-(4-(2-Benzylphenoxy)piperidin-1-yl)-6-(4-hydroxybenzyl)-12-((*R*)-1-hydroxyethyl)-9-methyl-5,8,11,14-tetraoxo-4,7,10,13-tetraazahexadecanamido)-6-(2-(4-((*E*)-2-(5,5-Difluoro-7-(thiophen-2-yl)-5*H*-5λ^4^,6λ^4^-dipyrrolo[1,2-*c*:2’,1’-*f*][1,3,2]diazaborinin-3-yl)vinyl)phenoxy)acetamido)hexanamide (**31h**)

The title compound was synthesized following the method described in general procedure 3, using **29h** (2.3 mg, 2.64 μmol) to afford a blue solid (1.04 mg, 0.80 μmol, 30%). HRMS (Bruker MicroTOF)(*m/z*): Calculated (M+H)^+^ for C_69_H_79_B_1_F_2_N_10_O_11_S_1_ = 1305.5784; measured = 1305.5757. Analytical HPLC (system 1): 98% purity; *t*_R_*=* 18.28 min.

#### (*S*)-*N*1-(3-(((S)-1-Amino-6-(2-(4-((*E*)-2-(5,5-difluoro-7-(thiophen-2-yl)-5*H*-5λ^4^,6λ^4^-dipyrrolo[1,2-*c*:2’,1’-*f*][1,3,2]diazaborinin-3-yl)vinyl)phenoxy)acetamido)-1-oxohexan-2-yl)amino)-3-oxopropyl)-2-((*S*)-2-((2*S*,3*S*)-2-(3-(4-(2-benzylphenoxy)piperidin-1-yl)propanamido)-3-methylpentanamido)-3-hydroxypropanamido)succinamide (**31i**)

The title compound was synthesized following the method described in general procedure 3, using **29i** (2.3 mg, 2.70 μmol) to afford a blue solid (1.02 mg, 0.78 μmol, 29%). HRMS (Bruker MicroTOF)(*m/z*): Calculated (M+H)^+^ for C_66_H_80_B_1_F_2_N_11_O_11_S_1_ = 1284.5893; measured = 1284.5828. Analytical HPLC (system 1): 95% purity; *t*_R_ = 17.92 min.

#### (*S*)-*N*1-(3-(((*S*)-1-Amino-6-(2-(4-((*E*)-2-(5,5-difluoro-7-(thiophen-2-yl)-5*H*-5λ^4^,6λ^4^-dipyrrolo[1,2-*c*:2’,1’-*f*][1,3,2]diazaborinin-3-yl)vinyl)phenoxy)acetamido)-1-oxohexan-2-yl)amino)-3-oxopropyl)-2-((*S*)-2-((*S*)-2-(3-(4-(2-benzylphenoxy)piperidin-1-yl)propanamido)-4-methylpentanamido)-3-hydroxypropanamido)succinamide (**31j**)

The title compound was synthesized following the method described in general procedure 3, using **29j** (1.3 mg, 1.53 μmol) to afford a blue solid (0.59 mg, 0.44 μmol, 29%). HRMS (Bruker MicroTOF)(*m/z*): Calculated (M+H)^+^ for C_66_H_80_B_1_F_2_N_11_O_11_S_1_ = 1284.5893; measured = 1284.5844. Analytical HPLC (system 1): 95% purity; *t*_R_ = 18.03 min.

#### (*S*)-2-((6*S*,9*S*,12*S*)-12-Benzyl-16-(4-(2-benzylphenoxy)piperidin-1-yl)-6,9-dimethyl-5,8,11,14-tetraoxo-4,7,10,13-tetraazahexadecanamido)-6-(2-(4-((*E*)-2-(5,5-difluoro-7-(thiophen-2-yl)-5*H*-5λ^4^,6λ^4^-dipyrr-olo[1,2-*c*:2’,1’-*f*][1,3,2]diazaborinin-3-yl)vinyl)phenoxy)acetamido)hexanamide (**31k**)

The title compound was synthesized following the method described in general procedure 3, using **29k** (2.5 mg, 3.02 μmol) to afford a blue solid (1.18 mg, 0.90 μmol, 30%). HRMS (Bruker MicroTOF)(*m/z*): Calculated (M+H)^+^ for C_68_H_77_B_1_F_2_N_10_O_9_S_1_ = 1259.5730; measured = 1259.5783. Analytical HPLC (system 1): 98% purity; *t*_R_ = 18.67 min.

#### Methyl (*E*)-2-(4-(2-(1*H*-Pyrrol-2-yl)vinyl)phenoxy)acetate (**32**)

(*E*)-2-(4-Methoxystyryl)-1*H*-pyrrole (**18**) (50 mg, 0.25 mmol) was dissolved in dry DMF (3 mL), and the reaction mixture was degassed by N_2_ sparging for 15 min. The reaction mixture was treated with sodium ethanethiolate (63 mg, 0.75 mmol) and heated to 145 °C for 18 h under N_2_. The reaction mixture was diluted with EtOAc (30 mL) and washed with 0.5 M NH_4_Cl (5 × 20 mL), followed by saturated brine, dried over MgSO_4_, and filtered, and the filtrate was concentrated over a rotary evaporator to afford a black solid. The solid was dissolved in acetonitrile (5 mL) and treated with K_2_CO_3_ (52 mg, 0.38 mmol), and the reaction mixture was heated to reflux for 30 min under N_2_. This was cooled to rt and treated with methyl bromoacetate (26 μL, 0.28 mmol), and the reaction mixture heated to reflux for 18 h. The reaction mixture was left to cool at rt, concentrated over a rotary evaporator, and diluted in EtOAc (30 mL). The organic layer was washed three times with water followed by saturated brine, dried over MgSO_4_, and filtered, and the filtrate was evaporated over a rotary evaporator afforded a green-black solid (44 mg, 0.17 mmol, 69%). ^1^H NMR (400 MHz, CDCl_3_) δ 8.35 (s, 1H, pyrrole-**H**), 7.35 (d, *J* = 8.8 Hz, 2H, 2 × Ph-C2**H**), 6.87 (d, *J* = 8.8 Hz, 2H, 2 × Ph-C3**H**), 6.85 (d, *J* = 16.7 Hz, 1H, -CHC**H**Ph), 6.81–6.77 (m, 1H, pyrrole-C5**H**), 6.61 (d, *J* = 16.5 Hz, 1H, −C**H**CHPh), 6.34–6.29 (m, 1H, pyrrole-C3**H**), 6.27–6.20 (m, 1H, pyrrole-C3**H**), 4.64 (s, 2H, −OC**H_2_**−), 3.81 (s, 3H, −OC**H_3_**). ^13^C NMR (101 MHz, CDCl_3_) δ 169.5, 157.0, 131.6, 131.1, 127.2, 122.9, 119.0, 117.9, 115.0, 114.3, 110.1, 108.8, 65.6, 52.4. LC-MS *m/z* calculated (M+H)^+^ for C_15_H_15_NO_3_ = 258.1, found = 257.8; *t*_R_*=* 2.81 min.

#### Methyl (*E*)-2-(4-(2-(5,5-Difluoro-7,9-dimethyl-5*H*-5λ^4^,6λ^4^-dipyrrolo[1,2-*c*:2’,1’-*f*][1,3,2]diaza-borinin-3-yl)vinyl)phenoxy)acetate (**34**)

Methyl (*E*)-2-(4-(2-(1*H*-pyrrol-2-yl)vinyl)phenoxy)acetate (**32**) (44 mg, 0.17 mmol) and 3,5-dimethyl-1*H*-pyrrole-2-carboxaldehyde (**33**) (21 mg, 0.17 mmol) were dissolved in dry DCM (10 mL), and the reaction mixture was degassed by N_2_ sparging for 15 min. The reaction mixture was treated dropwise with POCl_3_ (16 μL, 0.17 mmol) and stirred at rt for 2 h in the dark under N_2_. The mixture was subsequently diluted with DCM (40 mL) and treated with DIPEA (300 μL, 2.05 mmol) and boron trifluoride diethyl etherate (250 μL, 2.05 mmol), and the reaction mixture was stirred at rt for 18 h in the dark under N_2_. The reaction mixture was washed once with saturated brine, dried over MgSO_4_, and filtered, and the filtrate was concentrated over a rotary evaporator. The resulting gum was purified by silica gel column chromatography (5–50% EtOAc/hexane) to afford a dark purple solid (13 mg, 0.032 mmol, 19%). ^1^H NMR (400 MHz, DMSO-*d*_6_) δ 7.62 (s, 1H, Ar-C**H**), 7.60–7.45 (m, 3H, Ar-C**H**), 7.30 (d, *J* = 16.4 Hz, 1H, −C**H**CHPh), 7.18 (d, *J* = 4.3 Hz, 1H, Ar-C**H**), 7.09 (d, *J* = 4.3 Hz, 1H, Ar-C**H**), 7.00 (d, *J* = 8.8 Hz, 2H, 2 × Ph-C3**H**), 6.28 (s, 1H, Ar-C**H**), 4.84 (s, 2H, −OC**H_2_**−), 3.70 (s, 3H, −OC**H_3_**), 2.48 (s, 3H, pyrrole C3-C**H_3_**), 2.25 (s, 3H, pyrrole C5-C**H_3_**). ^13^C NMR (101 MHz, DMSO-*d*_6_) δ 169.0, 158.5, 157.8, 153.2, 142.9, 135.8, 134.7, 134.5, 129.5, 129.4, 128.6, 123.4, 119.9, 116.5, 115.8, 115.2, 64.6, 51.9, 14.6, 11.0. LC-MS *m/z* calculated (M+H)^+^ for C_22_H_21_BF_2_N_2_O_3_ = 411.2, found = 410.9; *t*_R_*=* 3.10 min.

#### (*E*)-2-(4-(2-(5,5-Difluoro-7,9-dimethyl-5*H*-5λ^4^,6λ^4^-dipyrrolo[1,2-*c*:2’,1’-*f*][1,3,2]di-aza-borinin-3-yl)vinyl)phenoxy)acetic Acid (**35**)

Methyl (*E*)-2-(4-(2-(5,5-difluoro-7,9-dimethyl-5*H*-5λ^4^,6λ^4^-dipyrrolo[1,2-*c*:2’,1’-*f*][1,3,2]diaza-borinin-3-yl)vinyl)phenoxy)acetate (**34**) (13 mg, 0.032 mmol) was dissolved in 2:1 THF/H_2_O (2.25 mL) and treated with 85% H_3_PO_4_ (50 μL, 0.433 mmol). The reaction mixture was heated under reflux at 65°C for 90 hr under N_2_. The reaction mixture was concentrated over a rotary evaporator, diluted with DCM (25 mL), washed once with sat. brine, dried over MgSO_4_, and filtered, and the filtrate was concentrated over a rotary evaporator and purified by silica gel column chromatography (10%-25% MeOH/DCM) to afford a dark purple solid (12 mg, 0.030 mmol, 92%). ^1^H NMR (400 MHz, DMSO-*d*_6_) δ 13.11 (s, 1H, COO**H**), 7.64 (s, 1H, Ar-C**H**), 7.61–7.47 (m, 3H, Ar-C**H**), 7.31 (d, *J* = 16.4 Hz, 1H, −C**H**CHPh), 7.19 (d, *J* = 4.4 Hz, 1H, Ar-C**H**), 7.12 (d, *J* = 4.3 Hz, 1H, Ar-C**H**), 7.00 (d, *J* = 8.8 Hz, 2H, 2 × Ph-C3**H**), 6.29 (s, 1H, Ar-C**H**), 4.73 (s, 2H, −OC**H_2_**−), 2.50 (s, 3H, pyrrole C3-C**H_3_**), 2.26 (s, 3H, pyrrole C5-C**H_3_**). ^13^C NMR (101 MHz, DMSO-*d*_6_) δ 169.9, 158.7, 157.6, 153.3, 142.7, 136.0, 134.7, 134.4, 129.5, 129.3, 128.5, 123.3, 119.8, 116.3, 115.8, 115.2, 64.6, 14.6, 11.0. LC-MS *m/z* calculated (M+H)^+^ for C_22_H_21_BF_2_N_2_O_3_ = 397.2, found = 396.7; *t*_R_*=* 2.96 min.

#### 3-(5,5-Difluoro-7-(thiophen-2-yl)-5*H*-5λ^4^,6λ^4^-dipyrrolo[1,2-*c*:2’,1’-*f*][1,3,2]diaza-borini-n-3-yl)propanoic Acid (**37**)

5-(Thiophen-2-yl)-1*H*-pyrrole-2-carbaldehyde (**15**) (50 mg, 0.282 mmol) and methyl 3-(1*H*-pyrrol-2-yl)propanoate (**36**) (44 mg, 0.282 mmol) were dissolved in dry DCM (10 mL), and the reaction mixture was degassed by N_2_ sparging for 15 min. The reaction mixture was treated dropwise with POCl_3_ (29 μL, 0.311 mmol) and stirred at rt for 2 h in the dark under N_2_. The reaction mixture was subsequently diluted with DCM (40 mL) and treated with DIPEA (850 μL, 3.39 mmol) and boron trifluoride diethyl etherate (1250 μL, 3.39mmol), and the reaction mixture was stirred at rt for 18 h in the dark under N_2_. The reaction mixture was washed once with saturated brine, dried over MgSO_4_, and filtered, and the filtrate was concentrated over a rotary evaporator and purified by silica gel column chromatography (5–30% EtOAc/hexane). The resulting gum was dissolved in 2:1 THF/H_2_O (2.7 mL), treated with 85% H_3_PO_4_ (100 μL, 0.866 mmol), and heated under reflux at 65 °C for 90 h under N_2_. The reaction mixture was concentrated over rotary evaporator, diluted in DCM (30 mL), washed once with saturated brine, dried over MgSO_4_, and filtered, and the filtrate was concentrated over a rotary evaporator and purified by silica gel column chromatography (5–7.5% MeOH/DCM) to afford a dark purple solid (15 mg, 0.043 mmol, 15% over two steps). ^1^H NMR (400 MHz, DMSO-*d*_6_) δ 12.36 (s, 1H, COO**H**), 8.03 (dd, *J* = 3.8, 1.1 Hz, 1H, thiophene-C**H**), 7.85 (dd, *J* = 5.1, 1.0 Hz, 1H, thiophene-C**H**), 7.70 (s, 1H, Ar-C**H**), 7.35 (d, *J* = 4.3 Hz, 1H, Ar-C**H**), 7.29 (d, *J* = 4.2 Hz, 1H, Ar-C**H**), 7.26 (dd, *J* = 5.0, 3.8 Hz, 1H, thiophene-C**H**), 6.98 (d, *J* = 4.3 Hz, 1H, Ar-C**H**), 6.55 (d, *J* = 4.2 Hz, 1H, Ar-C**H**), 3.17 (t, *J* = 7.7 Hz, 2H, -C**H_2_**CH_2_COOH), 2.69 (t, *J* = 7.7 Hz, 2H, -CH_2_C**H_2_**COOH). ^13^C NMR (101 MHz, DMSO-*d*_6_) δ 173.3, 161.0, 149.4, 136.3, 134.6, 133.2, 131.4, 131.0, 131.0, 128.9, 128.0, 120.0, 119.0, 32.1, 23.9 (1 carbon missing). HRMS (Bruker MicroTOF)(*m/z*): Calculated (M-H)^−^ for C_16_H_13_B_1_F_2_N_2_O_2_S_1_ = 345.0686; measured = 345.0680. LC-MS *m/z* calculated (M+H)^+^ for C_16_H_13_BF_2_N_2_O_2_S = 139.0, not found (likely due to poor ionization); *t*_R_*=* 2.80 min.

#### (*S*)-*N*^1^-(3-(((*S*)-1-Amino-6-(6-(2-(4-((*E*)-2-(5,5-difluoro-7-(thiophen-2-yl)-5*H*-4λ^4^,5λ^4^-dipyrrolo[1,2-*c*:2’,1’-*f*][1,3,2]diazaborinin-3-yl)vinyl)phenoxy)acetamido)hexanamido)-1-oxohexan-2-yl)amino)-3-oxopropyl)-2-((*S*)-2-((*S*)-2-(3-(4-(2-benzylphenoxy)piperidin-1-yl)propanamido)-3-methylbutanamido)-3-hydroxypropanamido)succinamide (**38a**)

The title compound was synthesized following the method described in general procedure 4, using **29a** (1.3 mg, 1.55 μmol) to afford a blue solid (1.28 mg, 0.93 μmol, 60%). HRMS (Bruker MicroTOF)(*m/z*): Calculated (M+H)^+^ for C_71_H_89_B_1_F_2_N_12_O_12_S_1_ = 1383.6578; measured = 1383.6513. Analytical HPLC (system 1): 98% purity; *t*_R_ = 16.31 min.

#### (*S*)-*N*^1^-(3-(((*S*)-1-Amino-6-(2-(4-((*E*)-2-(5,5-difluoro-7,9-dimethyl-5*H*-5λ^4^,6λ^4^-dipyrrolo[1,2-*c*:2’,1’-*f*][1,3,2]diazaborinin-3-yl)vinyl)phenoxy)acetamido)-1-oxohexan-2-yl)amino)-3-oxopropyl)-2-((*S*)-2-((*S*)-2-(3-(4-(2-benzylphenoxy)piperidin-1-yl)propanamido)-3-methylbutanamido)-3-hydroxypropanamido)succinamide (**38b**)

The title compound was synthesized following the method described in general procedure 3, using **29a** (0.6 mg, 0.72 μmol) to afford a purple solid (0.61 mg, 0.51 μmol, 71%). HRMS (Bruker MicroTOF)(*m/z*): Calculated (M+H)^+^ for C_63_H_80_B_1_F_2_N_11_O_11_ = 1216.6173; measured = 1216.6214. Analytical HPLC (system 1): 95% purity; *t*_R_ = 15.65 min.

#### (*S*)-*N*^1^-(3-(((*S*)-1-Amino-6-(3-(5,5-difluoro-7-(thiophen-2-yl)-5*H*-5λ^4^,6λ^4^-dipyrrolo[1,2-*c*:2’,1’-*f*][1,3,2]diazaborinin-3-yl)propanamido)-1-oxohexan-2-yl)amino)-3-oxopropyl)-2-((*S*)-2-((*S*)-2-(3-(4-(2-benzylphenoxy)piperidin-1-yl)propanamido)-3-methylbutanamido)-3-hydroxypropanamido)succinamide (**38c**)

The title compound was synthesized following the method described in general procedure 3, using **29a** (0.6 mg, 0.72 μmol) to afford a purple solid (0.37 mg, 0.32 μmol, 44%). HRMS (Bruker MicroTOF)(*m/z*): Calculated (M+H)^+^ for C_58_H_74_B_1_F_2_N_11_O_10_S_1_ = 1166.5475; measured = 1166.5450. Analytical HPLC (system 1): 96% purity; *t*_R_ = 14.80 min.

#### (*S*)-*N*^1^-(3-(((*S*)-1-Amino-6-(3-(5,5-difluoro-7,9-dimethyl-5*H*-4λ^4^,5λ^4^-dipyrrolo[1,2-*c*:2’,1’-*f*][1,3,2]diazaborinin-3-yl)propanamido)-1-oxohexan-2-yl)amino)-3-oxopropyl)-2-((*S*)-2-((*S*)-2-(3-(4-(2-benzylphenoxy)piperidin-1-yl)propanamido)-3-methylbutanamido)-3-hydroxypropanamido)succinamide (**38d**)

The title compound was synthesized following the method described in general procedure 4, using **29a** (0.6 mg, 0.72 μmol) to afford a lime-green solid (0.65 mg, 0.58 μmol, 81%). HRMS (Bruker MicroTOF)(*m/z*): Calculated (M+H)^+^ for C_56_H_76_B_1_F_2_N_11_O_10_ = 1112.5911; measured = 1112.5966. Analytical HPLC (system 1): 98% purity; *t*_R_ = 14.36 min.

#### (*S*)-*N*^1^-(3-(((*S*)-1-Amino-6-(6-(3-(5,5-difluoro-7,9-dimethyl-5*H*-4λ^4^,5λ^4^-dipyrrolo[1,2-*c*:2’,1’-*f*][1,3,2]diazaborinin-3-yl)propanamido)hexanamido)-1-oxohexan-2-yl)amino)-3-oxopropyl)-2-((*S*)-2-((*S*)-2-(3-(4-(2-benzylphenoxy)piperidin-1-yl)propanamido)-3-methylbutanamido)-3-hydroxypropanamido)succinamide (**38e**)

The title compound was synthesized following the method described in general procedure 4, using **29a** (1.3 mg, 1.55 μmol) to afford a lime-green solid (0.81 mg, 0.66 μmol, 43%). HRMS (Bruker MicroTOF)(*m/z*): Calculated (M+H)^+^ for C_62_H_87_B_1_F_2_N_12_O_11_ = 1225.6751; measured = 1225.6712. Analytical HPLC (system 1): 98% purity; *t*_R_ = 14.62 min.

#### 1-((5*S*,8*S*,11*S*,18*S*)-11-(2-Amino-2-oxoethyl)-1-(4-(2-benzylphenoxy)piperidin-1-yl)-18-carbamoyl-8-(hydroxymethyl)-5-isopropyl-3,6,9,12,16,24-hexaoxo-4,7,10,13,17,23-hexaazanonacosan-29-yl)-3,3-dimethyl-2-((1*E*,3*E*)-5-((*E*)-1,3,3-trimethyl-5-sulfonatoindolin-2-ylidene)penta-1,3-dien-1-yl)-3*H*-indol-1-ium-5-sulfonate (**38f**)

The title compound was synthesized following the method described in general procedure 4, using **29a** (1.3 mg, 1.55 μmol) to afford a blue solid (1.12 mg, 0.77 μmol, 50%). HRMS (Bruker MicroTOF)(*m/z*): Calculated M^-^ for C_74_H_98_N_11_O_16_S_2_ = 1460.6640; measured = 1460.6607. Analytical HPLC (system 1): 95% purity; *t*_R_ = 14.36 min.

#### *N*-((5*S*,8*S*,11*S*,18*S*)-5-Benzyl-1-(4-(2-benzylphenoxy)piperidin-1-yl)-18-carbamoyl-8,11-dimethyl-3,6,9,12,16-pentaoxo-4,7,10,13,17-pentaazadocosan-22-yl)-6-(2-(4-((*E*)-2-(5,5-difluoro-7-(thiophen-2-yl)-5*H*-4λ^4^,5λ^4^-dipyrrolo[1,2-*c*:2’,1’-*f*][1,3,2]diazaborinin-3-yl)vinyl)phenoxy)acetamido)hexanamide (**39a**)

The title compound was synthesized following the method described in general procedure 4, using **29k** (1.3 mg, 1.57 μmol) to afford a blue solid (1.81 mg, 1.32 μmol, 84%). HRMS (Bruker MicroTOF)(*m/z*): Calculated (M+H)^+^ for C_74_H_88_B_1_F_2_N_11_O_10_S_1_ = 1372.7570; measured = 1372.6578. Analytical HPLC (system 1): 99% purity; *t*_R_ = 16.89 min.

#### (*S*)-2-((6*S*,9*S*,12*S*)-12-Benzyl-16-(4-(2-benzylphenoxy)piperidin-1-yl)-6,9-dimethyl-5,8,11,14-tetraoxo-4,7,10,13-tetraazahexadecanamido)-6-(2-(4-((*E*)-2-(5,5-difluoro-7,9-dimethyl-5*H*-5λ^4^,6λ^4^-dipyrrolo[1,2-*c*:2’,1’-*f*][1,3,2]diazaborinin-3-yl)vinyl)phenoxy)acetamido)hexanamide (**39b**)

The title compound was synthesized following the method described in general procedure 3, using **29k** (0.6 mg, 0.72 μmol) to afford a purple solid (0.61 mg, 0.51 μmol, 71%). HRMS (Bruker MicroTOF)(*m/z*): Calculated (M+H)^+^ for C_66_H_79_B_1_F_2_N_10_O_9_ = 1205.6165; measured = 1205.6199. Analytical HPLC (system 1): 96% purity; *t*_R_ = 15.47 min.

#### (*S*)-2-((6*S*,9*S*,12*S*)-12-Benzyl-16-(4-(2-benzylphenoxy)piperidin-1-yl)-6,9-dimethyl-5,8,11,14-tetraoxo-4,7,10,13-tetraazahexadecanamido)-6-(3-(5,5-difluoro-7-(thiophen-2-yl)-5*H*-5λ^4^,6λ^4^-dipyrrolo[1,2-*c*:2’,1’-*f*][1,3,2]diazaborinin-3-yl)propanamido)hexanamide (**39c**)

The title compound was synthesized following the method described in general procedure 3, using **29k** (0.6 mg, 0.72 μmol) to afford a purple solid (0.32 mg, 0.32 μmol, 44%). HRMS (Bruker MicroTOF)(*m/z*): Calculated (M+H)^+^ for C_61_H_73_B_1_F_2_N_10_O_8_S_1_ = 1155.5467; measured = 1155.5496. Analytical HPLC (system 1): 96% purity; *t*_R_ = 15.48 min.

#### (*S*)-2-((6*S*,9*S*,12*S*)-12-Benzyl-16-(4-(2-benzylphenoxy)piperidin-1-yl)-6,9-dimethyl-5,8,11,14-tetraoxo-4,7,10,13-tetraazahexadecanamido)-6-(3-(5,5-difluoro-7,9-dimethyl-5*H*-4λ^4^,5λ^4^-dipyrrolo[1,2-*c*:2’,1’-*f*][1,3,2]diazaborinin-3-yl)propan-amido)hexanamide (**39d**)

The title compound was synthesized following the method described in general procedure 4, using **29k** (0.6 mg, 0.72 μmol) to afford a lime-green solid (0.66 mg, 0.60 μmol, 83%). HRMS (Bruker MicroTOF)(*m/z*): Calculated (M+H)^+^ for C_59_H_75_B_1_F_2_N_10_O_8_ = 1101.5903; measured = 1101.5961. Analytical HPLC (system 1): 99% purity; *t*_R_ = 15.12 min.

#### *N*-((5*S*,8*S*,11*S*,18*S*)-5-Benzyl-1-(4-(2-benzylphenoxy)piperidin-1-yl)-18-carbamoyl-8,11-dimethyl-3,6,9,12,16-pentaoxo-4,7,10,13,17-pentaazadocosan-22-yl)-6-(3-(5,5-difluoro-7,9-dimethyl-5*H*-4λ^4^,5λ^4^-dipyrrolo[1,2-*c*:2’,1’-*f*][1,3,2]diazaborinin-3-yl)propanamido)hexanamide (**39e**)

The title compound was synthesized following the method described in general procedure 4, using **29k** (1.3 mg, 1.57 μmol) to afford a lime-green solid (1.16 mg, 0.96 μmol, 61%). HRMS (Bruker MicroTOF)(*m/z*): Calculated (M+H)^+^ for C_65_H_86_B_1_F_2_N_11_O_9_ = 1214.6744; measured = 1214.6816. Analytical HPLC (system 1): 98% purity; *t*_R_ = 15.23 min.

#### 1-((5*S*,8*S*,11*S*,18*S*)-5-Benzyl-1-(4-(2-benzylphenoxy)piperidin-1-yl)-18-carbamoyl-8,11-dimethyl-3,6,9,12,16,24-hexaoxo-4,7,10,13,17,23-hexaazanonacosan-29-yl)-3,3-dimethyl-2-((1*E*,3*E*)-5-((*E*)-1,3,3-trimethyl-5-sulfonatoindolin-2-ylidene)penta-1,3-dien-1-yl)-3*H*-indol-1-ium-5-sulfonate (**39f**)

The title compound was synthesized following the method described in general procedure 4, using **29k** (1.3 mg, 1.57 μmol) to afford a blue solid (1.29 mg, 0.89 μmol, 57%). HRMS (Bruker MicroTOF)(*m/z*): Calculated M^-^ for C_77_H_87_N_10_O_14_S_2_ = 1449.6633; measured = 1449.6576. Analytical HPLC (system 1): 98% purity; *t*_R_ = 15.23 min.

### Pharmacology General Information

Fetal calf serum was obtained from PAA Laboratories (Wokingham, UK), furimazine from Promega (Southampton, UK), and all other chemicals and reagents were obtained from Sigma-Aldrich (Gillingham, UK) and Tocris Bioscience (Bristol, UK). Clobenpropit BODIPY 630/650^TM^

### Cell Culture

Nluc-tagged H_1_R expressing Human Embroynic Kidney (HEK)293T cells, H_1_R-YFP expressing Chinese Hamster Ovary (CHO) cells, and wild-type H_1_R expressing CHO cells were prepared as detailed in Stoddart et. al.^25^ HEK293T cells were maintained in Dulbecco’s modified Eagle’s medium (DMEM) supplemented with 10% fetal calf serum, whereas CHO cells were maintained in Dulbecco’s modified Eagle’s medium nutrient mix F12 (DMEM/F12) supplemented with 10% fetal calf serum and 2 mM of l-glutamine. All cell lines were maintained in T-75 flasks at 37 °C in a humidified atmosphere of air with 5% CO_2_. All cell culture procedures including cell plating for assays were performed in a class II laminar flow hood using sterile techniques.

### Spectral Characterization of Fluorescent Ligands

1 mM standard solution of the fluorescent ligand in DMSO was diluted to 0.1 mM with Hank’s balanced salt solution (HBSS; 145 mmol/L NaCl, 5 mmol/L KCl, 1.7 mmol/L CaCl_2_, 1 mmol/L MgSO_4_, 10 mmol/L HEPES, 2 mmol/L sodium pyruvate, 1.5 mmol/L NaHCO_3_, 10 mmol/L d-glucose, pH7.4), and 100 μL of the dilution was transferred to a black-walled, clear-bottom 96-well plate. The plate was then placed in multi-well fluorometric imaging plate reader FlexStation3 (Molecular Devices, Sunnyvale, CA). The excitation spectrum was determined by measuring the intensity of emission at 620 nm after excitation with light of increasing wavelengths ranging from 450–580 nm. The emission spectrum was determined by measuring the intensity of light emitted across a range of wavelengths 550–700 nm under 500 nm light emission.

### Transfection and Preparation of Cell Homogenates

HEK293T cells were transiently transfected with 2.5 μg of cDNA encoding Nluc-H_3_R or Nluc-H_4_R and 2.5 μg of empty pcDEF3 vector using the polyethylenimine method as described previously in Mocking et al.^23^ Two days after transfection, cells were collected in phosphate-buffered saline (PBS) and centrifuged at 1900*g* for 10 min and the cell pellet was stored at −20°C until the day of experiment. The pellet was resuspended in HBSS buffer, and cells were disrupted using a Branson sonifier 250 (Boom bv., Meppel, the Netherlands) prior to use in a NanoBRET binding assay.

### NanoBRET Binding Assay

For whole cell NanoBRET assays, HEK293T cells stably expressing Nluc-tagged H_1_R were seeded in white 96-well microplates and grown for 24 h prior to experimentation in normal growth medium. The media was replaced with HBSS right before experimentation. For whole cell saturation binding experiments, the required concentrations of fluorescent ligand and the competing unlabeled ligand mepyramine (final concentration of 10 μM) were added simultaneously to the plate and incubated for 2 h at 37 °C in the absence of CO_2_. For whole cell competition binding experiments, the required concentrations of unlabeled ligand and the competing fluorescent ligand **1** (final concentration of 25 nM) were added simultaneously and incubated for 2 h at 37 °C in the absence of CO_2_.

For cell membrane NanoBRET saturation binding experiments, cell homogenates expressing the Nluc-H_3_R or Nluc-H_4_R were incubated with the required concentrations of fluorescent ligand in the absence and presence of 10 μM clobenpropit in HBSS for 2 h at 25 °C. For all NanoBRET binding experiments, 10 μM NanoGlo substrate (Promega) was added to each well after the incubation period and fluorescence and luminescence emissions were measured after 5 min.

For whole cell NanoBRET association kinetic experiments, 10 μM furimazine was added to each well and incubated at room temperature in the dark for 15 min to allow for stabilization of the luminescence signal. The required concentration of fluorescent ligand in the presence or absence of 10 μM of doxepin was added simultaneously, and the plates were read immediately with each well being read once per min for 90 min.

Fluorescence and luminescence were read simultaneously using a PHERAstar FS plate reader (BMG Labtech, Aylesbury, UK) at 37 °C. Luminescence was measured at 392.5–467.5 nm for green-shifted fluorescent ligands **38d,e** and **39d,e** and 420–500 nm for orange and red-shifted fluorescent ligands **22a**–**c**, **28a**–**d**, **31a**–**k**, **38a**–**c, 38f, 39a**–**c**, and **39f**. Fluorescence was measured at 512.5–547.5 nm for green-shifted fluorescent ligands **38d**,**e** and **39d**,**e**; >550 nm (longpass) for orange-shifted fluorescent ligands **38a**,**b** and **39a**,**b**; >610 nm (longpass) for red-shifted fluorescent ligands **22a**–**c**, **28a**–**d**, **31a**–**k**, **38a, 38f, 39a**, and **39f**. The raw BRET ratio was calculated by dividing the fluorescence value by the luminescence value.

### Intracellular Calcium Mobilization Assay

CHO cells expressing H_1_R were grown to confluence in black-walled, clear-bottom 96-well plates. On the day of the experiment, media were replaced with 100 μL of HBSS containing 2.5 nM probenecid, 2.3 μM Fluo-4AM (Invitrogen), 0.023% Pluronic F-127, 0.5 mM Brilliant Black BN, and 1 μM required fluorescent ligand and the plates incubated for 1 h at 37 °C in the dark. The plates were then placed in the multi-well fluorometric imaging plate reader FlexStation3 (Molecular Devices, Sunnyvale, CA) and HBSS only or HBSS containing the required concentration of histamine was added after 15 s. Fluo-4 fluorescence was measured at 520 nm (excitation at 485 nm) every 1.52 s for 200 s. For each well, the peak maximum–minimum–maximum change in fluorescence value was taken and plotted against concentration of histamine to give a dose–response curve.

### Confocal Imaging

H_1_R-YFP expressing CHO cells were grown to approximately 80% confluency on an 8-well Labtek chambered coverglass (Nunc Nalgene). On the day of experiment, the cells were washed two times with 200 μL of HBSS and subsequently incubated in the presence or absence of 10 μM mepyramine for 30 min at 37 °C in humidified air with 0% CO_2_. The required concentration of **31a** was added to the wells and incubated for another 30 min at 37 °C. Images were acquired using a Zeiss LSM710 confocal microscope (Carl Zeiss GmbH, Hena, Germany) fitted with a 63× plan-Apochromat NA1.3 Ph3 oil-immersion lens. For YFP, a 488 nm argon laser was used for excitation and emission was detected using a BP505-30 filter. For fluorescent ligand **31a**, a 633 nm helium-neon laser was used for excitation and emission was detected using a 650 nm long pass filter. A pinhole of 1 Airy Unit was used, and fixed laser power, gain, and offset for the BODIPY 630/650 containing compounds were kept constant across all experiments.

### Data Analysis

All data were analyzed and presented using Prism 7 (GraphPad Software, San Diego, CA).

### Analysis of NanoBRET Binding Experiments

Total and non-specific saturation binding curves from NanoBRET saturation binding assay were fitted simultaneously using eq 1:
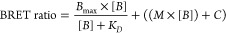
1where *B*_max_ is the maximal specific binding, [*B*] is the concentration of fluorescent ligand in nM, *K*_D_ is the equilibrium dissociation constant in nM, *M* is the slope of the non-specific component, and *C* is the intercept with the *Y* axis.

NanoBRET competition binding curves were fitted using the Cheng-Prusoff equation (2):
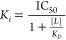
2where [*L*] is the concentration of the fluorescent ligand **1** in nM and *K*_D_ is the equilibrium dissociation constant of **1** in nM (*K*_D_ = 8 nM). The IC_50_ is calculated as in eq 3:
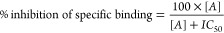
3where [*A*] is the concentration of the unlabeled competing drug and IC_50_ is the molar concentration of this competing ligand required to inhibit 50% of the specific binding of the concentration [*L*] of the fluorescent ligand.

For the NanoBRET association kinetic experiments, the BRET ratio was determined for each concentration of fluorescent ligand at each time point in the presence or absence of 10 μM of doxepin. Specific binding was calculated by subtracting non-specific binding from total binding. The *k*_on_, *k*_off_, and *K*_D_ values were obtained from the data using eq 4:
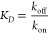
4where *K*_D_ is the equilibrium dissociation constant and *k*_off_ is the dissociation rate constant of the ligand in min^–1^. *k*_on_ is the association rate constant in M^–1^ min^–1^ and is calculated as follows in eq 5:
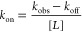
5where [*L*] is the ligand concentration in M and *k*_obs_ is calculated from global fitting of the data to the following equation, which expresses monoexponential association function (6):
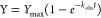
6where *Y*_max_ equals levels of binding at infinite time (*t*) and *k*_obs_ is the rate constant for the observed rate of association.

### Analysis of Ca^2+^ Mobilization Experiment

Estimated affinity values (p*K*_B_) were calculated from the shift in agonist concentration–response curves in the presence of the fluorescent antagonists using the Gaddum equation (7):
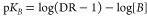
7where DR (dose ratio) is the ratio of the agonist concentration required to stimulate an identical response in the presence and absence of antagonist, [*B*]. DR was determined from the EC_25_ value as there was a decrease in observed maximal efficacy of histamine in the presence of all 10 fluorescent antagonists. The EC_25_ value was determined from the nonlinear regression fit of the normalized maximum–minimum fluorescence values using eq 8:
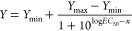
8where *Y*_min_ and *Y*_max_ are the lowest and highest normalized maximum–minimum fluorescence value and EC_50_ is the concentration of the histamine at 50% response.

### Molecular Docking Simulation

The H_1_R crystal structure (PDB code: 3RZE) was obtained from the protein data bank and prepared by using Protein Preparation Wizard within Maestro of the Schrodinger modeling suite. Missing atoms (in K442, R481) and missing residues (F168–V174 in extracellular loop 2) were modeled, phosphate ions, water molecules, and the *Z*-isomer of doxepin (D7V) were removed retaining only the *E*-isomer (5EH), hydrogen bondings were optimized using PROPKA^62,63^ at pH 7, and the protein structure was minimized using the OPLS3 force field.^64^ Ligands for docking were prepared and minimized using LigPrep^65^ within the modeling suite and docked into the receptor using Glide under XP (extra precision)^66^ mode without imposing any restraints using the OPLS3 force field and flexible ligand sampling. The sampling space encompassed the orthosteric binding domain and the entire space above it. The binding poses that formed the crucial salt bridge interaction with Asp107 were selected and examined using Maestro within the Schrodinger modeling suite. This was modeled using PyMOL 2.1.1^67^ to include amino acid labels and distance measurements between the fluorescent ligand and nearby receptor residues.

### Membrane Bilayer Modeling

The T4-lysozyme of the H_1_R crystal structure (PDB: 3RZE) was truncated on PyMOL 2.1.1 and saved as a PDB file. The membrane bilayer was modeled using the ProBLM web server^50^ by uploading the PDB file as input and selecting POPC-75x75 (phospholiphase C) as the membrane file on the server. The membrane bilayer model was subsequently aligned with the docking pose of **31a** using PyMOL 2.1.1.^67^

## Supporting Information Available

Molecular formula strings of tested compounds (CSV)
PDB file (PDB)
Preliminary docking studies showing the predicted binding pose of **8a** at the human H_1_R (PDB code: 3RZE), absorption/emission spectral profiles of **38b,c** and **39b,c**, saturation binding curves of tested fluorescent ligands, and analytical reverse-phase HPLC chromatogram of **31a** and **31k** (PDF)

## Supplementary Material

jm2c00125_si_001.csv

jm2c00125_si_002.pdb

## ABBREVIATIONS

BODIPY: borondipyrromethene
BRET: bioluminescence resonance energy transfer
CHO: Chinese hamster ovary
CuAAC: copper-catalyzed alkyne-azide cycloaddition
Cy5: cyanine 5
DIPEA: *N*,*N*-diisoproylethylamine
GPCR: G protein-coupled receptor
H_1_R: histamine H_1_ receptor
H_3_R: histamine H_3_ receptor
H_4_R: histamine H_4_ receptor
HATU: 1-[bis(dimethylamino)methylene]-1*H*-1,2,3-triazolo[4,5-*b*]pyridinium 3-oxide hexafluorophosphate
HBTU: 2-(1*H*-benzotriazol-1-yl)-1,1,3,3-tetramethyluronium hexafluorophosphate
HOBt: hydroxybenzotriazole
*k*_on_: association rate
*k*_off_: dissociation rate
MRT: mean residence time
NHS: *N*-hydroxysuccinimide
Nluc: nanoluciferase
NanoBRET: nanoluciferase-bioluminescence resonance energy transfer
SPPS: solid-phase peptide synthesis
TFA: trifluoroacetic acid
YFP: yellow fluorescence protein
