# Supplementary material for: Optimization of Peptide Linker-Based Fluorescent Ligands for the Histamine H1 Receptor
Source: J Med Chem. 2022 Jun 3;65(12):8258–88. doi: 10.1021/acs.jmedchem.2c00125 (PMC9234962; doi:10.1021/acs.jmedchem.2c00125)

Supporting Information

Optimization of Peptide Linker-based Fluorescent Ligands for the Histamine H_1 Receptor

Zhi Yuan Kok,[†, §] Leigh A. Stoddart,[‡,§] Sarah J. Mistry,[†, §] Tamara A. M. Mocking,[∥] Henry F. Vischer,[∥] Rob Leurs,[∥] Stephen J. Hill,[,‡,§] Shailesh N. Mistry,[*, †] Barrie Kellam[*,†,§]*

[†]Division of Biomolecular Science and Medicinal Chemistry, School of Pharmacy, University of Nottingham Biodiscovery Institute, University Park, Nottingham NG7 2RD, U.K.

[‡]Division of Physiology, Pharmacology & Neuroscience, Medical School, School of Life Sciences, University of Nottingham, Nottingham NG7 2UH, U.K.

[§]Centre of Membrane Proteins and Receptors, University of Birmingham and University of Nottingham, the Midlands NG7 2UH, U.K.

[∥]Amsterdam Institute for Molecules, Medicines and Systems, Division of Medicinal Chemistry, Faculty of Science, Vrije Universiteit Amsterdam, De Boelelean 1083, 1083 HV Amsterdam, The Netherlands.

Corresponding Authors

*Barrie Kellam: e-mail, barrie.kellam@nottingham.ac.uk; tel., +44 (0)115 951 3026.
*Shailesh N. Mistry: email, shailesh.mistry@nottingham.ac.uk; tel., +44 (0)115 846 7983.
*Stephen J. Hill: email, stephen.hill@nottingham.ac.uk; tel., +44 (0)115 823 0082.

Table of Contents

Figure S1. The predicted binding pose of **8a** (green) at the human H_1R. S3

Figure S2. Absorption and emission spectra of **38b** S4

Figure S3. Absorption and emission spectra of **38c** S5

Figure S4. Absorption and emission spectra of **39b** S6

Figure S5. Absorption and emission spectra of **39c** S7

Figure S6. Overview of H_1R fluorescent ligands synthesized S8

Figure S7. Saturation binding curves of **22a-c** aNluc-H_1R from NanoBRET assay S9

Figure S8. Saturation binding curves of **28a-d** aNluc-H_1R from NanoBRET assay S10

Figure S9. Saturation binding curves of **31a-f** at Nluc-H_1R from NanoBRET assay S11

Figure S10. Saturation binding curves of **31g-k** at Nluc-H_1R from NanoBRET assay S12

Figure S11. Saturation binding curves of **38a-f** at Nluc-H_1R from NanoBRET assay S13

Figure S12. Saturation binding curves of **39a-f** at Nluc-H_1R from NanoBRET assay S14

Figure S13. Displacement curve of **VUF14454** at Nluc-H_3R and Nluc-H_4R from NanoBRET assay S15

Figure S14. Analytical RP-HPLC chromatogram for **31a** S16

Figure S15. Analytical RP-HPLC chromatogram for **31k** S17

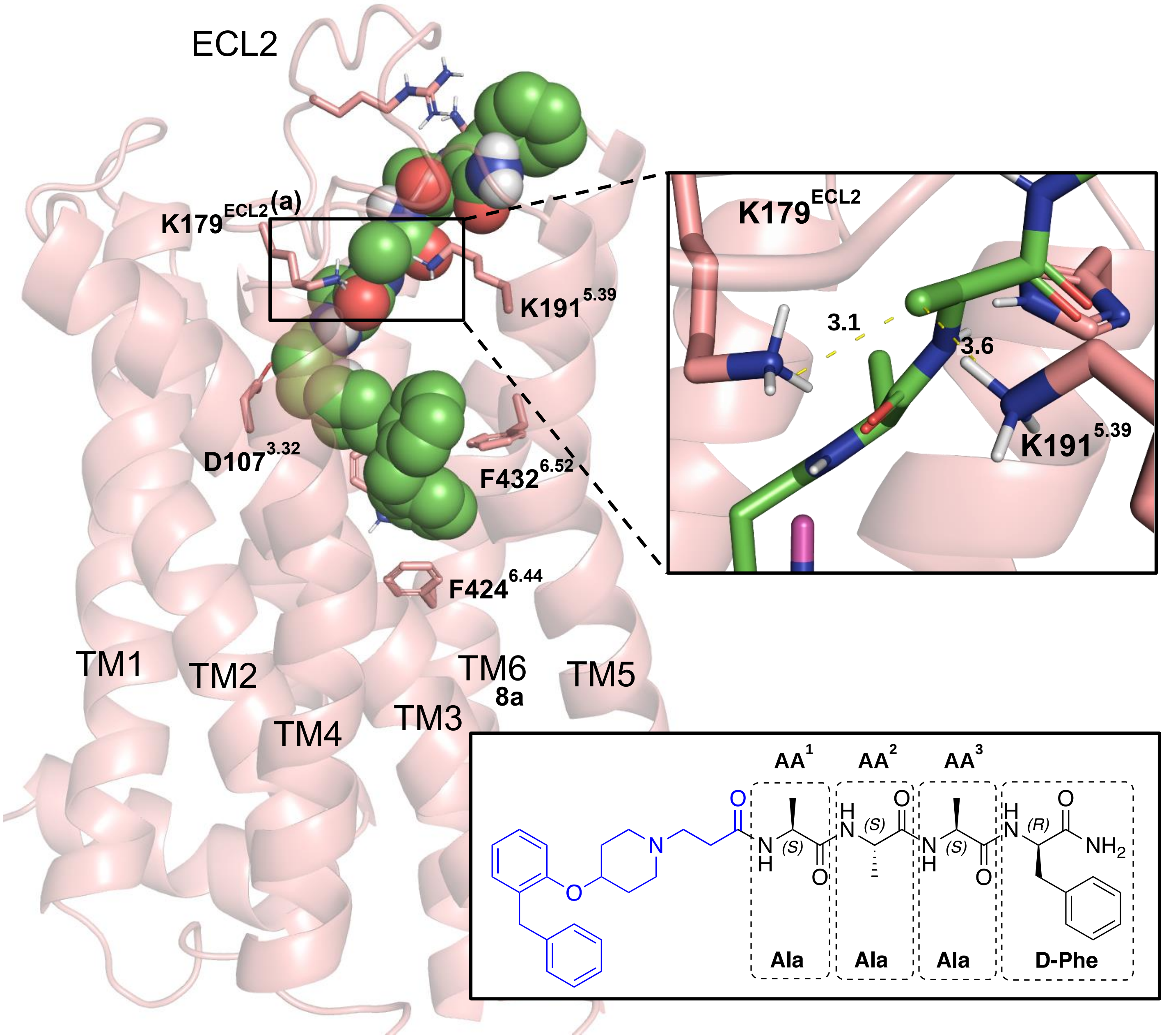

Figure S1. The predicted binding pose of **8a** (green) at the human H_1R (PDB code: 3RZE) determined using Glide docking. The transmembrane helices (TM) and amino acid residues which are predicted to interact with the congener are labelled. The region around AA^2 is magnified with distances of 3.1Å and 3.6Å between the methyl side chain and $K179^{ECL2}$ and $K191^{5.39}$ respectively.

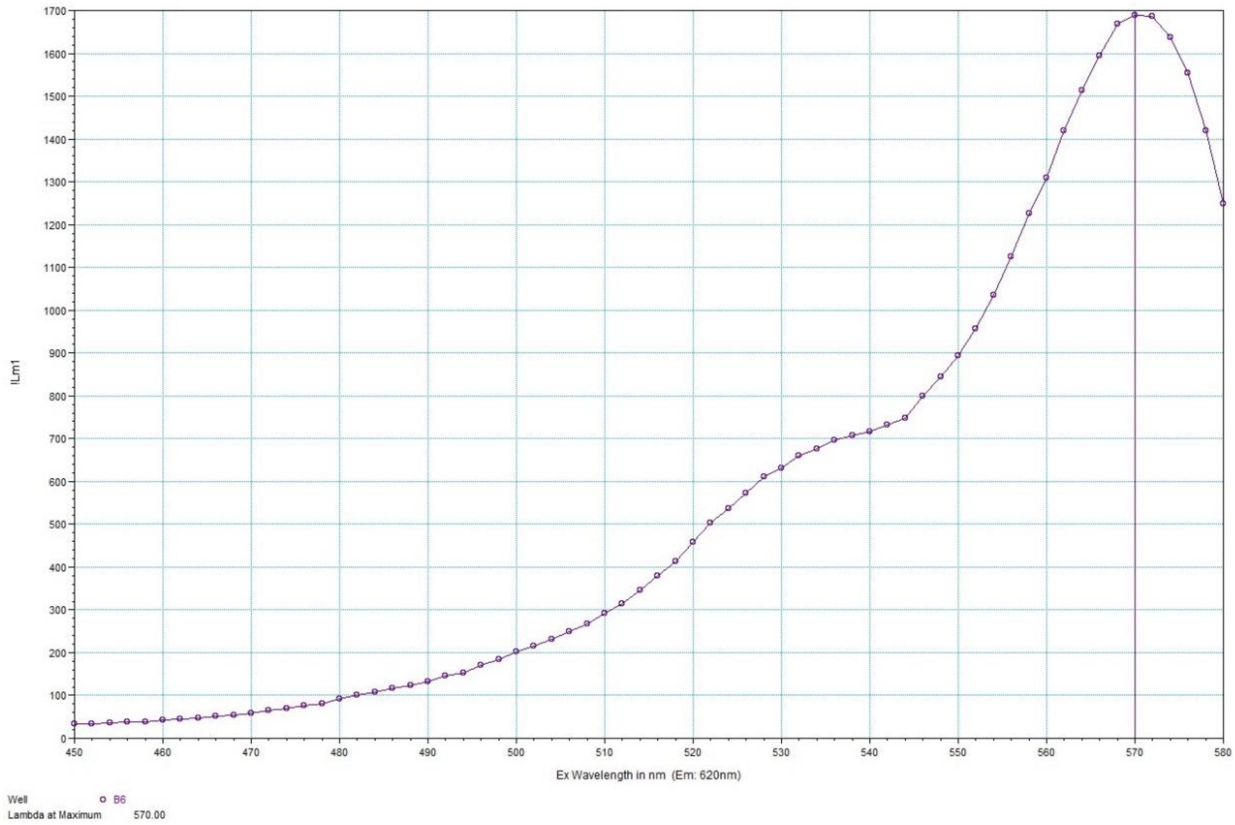

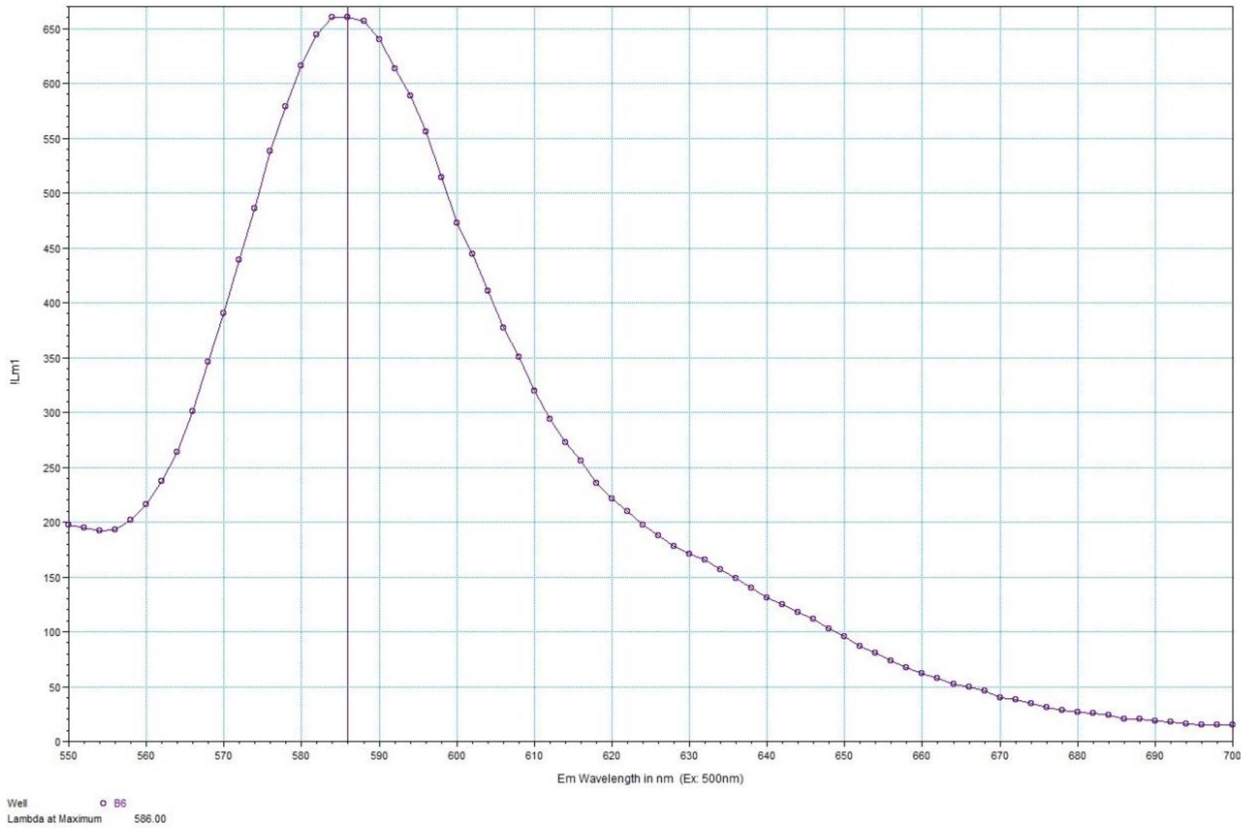

Figure S2. Absorption (top) and emission (bottom) spectra of **38b**.

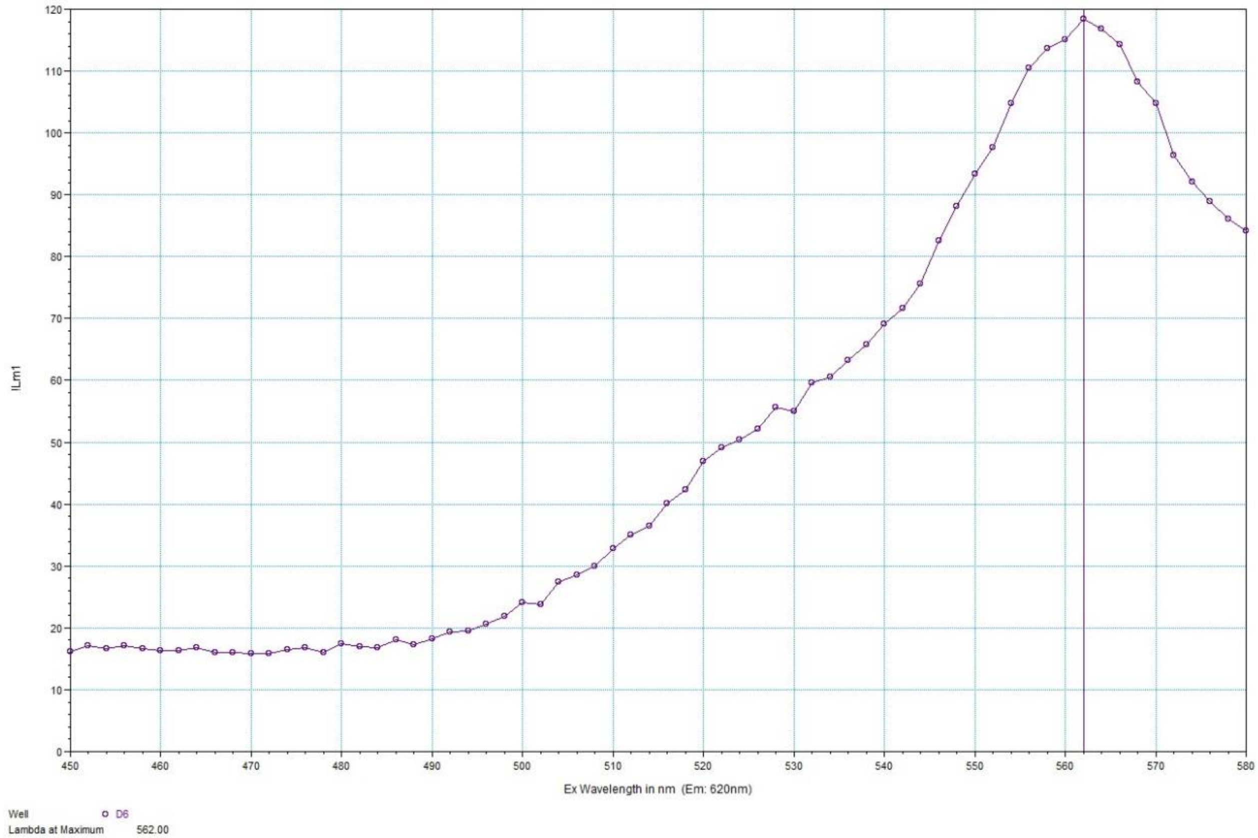

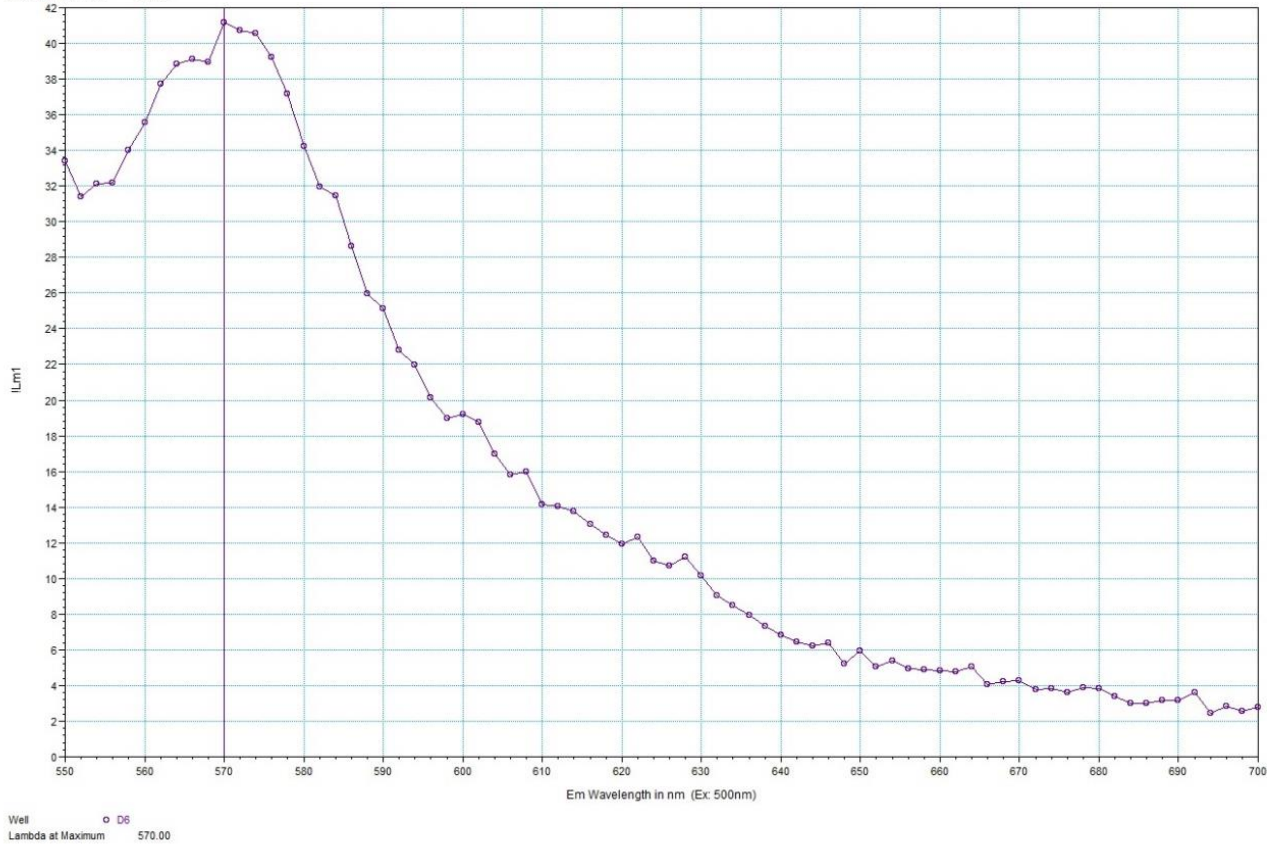

Figure S3. Absorption (top) and emission (bottom) spectra of **38c**.

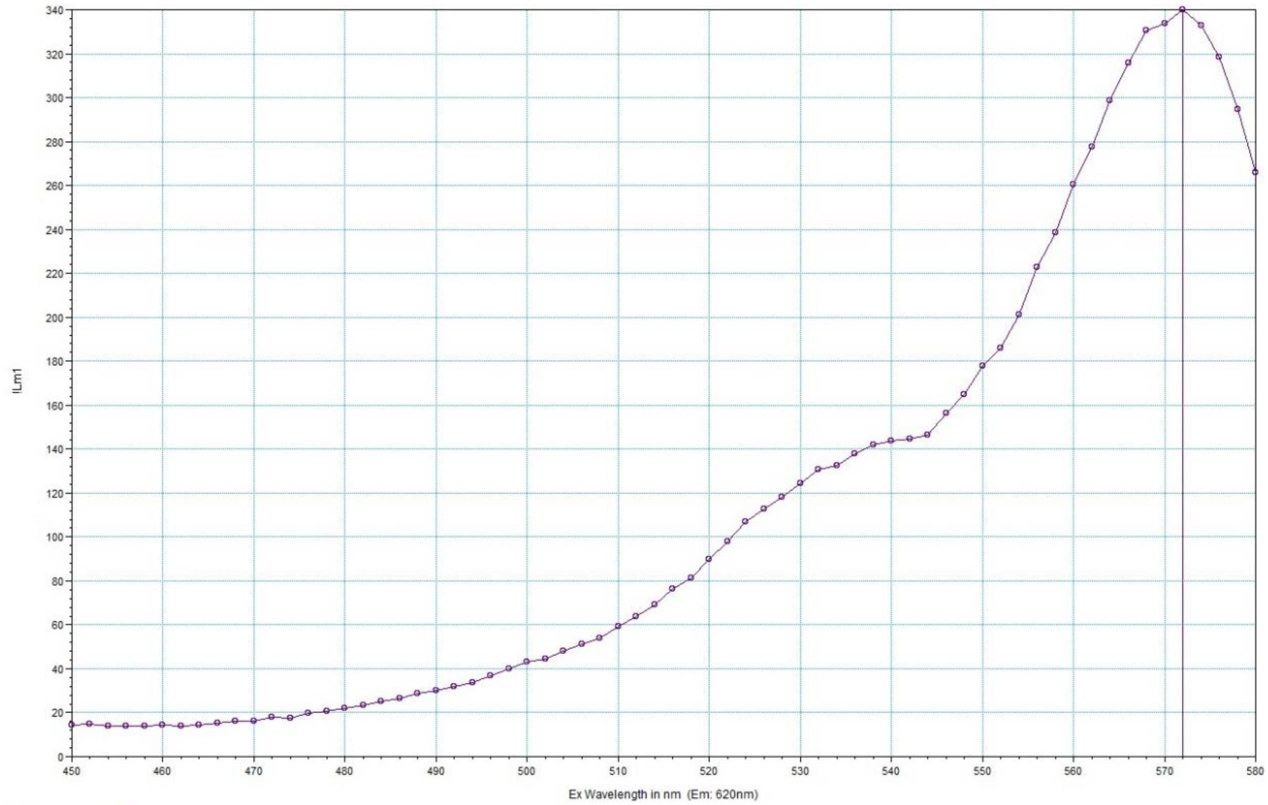

Well o C6
Lambda at Maximum 572.00

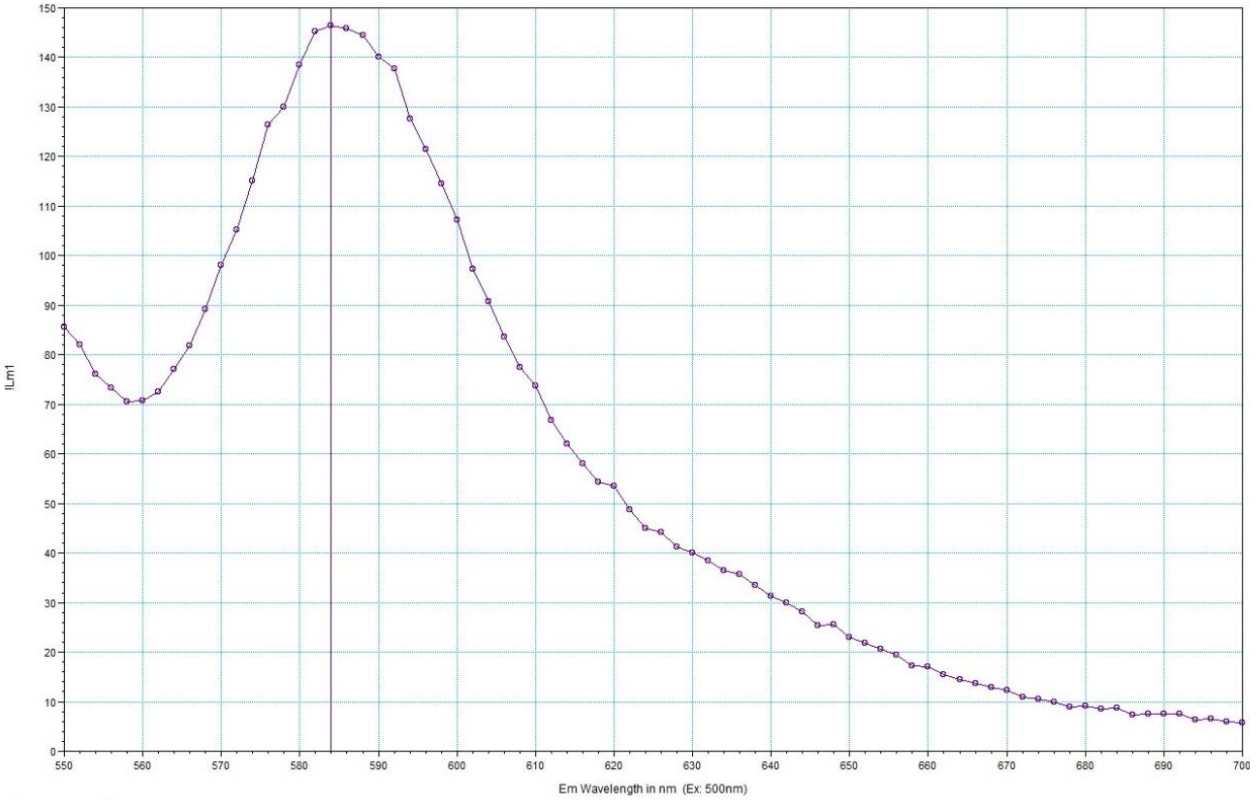

Well o C6
Lambda at Maximum 584.00

Figure S4. Absorption (top) and emission (bottom) spectra of **39b**.

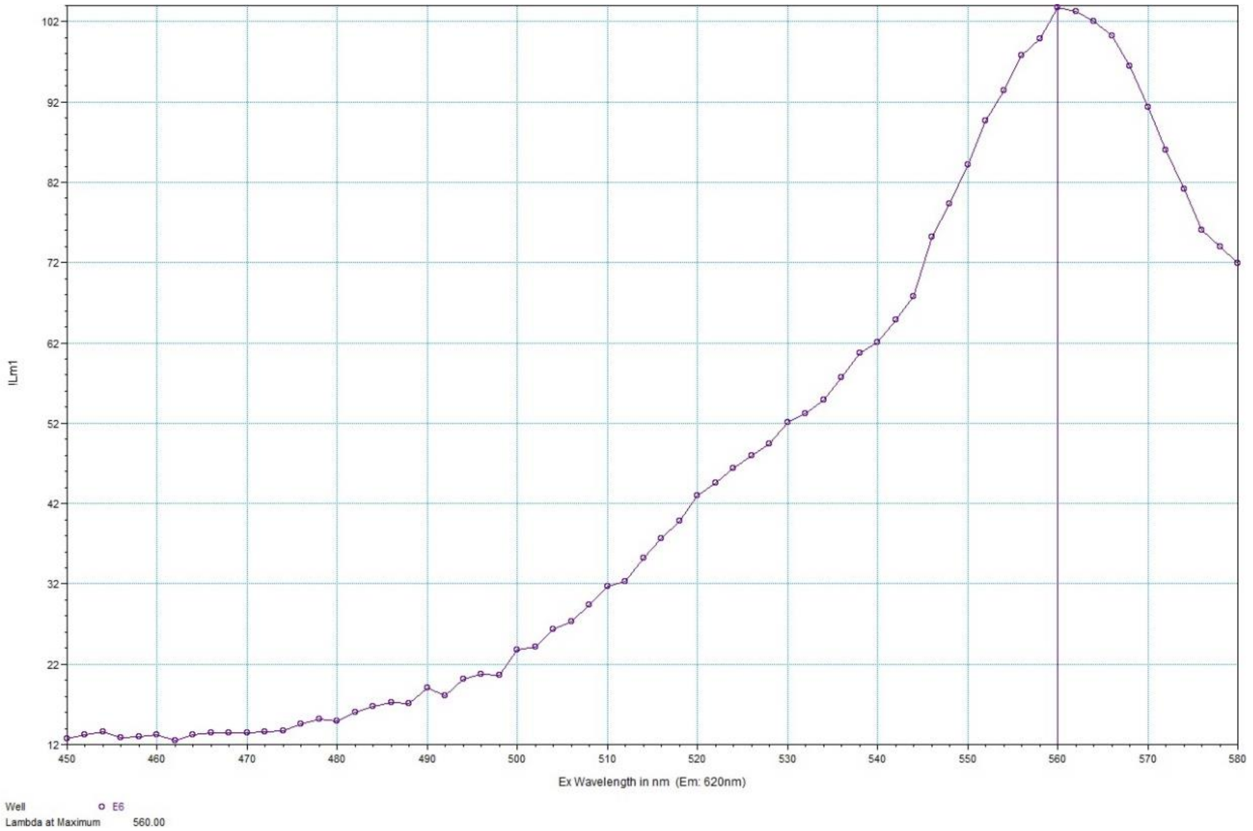

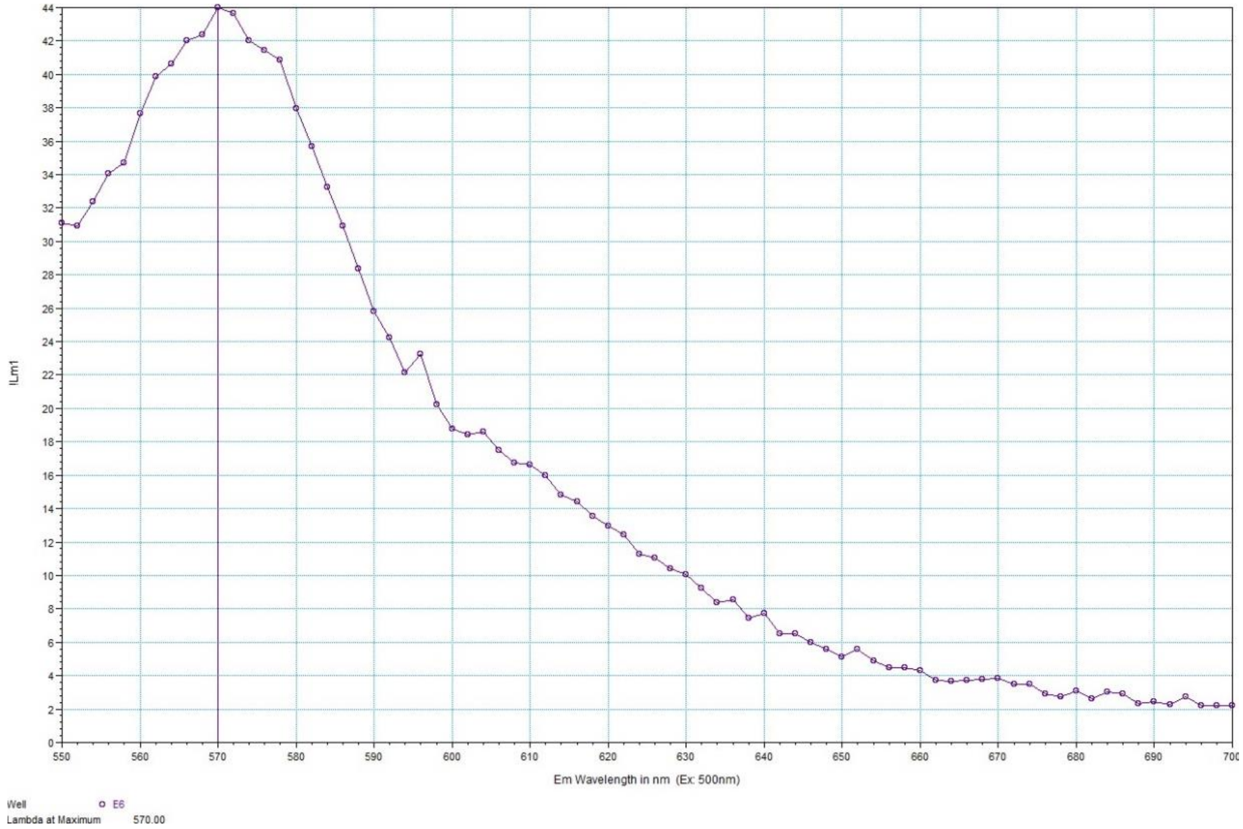

Figure S5. Absorption (top) and emission (bottom) spectra of **39c**.

Copper-catalyzed Azide-Alkyne Cycloaddition (CuAAC)-coupled Fluorescent Ligands

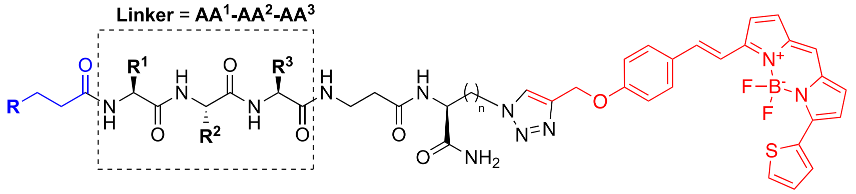

22a-c, 28a-d

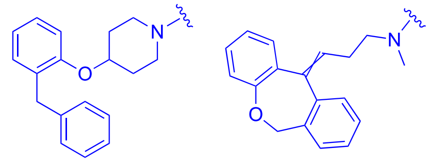

VUF13816 desmethyldoxepin

22a-c: n = 1, **28a-d:** n = 3,

22a: R = VUF13816, linker = Val-Ser-Asn
22b: R = VUF13816, linker = Ala-Ala-Ala
22c: R = VUF13816, linker = Ala-Glu-Ser

28a: R = VUF13816, linker = Val-Ser-Asn
28b: R = VUF13816, linker = Ala-Ala-Ala
28c: R = desmethyldoxepin, linker = Val-Ser-Asn
28d: R = desmethyldoxepin, linker = Ala-Ala-Ala

Amide-coupled Fluorescent Ligands

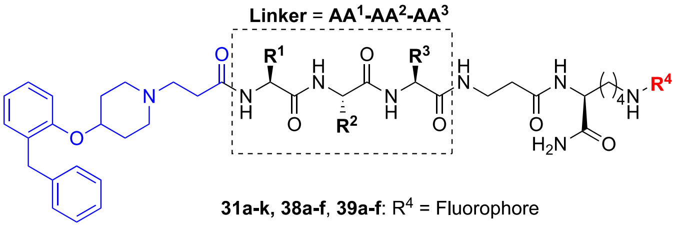

31a-k, 38a-f, 39a-f: R^4 = Fluorophore

31a-k: R = VUF13816, n = 4, R^4 = BODIPY 630/650™-COOH, Linker =

**31a**: Val-Ser-Asn	**31g**: Thr-Ser-Tyr
**31b**: Val-Ala-Asn	**31h**: Thr-Ala-Tyr
**31c**: Thr-Ser-Asn	**31i**: Ile-Ser-Asn
**31d**: Thr-Ala-Asn	**31j**: Leu-Ser-Asn
**31e**: Val-Ser-Tyr	**31k**: Phe-Ala-Ala
**31f**: Val-Ala-Tyr	

38a-f: R = VUF13816, n = 4, Linker = Val-Ser-Asn, R^4 =

**38a**: BODIPY 630/650™-X	**38d**: BODIPY FL
**38b**: BODIPY A	**38e**: BODIPY FL-X
**38c**: BODIPY B	**38f**: Sulfo-Cy5

39a-f: R = VUF13816, n = 4, Linker = Phe-Ala-Ala, R^4 =

**39a**: BODIPY 630/650™-X	**39d**: BODIPY FL
**39b**: BODIPY A	**39e**: BODIPY FL-X
**39c**: BODIPY B	**39f**: Sulfo-Cy5

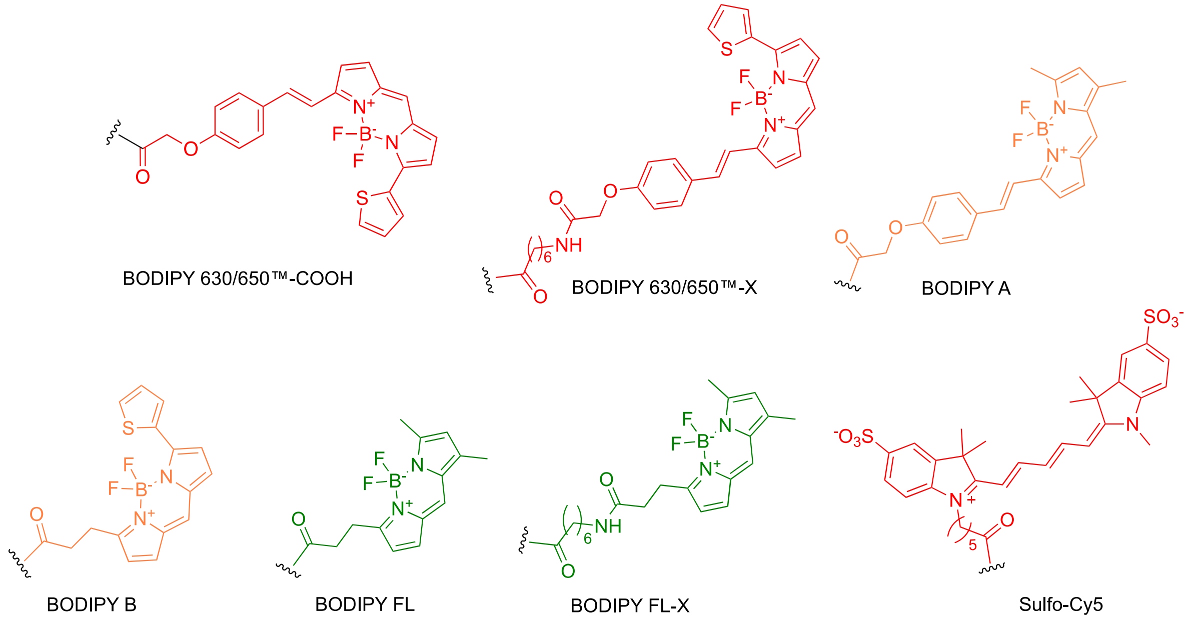

Figure S6. Overview of H_1R fluorescent ligands synthesized.

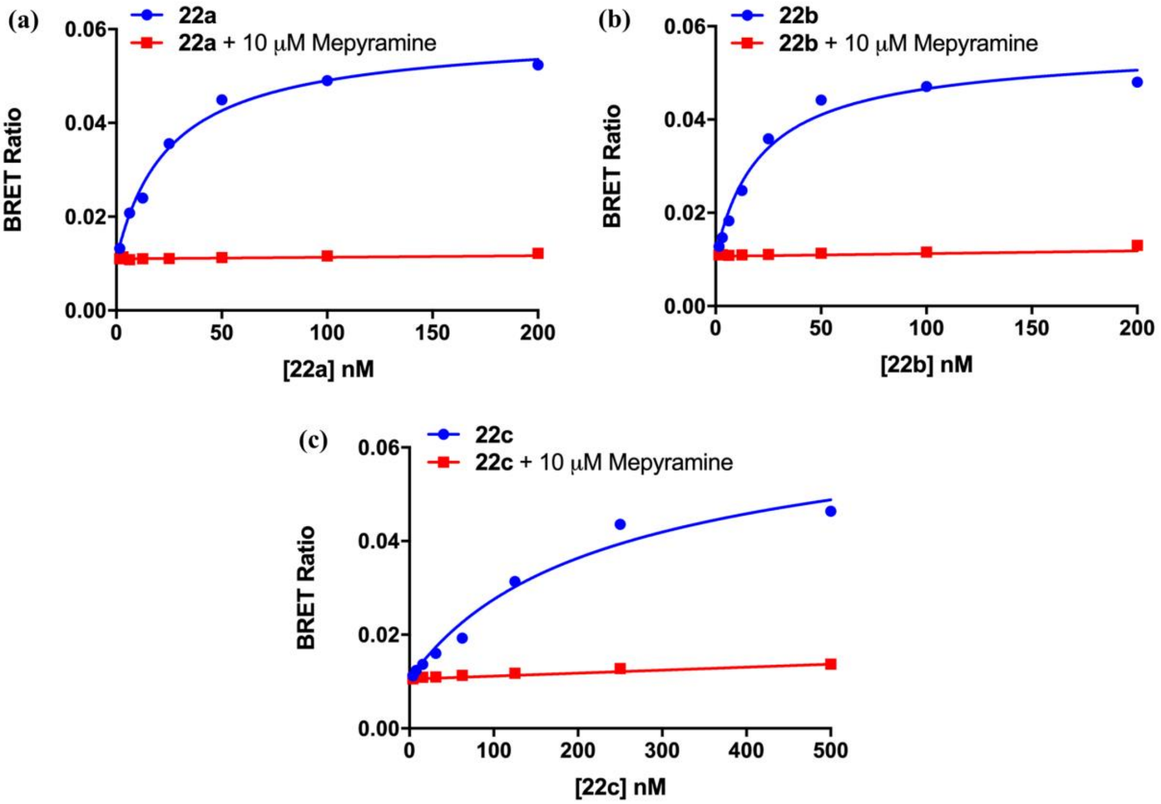

Figure S7. Saturation binding curves of **22a-c** at Nluc-H_1R from NanoBRET assay. The experiment was separately performed using Nluc-H_1R expressing HEK293T cells with increasing concentrations of fluorescent ligands **22a (a)**, **22b (b)** and **22c (c)** in the absence or presence of 10 μM mepyramine. The data shown are representative of five independent experiments performed in triplicate. Data points are expressed in mean ± SEM and error bars are within the limits of the symbols if not shown.

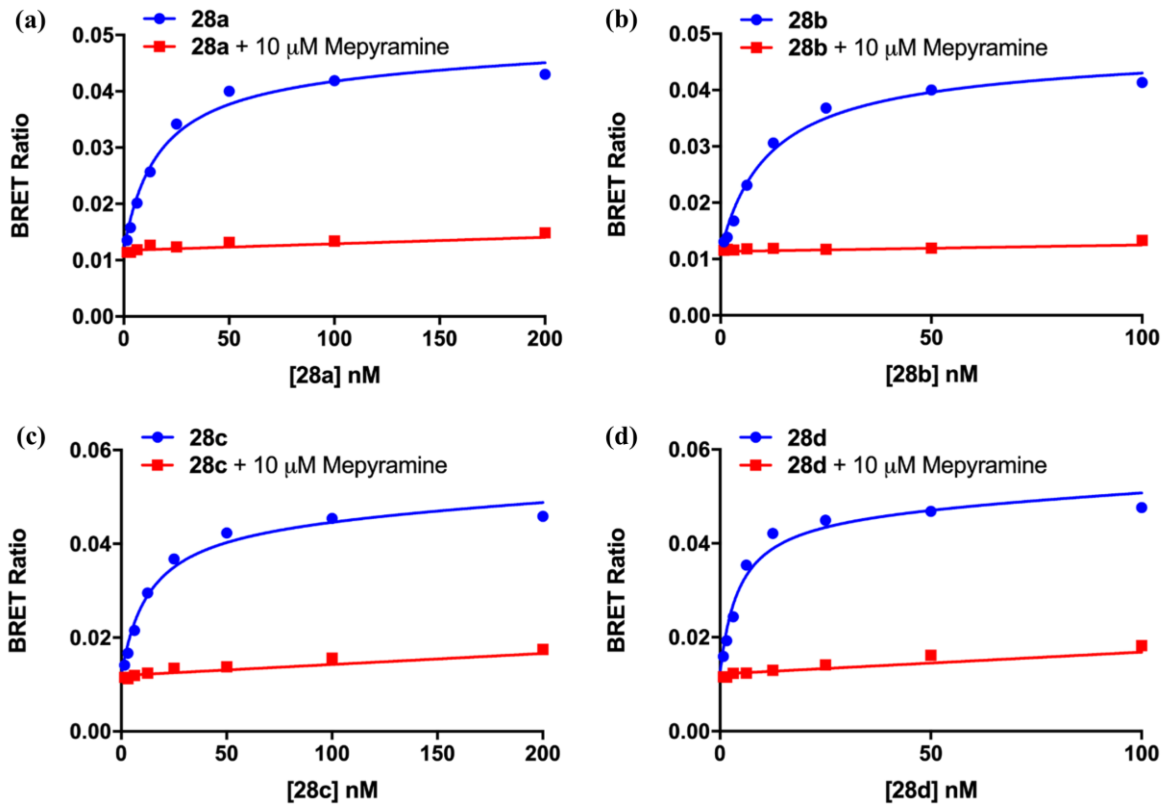

Figure S8. Saturation binding curves of **28a-d** at Nluc-H_1R from NanoBRET assay. The experiment was separately performed using Nluc-H_1R expressing HEK293T cells with increasing concentrations of fluorescent ligands **28a (a)**, **28b (b)**, **28c (c)** and **28d (d)** in the absence or presence of 10 μM mepyramine. The data shown are representative of five independent experiments performed in triplicate. Data points are expressed in mean ± SEM and error bars are within the limits of the symbols if not shown.

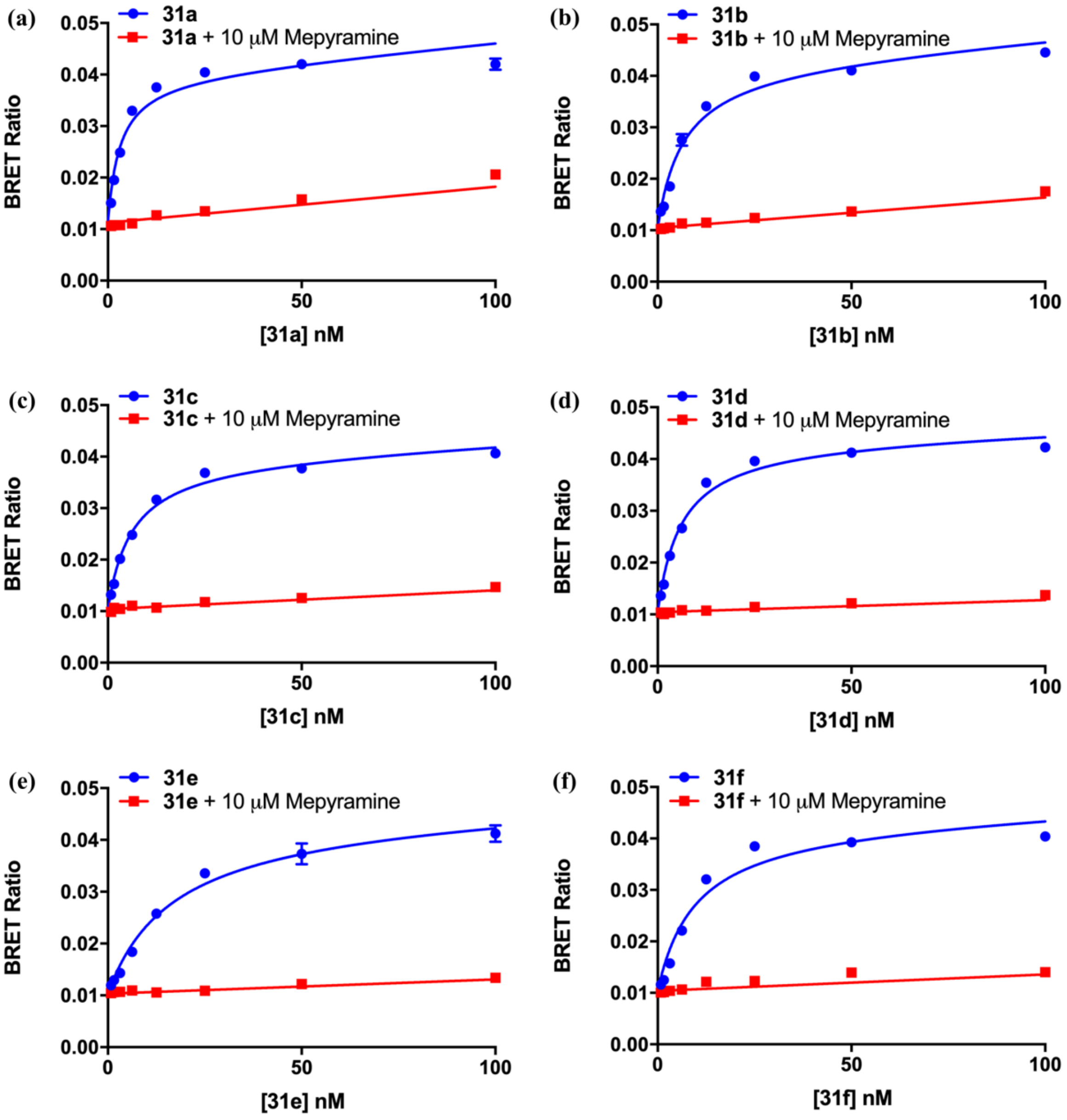

Figure S9. Saturation binding curves of **31a-f** at Nluc-H_1R from NanoBRET assay. The experiment was separately performed using Nluc-H_1R expressing HEK293T cells with increasing concentrations of fluorescent ligands **31a (a)**, **31b (b)**, **31c (c)**, **31d (d)**, **31e (e)** and **31f (f)** in the absence or presence of 10 μM mepyramine. The data shown are representative of four independent experiments performed in triplicate. Data points are expressed in mean ± SEM and error bars are within the limits of the symbols if not shown.

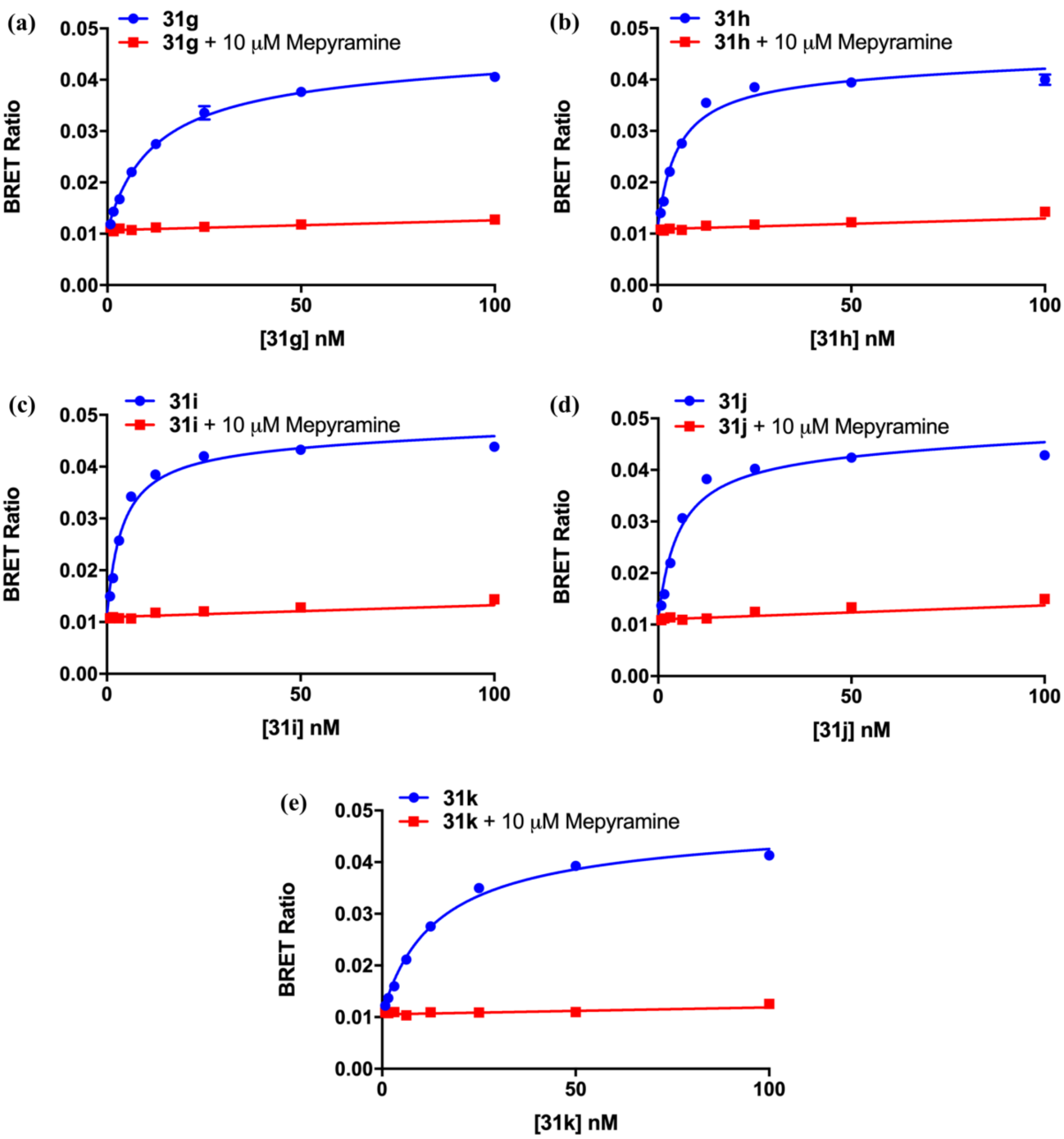

Figure S10. Saturation binding curves of **31g-k** at Nluc-H_1R from NanoBRET assay. The experiment was separately performed using Nluc-H_1R expressing HEK293T cells with increasing concentrations of fluorescent ligands **31g (a)**, **31h (b)**, **31i (c)**, **31j (d)** and **31k (e)** in the absence or presence of 10 μM mepyramine. The data shown are representative of four independent experiments performed in triplicate. Data points are expressed in mean ± SEM and error bars are within the limits of the symbols if not shown.

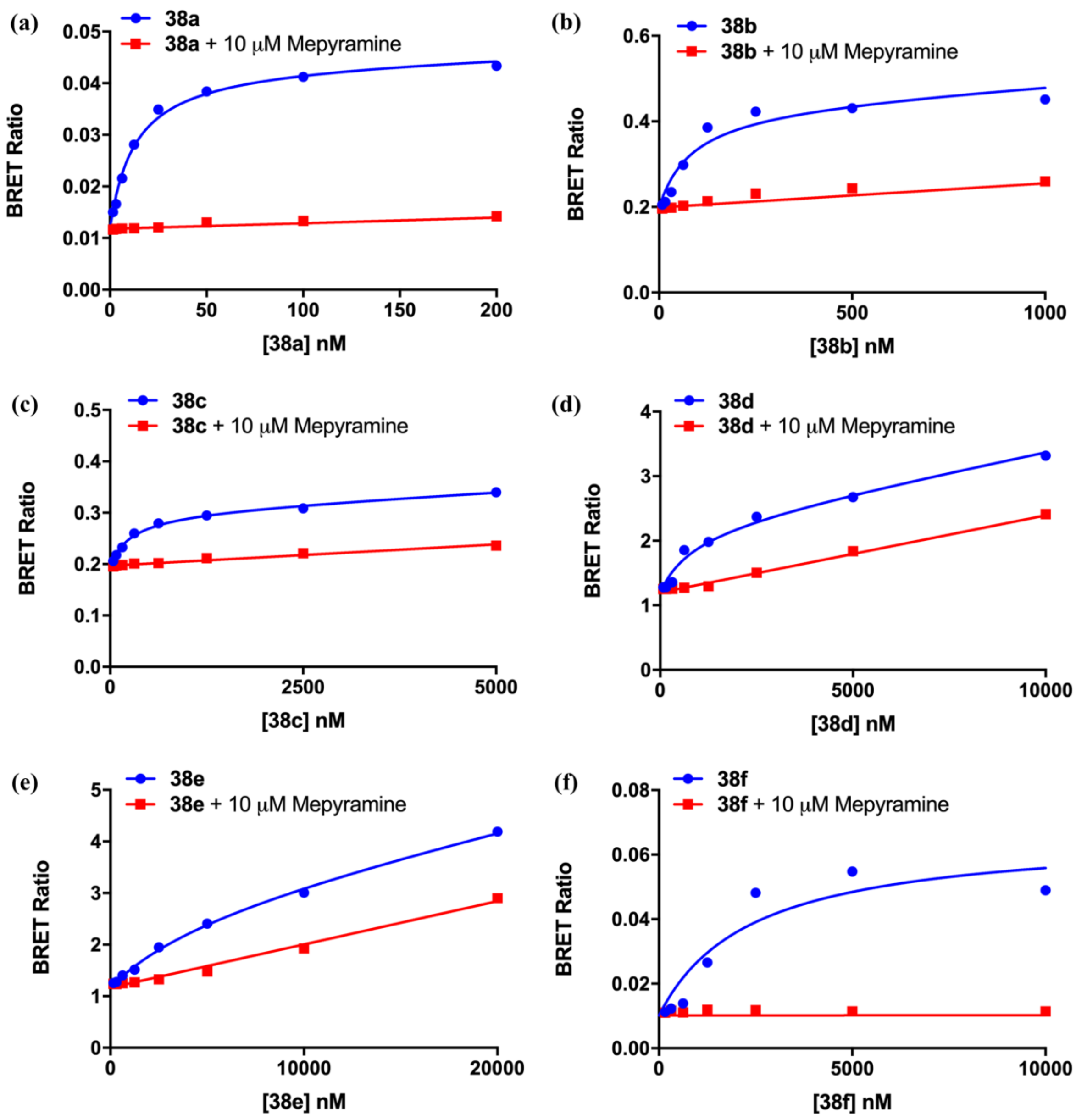

Figure S11. Saturation binding curves of **38a-f** at Nluc-H_1R from NanoBRET assay. The experiment was separately performed using Nluc-H_1R expressing HEK293T cells with increasing concentrations of fluorescent ligands **38a (a)**, **38b (b)**, **38c (c)**, **38d (d)**, **38e (e)** and **38f (f)** in the absence or presence of 10 μM mepyramine. The data shown are representative of four independent experiments performed in triplicate. Data points are expressed in mean ± SEM and error bars are within the limits of the symbols if not shown.

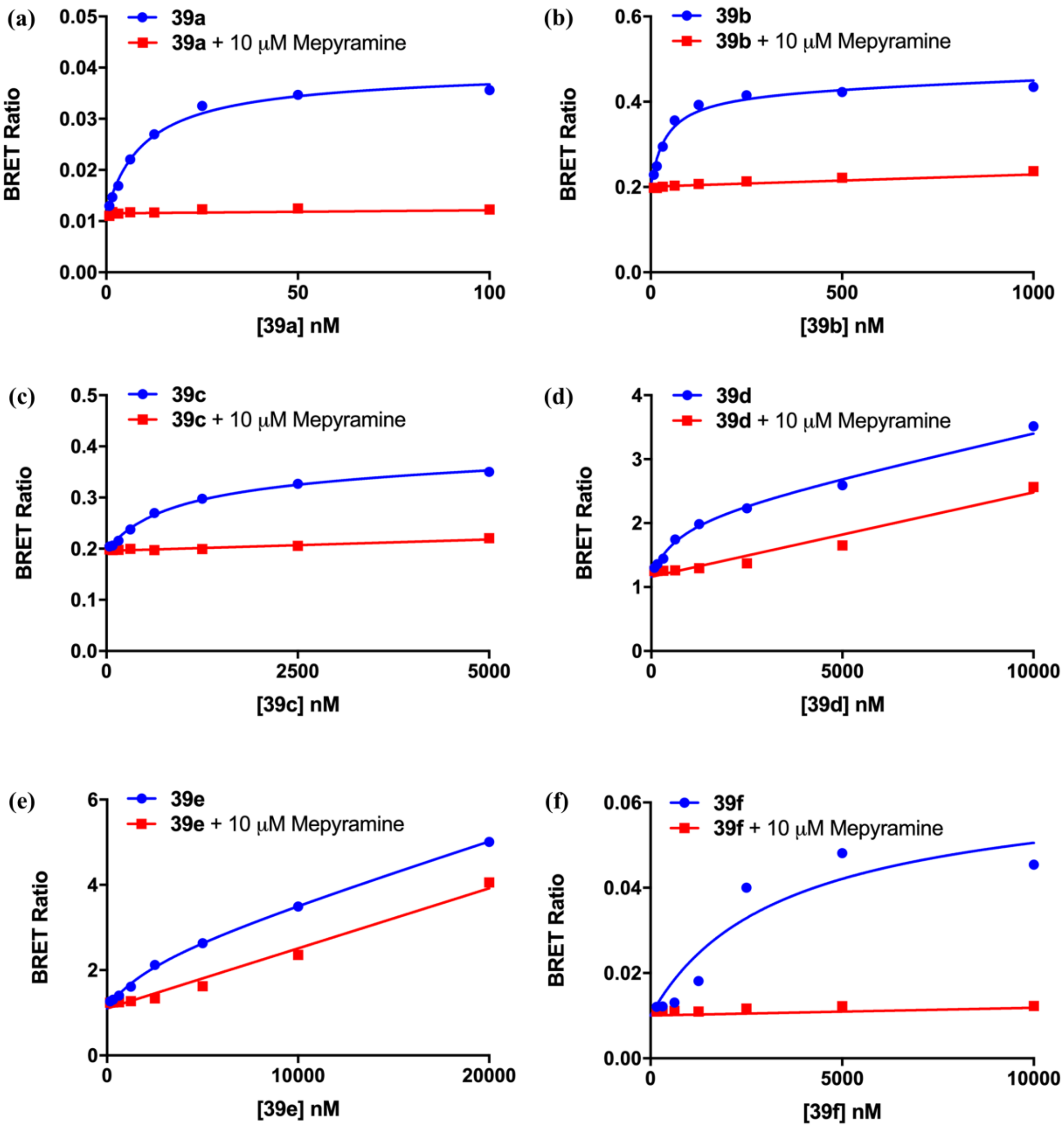

Figure S12. Saturation binding curves of **39a-f** at Nluc-H_1R from NanoBRET assay. The experiment was separately performed using Nluc-H_1R expressing HEK293T cells with increasing concentrations of fluorescent ligands **39a (a)**, **39b (b)**, **39c (c)**, **39d (d)**, **39e (e)** and **39f (f)** in the absence or presence of 10 μM mepyramine. The data shown are representative of four independent experiments performed in triplicate. Data points are expressed in mean ± SEM and error bars are within the limits of the symbols if not shown.

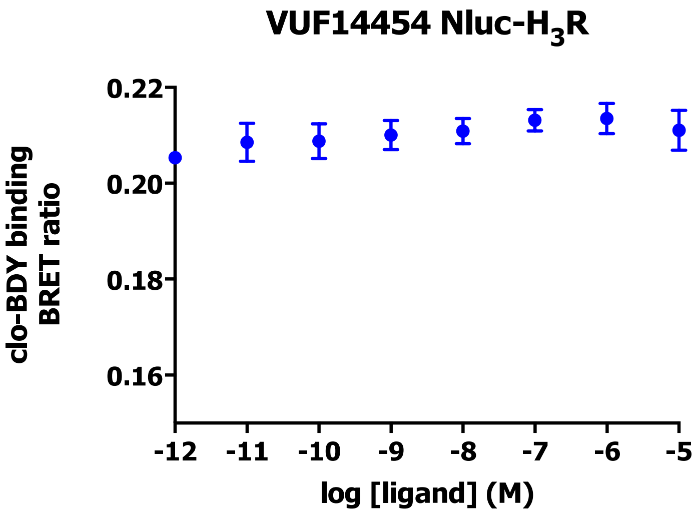

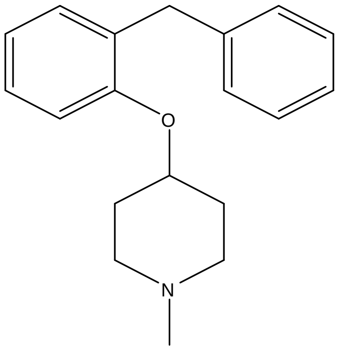

VUF14454

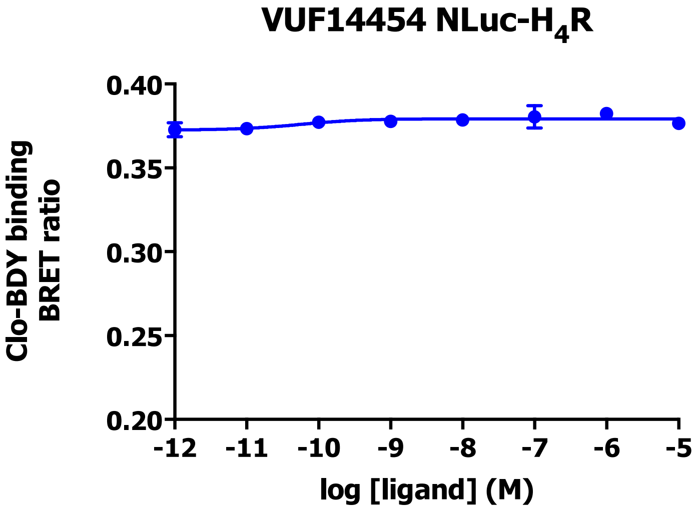

Figure S13. Displacement curve of **VUF14454** at Nluc-H_3R and Nluc-H_4R from NanoBRET assay. The experiments were performed on cell homogenates prepared from Nluc-H_3R and Nluc-H_4R expressing HEK293T cells in the presence of the H_3R/H_4R fluorescent ligand **CA200843** (100 nM) as the labelled competitor in both H_3R and H_4R experiments. **VUF14454** did not displace **CA200843** from both Nluc-H_3R and Nluc-H_4R at concentrations of up to 10 µM. Data shown represent the combined mean ± SEM of three experiments performed in triplicate. The data points are expressed in mean ± SEM and error bars are within the limits of the symbols.

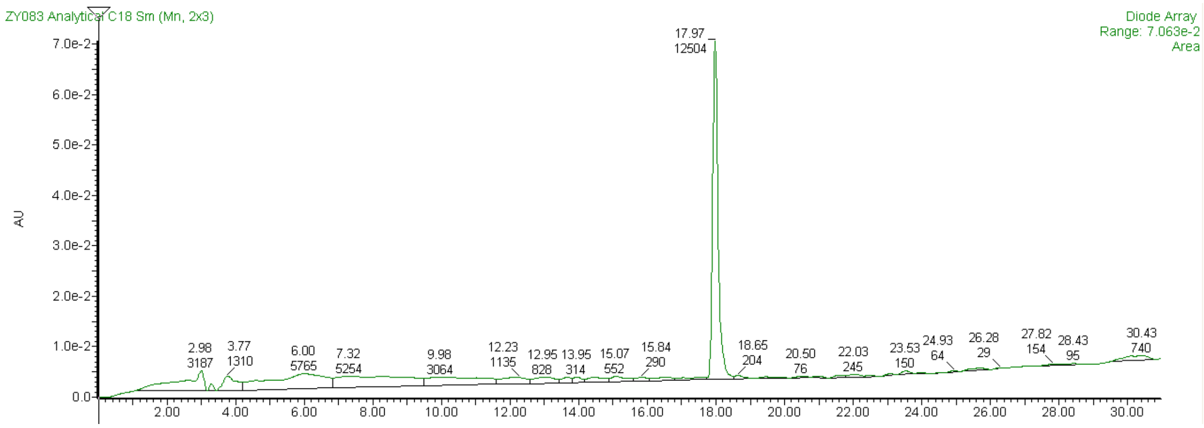

Figure S14. Analytical RP-HPLC chromatogram of **31a**

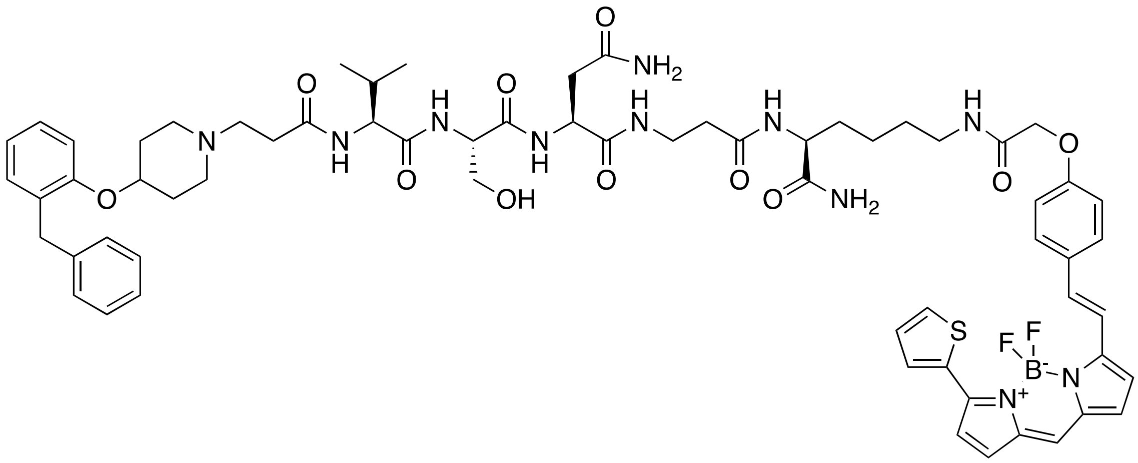

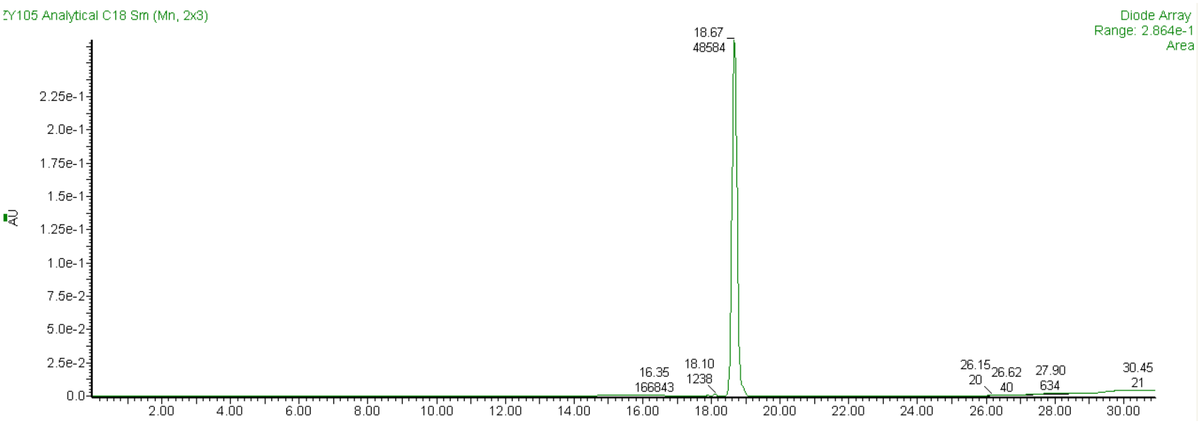

Figure S15. Analytical RP-HPLC chromatogram of **31k**

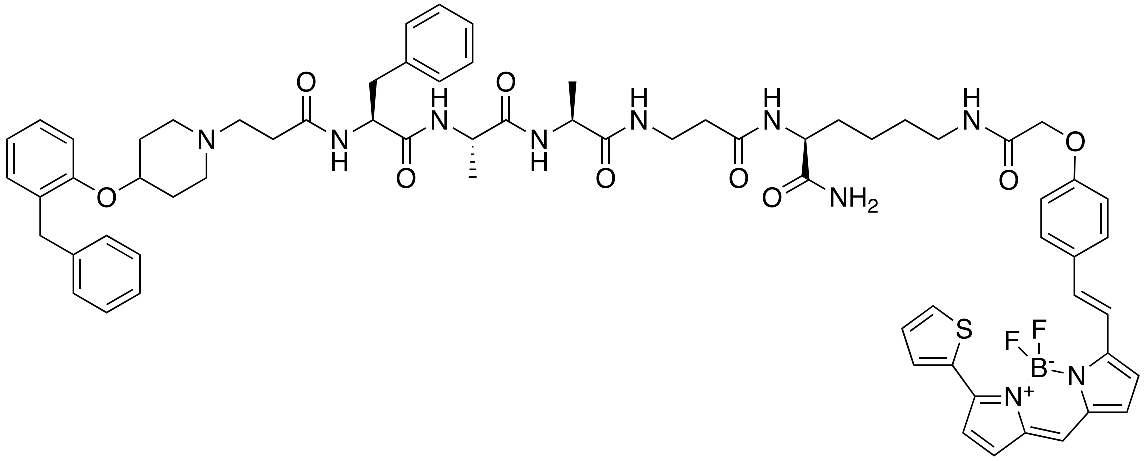

Supplement: Supplementary file 3 — jm2c00125_si_003.pdf [file jm2c00125_si_003.pdf]
